# CRISPR/Cas9‐Based Vanadium MXene‐Free Radical Spatiotemporally Controlled Nanoreactor for Photothermal‐Induced Multi‐Effect Synergistic Antitumor Therapy

**DOI:** 10.1002/advs.202522535

**Published:** 2026-03-12

**Authors:** Zi‐Jian Huang, Feng‐Ming Li, Yi‐Fan Tu, Ke‐Ke Feng, Cheng‐Lei Li, Shi‐Cheng Tian, Yong‐Shan Hu, Jing‐Wei Shao, Zhen‐Hua Liu

**Affiliations:** ^1^ College of Chemistry Fuzhou University Fuzhou Fujian China; ^2^ Department of Oncology Shengli Clinical Medical College of Fujian Medical University Fuzhou University Affiliated Provincial Hospital Fuzhou China

**Keywords:** CRISPR/Cas9, free radicals, immune activation, MXene, photothermal therapy

## Abstract

Photothermal therapy (PTT), a non‐invasive tumor treatment, shows promise but is limited in solid tumors by restricted tissue penetration, thermotolerance, anti‐apoptotic and immunosuppressive effects.

In this study, tumor microenvironment‐responsive nanoplatform VARH was constructed based on MXene. Under NIR‐II laser irradiation, VARH achieves a high photothermal conversion efficiency of 44.21%. Loaded 2,2'‐azobis[2‐(2‐imidazolin‐2‐yl)propane] dihydrochloride decomposes at high temperatures to generate alkyl radicals, synergizing with hydroxyl radicals from V^4+^‐catalyzed endogenous H_2_O_2_ decomposition, enabling chemodynamic therapy (CDT) and thermal dynamic therapy to enhance tumor cell oxidative damage.

Triggered by high glutathione, VARH releases ribonucleoprotein (RNP) complexes to knockout heat shock protein 90 (HSP90), attenuating cellular heat resistance and promoting apoptosis. It also enhances T cell‐mediated anti‐tumor immunity and, with free radicals, promotes tumor cell immunogenic cell death (ICD), achieving immunotherapeutic multi‐effect synergy.

Integrating nanotechnology with precise gene editing, this study develops a novel multimodal synergistic therapy system, providing new insights for multi‐modal treatment R&D and advancing PTT and free radical‐based cancer therapies.

## Graphical abstract

In this study, a MXene‐based V_4_C_3_ nanoplatform, VARH, was constructed, which achieved a photothermal conversion efficiency of 44.21% in the NIR‐II window. This platform integrated photothermal therapy, chemodynamic therapy, and thermodynamic therapy with CRISPR/Cas9‐mediated heat shock protein 90 knock‐out technology. This combination reversed tumor thermotolerance and induced immunogenic cell death, providing a novel paradigm for precise synergistic anti‐tumor therapy.

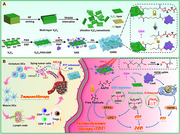

## 1 Introduction

Cancer is one of the leading causes of death worldwide [1]. Its complex heterogeneity, hypoxic microenvironment, and low immunogenicity severely limit the efficacy of existing therapeutic approaches and often lead to poor prognosis and drug‐resistant recurrence. Photothermal therapy (PTT) has demonstrated promising prospects in tumor treatment due to its advantages of excellent spatiotemporal controllability, non‐invasiveness, and low side effects [2]. It utilizes localized hyperthermia to kill tumor cells and, through endoplasmic reticulum stress, induces the release of calreticulin (CRT) and High Mobility Group Box‐1 protein (HMGB1) [3, 4]. This process synergistically promotes the maturation of dendritic cells (DCs), enhances T‐cell immune infiltration, and downregulates the proportion of regulatory T cells (Tregs), thereby facilitating immunogenic cell death (ICD) of tumor cells [5, 6]. However, conventional PTT relies on temperatures above 50°C to achieve tumor ablation, which not only risks thermal damage to surrounding normal tissues but also activates the self‐protective heat stress response in tumor cells. This response involves upregulating the expression of heat shock proteins (HSPs), which can compromise therapeutic efficacy [7, 8]. Furthermore, challenges such as the limited penetration depth of lasers and tumor thermoresistance have hindered its further development.

MXene, as a class of 2D materials, demonstrates broad application potential in fields such as catalysis, sensing, biomedicine and gene editing [9, 10, 11, 12, 13, 14, 15]. Its ultrathin layered structure endows the material with rapid photoresponse characteristics and excellent in‐plane electron mobility, thereby enabling efficient photothermal conversion [16, 17, 18]. Among them, vanadium‐based V_4_C_3_ MXene possesses near‐infrared‐II (NIR‐II) laser responsiveness, which facilitates deep‐tissue photothermal conversion. It also exhibits excellent stability, making it suitable for in vivo therapeutic applications [19]. Concurrently, transition metals (e.g., Mn, Cu, Ti, V) can catalyze the generation of hydroxyl radicals (·OH) from H_2_O_2_ via Fenton‐like reactions, enabling chemodynamic therapy (CDT) at tumor sites [20, 21, 22, 23, 24, 25].

Thermodynamic therapy (TDT), as an emerging tumor treatment strategy, operates by utilizing thermally activated thermosensitive agents to generate reactive oxygen species (ROS) or free radicals within the tumor region. This process induces oxidative stress in tumor cells, leading to mitochondrial dysfunction, DNA damage, and apoptosis. As a non‐oxygen‐dependent free radical generation strategy, TDT enhances local tumoricidal efficacy and promotes ICD, effectively compensating for the limitations of PTT under hypoxic conditions [26, 27]. While some studies have explored the combination of MXene‐based ROS‐generating agents with PTT [28, 29], research integrating MXene, TDT, and gene editing for tumor therapy has not yet formed a mature system.

The combination strategy of hyperthermia and HSP inhibition has been demonstrated to enhance tumor ablation efficacy. Compared to traditional small molecule inhibitors, which suffer from uncontrollable release behavior and significant systemic toxicity, the CRISPR/Cas9 gene editing technology serves as a suitable alternative, offering distinct advantages in reversing tumor drug resistance and precisely regulating gene expression [30, 31, 32, 33]. This technology can precisely inhibit the expression of HSPs, thereby disrupting tumor thermotolerance at its source, which improves the efficacy of PTT and reduces side effects [34].

Due to their metabolic abnormalities and genetic instability, tumor cells are more susceptible to oxidative stress. Disrupting their redox homeostasis using active free radicals has emerged as a promising therapeutic strategy [35, 36]. However, the hypoxic microenvironment commonly present in solid tumors not only promotes tumor metastasis and proliferation but also severely limits the efficacy of oxygen‐dependent free radical therapies [37, 38]. This further underscores the importance of synergistic treatment combining TDT and PTT.

**SCHEME 1:**
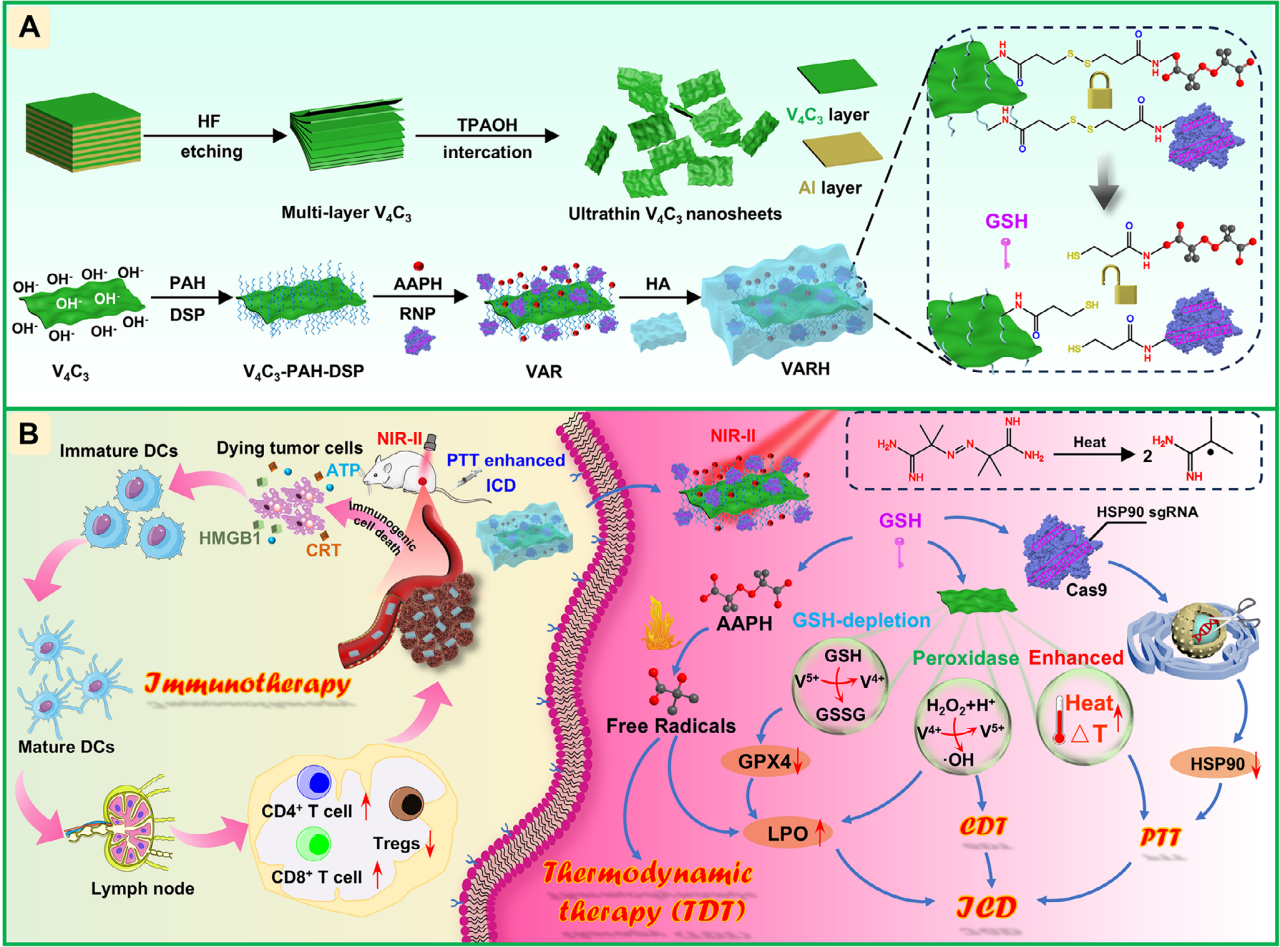
Synthesis and mechanism of VARH. (A) Fabrication of VARH. (B) Schematic diagram of VARH treatment process.

Based on the aforementioned background, this study employed poly(allylamine hydrochloride) (PAH) to aminate V_4_C_3_ nanosheets. Through amide reactions, V_4_C_3_, 2,2'‐azobis(2‐methylpropionamidine) dihydrochloride (AAPH), and the heat shock protein 90 (HSP90)‐targeting RNP complex were conjugated to construct nanoparticles (AAPH+RNP)/ V_4_C_3_ (VAR) with glutathione (GSH)‐responsive release and lysosomal escape capabilities. Subsequently, hyaluronic acid (HA) was coated via electrostatic adsorption, ultimately yielding (AAPH+RNP)/ V_4_C_3_@HA (VARH) with excellent stability and prominent targeting properties (Scheme 1). This composite system can be internalized into tumor cells via HA‐mediated targeting, achieve lysosomal escape through the proton sponge effect, and trigger the release of AAPH and RNP in the intracellular high‐GSH environment. RNP precisely knocks down the HSP90 gene, reversing tumor thermoresistance. Under NIR‐II laser irradiation, V_4_C_3_ exerts photothermal conversion functions while simultaneously triggering AAPH to generate alkyl radicals (·R) in situ, which oxidatively damage tumor cells. This achieves multimodal synergistic therapy, induces tumor cell necrosis/apoptosis, and releases damage‐associated molecular patterns (DAMPs), thereby activating ICD. By integrating multiple synergistic modes of PTT, CDT, TDT, and gene editing, this nanosystem provides a novel interventional strategy to overcome the challenges of cancer therapy.

## 2 Materials and Methods

### 2.1 The Main Reagents and Materials

V_4_AlC_3_ powder was purchased from Xinxi Technology Co., Ltd. (Xi'an, China). Poly(allylamine hydrochloride) (PAH, molecular weight ≈ 15 000) (98%) was obtained from Shanghai Yuanye Bio‐Technology Co., Ltd. (China). Hydrofluoric acid (HF), tetrapropylammonium hydroxide (TPAOH), 3,3'‐Dithiobis(succinimidylpropionate) [crosslinker] (DSP), 2,2'‐Azobis(2‐methylpropionamidine) dihydrochloride (AAPH), reduced glutathione (GSH), methylene blue (MB) were all purchased from Aladdin Biochemical Technology Co., Ltd. (Shanghai, China). Hoechst 33342 (CB2771) was obtained from Beijing Coolaber Science&Technology Co., Ltd. (China). LysoTracker Red and the DCFH‐DA assay kit were purchased from Dalian Meilun Biotechnology Co., Ltd. (Dalian, China). Anti‐HSP90 antibody was sourced from Wanlei Biotechnology Co., Ltd. (Shenyang, China). The lipid peroxidation sensor C11‐BODIPY^581/591^ was acquired from Qifeng Biotechnology Co., Ltd. (Shanghai, China). The Calcein AM/PI Double Staining Kit was obtained from Solarbio. (Beijing, China). Rhodamine 123 dye and the Annexin V‐FITC Apoptosis Detection Kit were purchased from Beyotime Biotechnology (Shanghai, China). The Modified Hematoxylin‐Eosin (HE) Staining Kit was a product of Solarbio. (Beijing, China). Anti‐CRT antibody, Anti‐HMGB1 antibody, and Alexa Fluor 488‐conjugated goat anti‐rabbit IgG secondary antibody were purchased from ImmunoWay Biotechnology Company (USA). Assay kits for alanine aminotransferase (ALT/GPT), aspartate aminotransferase (AST/GOT), and blood urea nitrogen (BUN) were obtained from Beijing Boxbio Science & Technology Co., Ltd. (China). Anti‐mouse CD3‐PE, CD4‐FITC, CD8‐APC, CD11C‐APC, CD80‐FITC, CD86‐PE, and Foxp3‐FITC antibodies were all purchased from Elabscience Biotechnology Inc. (Wuhan, China). The Mouse tumor necrosis factor‐α (TNF‐α), interferon‐γ (IFN‐γ), and interleukin‐6 (IL‐6) Enzyme‐Linked Immunosorbent Assay (ELISA) kit was purchased from Jiangsu Meimian Industrial Co., Ltd. (China). The sgRNA for HSP90α (Table S1) was chemically synthesized by GenScript Biotech Co., Ltd. (Nanjing, China).

### 2.2 Characterizations

Atomic force microscopy (AFM) images were acquired using a Nanoscope Multi Mode VIII system (Bruker, Germany). Transmission electron microscopy (TEM) images were obtained with a TECNAI G2 F20 system (FEI, USA). Dynamic light scattering (DLS) and zeta potential measurements were performed on a Nano ZS90 laser particle size analyzer (Malvern, UK). X‐ray photoelectron spectroscopy (XPS) was recorded on an Escalab 250 photoelectron spectrometer (Thermo Scientific, USA). Ultraviolet‐visible‐near‐infrared (UV‐Vis‐NIR) spectra were collected using a Lambda 950 spectrophotometer (PerkinElmer, USA). Near‐infrared‐II (NIR‐II) laser irradiation was applied using an LSR‐1064‐NL 1064 nm pumped solid‐state laser (Yuanming, Ningbo, China). Temperature changes and thermal images were monitored and captured with a Fotric 323 Pro#L25 infrared thermal imager (FOTRIC, China). Electron spin resonance (ESR) spectra were acquired on an A300 spectrometer (Bruker, Germany). Confocal laser scanning microscopy (CLSM) images were captured using a Leica TCS SP8 system (Leica, Germany).

### 2.3 Synthesis and Optimization of V_4_C_3_‐PAH‐DSP

One gram of V_4_AlC_3_ was weighed and added to 50 mL of HF for etching. After centrifugation, the supernatant was discarded, and the precipitate was washed with deionized water until a neutral pH was achieved. Subsequently, 10 mL of TPAOH solution was added to the precipitate, followed by stirring for 24 h. The mixture was then centrifuged at 12 000 rpm for 10 min, and the resulting precipitate was collected. The precipitate was dispersed in deionized water and subjected to freeze‐drying to obtain monolayer V_4_C_3_. The obtained V_4_C_3_ solid was weighed, dispersed in deionized water, and ultrasonicated using a ultrasonic disruptor in an ice‐water bath for 8 h to prepare a 1 mg/mL V_4_C_3_ stock solution. According to the optimal V_4_C_3_‐to‐PAH ratio, 2 mL of the V_4_C_3_ stock solution was mixed with 4 mL of PAH solution (1 mg/mL) and stirred at room temperature for 2 h. The mixture was centrifuged using a 100 kDa ultrafiltration tube, and the resulting concentrate was dispersed in dimethyl sulfoxide (DMSO) to obtain a V_4_C_3_‐PAH dispersion. Subsequently, 1 mL of DSP solution (4.5 mg/mL) was added, and the reaction was stirred at room temperature for 4 h. The product was purified again via ultrafiltration and finally dispersed in HEPES buffer (20 mm) for subsequent use.

### 2.4 Preparation of Various Nanocomplexes

VH: VH was prepared by stirring 2 mL of V_4_C_3_ dispersion with 400 µL of HA solution (10 mg/mL) for 30 min.

VAH: To 2 mL of V_4_C_3_‐PAH‐DSP, 1 mL of AAPH solution (12 mg/mL) was added, and the mixture was stirred at 4°C for 8 h. After ultrafiltration, 400 µL of HA solution (10 mg/mL) was added, and stirring was continued for another 30 min.

VAR: A RNP complex was formed by incubating 3 µL of sgRNA (2 µg/µL), 12 µL of Cas9 nuclease (2 µg/µL), and 45 µL of 1 × Cas9 buffer at room temperature for 30 min. Subsequently, this RNP complex was added to 2 mL of V_4_C_3_‐PAH‐DSP and stirred at room temperature for 2 h. Then, 1 mL of AAPH in HEPES buffer (8 mg/mL) was added, and the mixture was stirred at 4°C for 8 h. Finally, the product was purified sequentially via 100 kDa ultrafiltration and 300 kDa dialysis.

VARH: To the VAR dispersion, 400 µL of HA solution (10 mg/mL) was added and stirred for 30 min. After ultrafiltration centrifugation, the final product was dispersed in deionized water.

### 2.5 Encapsulation and Drug Release Studies

Standard curves were established based on UV absorption of ABTS^+^• and EGFP fluorescence intensity. The preparation was optimized by adjusting the feed ratio of RNP to AAPH. After removing unloaded drugs via ultrafiltration and dialysis, the actual drug content was determined to calculate the encapsulation efficiency (EE) and load efficiency (LE). To evaluate stability, VARH was dispersed in ultrapure water, PBS, 10% glucose, and cell culture medium, stored at 4°C, and periodically monitored for particle size and physical state. Additionally, the GSH‐responsive release behavior was investigated by dialyzing VARH (with V_4_C_3_ concentration of 1 mg/mL) in pH 7.4 buffer (without GSH) and pH 5.5 buffer (containing 10 mm GSH). Samples of 1 mL were collected at 0, 1, 2, 4, 8, 12, and 24 h under oscillation at 37°C and 200 rpm, with an equal volume of fresh buffer replenished each time. The cumulative release was calculated, and release profiles were plotted.

### 2.6 Photothermal Conversion Performance

To systematically evaluate the photothermal performance, dispersions of V_4_C_3_ and VARH were first diluted with ultrapure water to prepare gradient samples with V_4_C_3_ concentrations of 12.5, 25, 50, 100, and 200 µg/mL. Using ultrapure water as a control, these samples were irradiated with a 1064 nm laser at a power density of 1.0 W/cm^2^ for 5 min, and the temperature changes were recorded. Subsequently, with the V_4_C_3_ concentration fixed at 50 µg/mL, the temperature increase was investigated under power densities ranging from 0.5 to 1.5 W/cm^2^. Photostability was assessed through five laser on‐off cycles (5 min irradiation / 15 min cooling). Finally, 1 mL of the 50 µg/mL VARH sample was irradiated with the laser (1.0 W/cm^2^) until the temperature reached equilibrium, after which the laser was turned off to allow natural cooling; deionized water was used as a control. The photothermal conversion efficiency (η) was calculated based on the temperature data.

### 2.7 Ex Vivo Detection of Radical Generation

To detect ·OH, a solution containing MB (20 µg/mL) and H_2_O_2_ (1 mm) was mixed with VARH (100 µg/mL V_4_C_3_) at a 1:1 ratio. The change in UV absorbance was compared between the group irradiated with a 1064 nm laser for 5 min and the non‐irradiated control group. For the detection of ∙R, ABTS (2 mg/mL) was mixed with VARH, and the absorbance was monitored under different conditions: incubation in water baths at 37°C and 44°C, and under laser irradiation. To record the ESR signal, a mixture of 200 µg/mL V_4_C_3_/VARH and 100 mm DMPO was subjected to laser irradiation (1.0 W/cm^2^), and the signal was subsequently recorded using a Bruker X‐band A300 Electron Spin Resonance spectrometer.

### 2.8 Cellular Uptake and Lysosomal Escape

Both cellular uptake and lysosomal escape assays were performed using HepG2 cells. For the uptake assay, qualitative and quantitative analyses were conducted via CLSM and flow cytometry (FCM), respectively. After 24 h of cell culture, ICG‐labeled VARH was added and incubated in the dark at different time points (0–8 h). Cellular uptake was qualitatively observed using CLSM, while cells in the FCM group were trypsinized, resuspended, and subsequently analyzed. For the lysosomal escape assay, cells were seeded on coverslips and cultured for 24 h. EGFP‐labeled VARH was added 0–8 h prior to detection. Subsequently, lysosomes were stained with LysoTracker Red, followed by fixation and nuclear staining with Hoechst 33342. Colocalization was then observed by CLSM.

### 2.9 Assessment of Gene Editing Efficiency

Gene editing efficiency was evaluated by immunofluorescence and the T7 endonuclease I (T7EI) cleavage assay. HepG2 cells were seeded on coverslips and cultured for 24 h, followed by treatments assigned to the Control, VH, VARH, VH+L, VAH+L, and VARH+L groups. The cells were subsequently observed using CLSM. For the T7EI assay, HepG2 cells were cultured for 24 h and then treated with Control, VH, VAH, or VARH for another 24 h. Genomic DNA was extracted, and the target gene fragments were amplified by polymerase chain reaction (PCR) using primers detailed in Table S1. The PCR products were digested with T7EI enzyme and subjected to agarose gel electrophoresis. The band intensities were analyzed using ImageJ software to determine the editing efficiency.

### 2.10 Cytotoxicity and Live/Dead Assays

The cytotoxicity of the nanomaterials was systematically evaluated using the MTT assay, CCK‐8 assay, and Calcein‐AM/PI double staining. The MTT assay was used for adherent HepG2 cells, while the CCK‐8 assay was applied to suspended H22 cells. After treatment with drug‐containing medium for 24 h, the groups designated for light exposure were irradiated with a 1064 nm laser (1.0 W/cm^2^, 5 min) and then incubated for an additional 4 h. Cell viability was determined by measuring MTT formazan formation or the CCK‐8 reaction, respectively. Concurrently, a Calcein‐AM/PI live/dead cell staining experiment was performed. HepG2 cells were cultured under normoxic conditions or under a hypoxic environment simulated by mineral oil overlay. Following respective treatments in different groups, the cells were stained with Calcein‐AM/PI for 30 min, and cell viability was observed using fluorescence microscopy.

### 2.11 Intracellular Detection

To systematically evaluate the oxidative stress, apoptosis, and ICD effects induced by VARH nanoparticles at the cellular level, HepG2 cells were used. Laser‐irradiated and non‐irradiated cells were assessed for intracellular radical generation using the DCFH‐DA probe. Lipid peroxidation levels were monitored using the C11‐BODIPY^581/591^ probe. Changes in mitochondrial membrane potential were detected using the Rhodamine 123 probe, analyzed by both CLSM and FCM. Apoptosis was analyzed by FCM using the Annexin V‐FITC/PI double staining method. The expression and localization of ICD markers, CRT and HMGB1, were detected via immunofluorescence.

### 2.12 Ethics Approval and Consent to Participate

All animals used in experiments are handled in accordance with the procedures approved by the Experimental Animal Ethics Committee, College of Biological Science and Engineering, Fuzhou University (Approval number: 2024‐SG‐063).

### 2.13 Establishment of the Tumor‐Bearing Mouse Model

ICR mice (male, 3–8 weeks old, SPF grade) were purchased from Fuzhou Wushi Laboratory Animal Co. (China), Ltd. A 100 µL suspension of H22 cells (2 × 10^7^ cells/mL) was subcutaneously injected into the right hind limb of ICR mice. When the tumor volume exceeded 50 mm^3^, the mice were randomly assigned to groups for animal experiments. For the treatment and in vivo photothermal experiments (PBS+L group, VAR+L group), five mice per group were used. For the in vivo imaging experiment, three mice per group were used.

### 2.14 In Vivo Distribution and Photothermal Imaging of VARH

To evaluate the in vivo distribution and photothermal performance of VARH, tumor‐bearing mice were intravenously injected with ICG‐labeled VAR and VARH (ICG dose: 0.5 mg/kg). The mice were anesthetized for in vivo fluorescence imaging at 1, 2, 4, 8, 12, 24, and 48 h post‐injection. After 48 h, the mice were euthanized, and major organs and tumor tissues were collected for ex vivo imaging analysis. For photothermal imaging, VH, VAR, VAH, and VARH (V_4_C_3_ dose: 10 mg/kg) were administered intravenously, with a PBS group as control. At 24 h post‐injection, the tumor region was irradiated with a 1064 nm laser (1.0 W/cm^2^) for 5 min, and temperature changes were monitored in real‐time using an infrared thermal imaging camera.

### 2.15 In Vivo Anti‐Tumor Efficacy and Mechanistic Studies of VARH

To assess the in vivo anti‐tumor efficacy, safety, and mechanism of action of VARH, H22 tumor‐bearing mice were randomly divided into the following groups: Control, VH, VARH, VH+L, VAH+L, and VARH+L. Nanoparticles (V_4_C_3_ concentration: 10 mg/kg) or an equivalent volume of PBS were administered intravenously on days 0, 2, 4, 6, and 8. 24 h after each administration, the tumor regions in the “ laser‐treated groups” groups were exposed to 1064 nm laser irradiation (1.0 W/cm^2^, 5 min). Tumor volume was measured every 2 days. When the tumor volume in control group tumor‐bearing mice exceeded 1500 mm^3^ or body weight loss was greater than 20%, mice in each group were euthanized. On day 14, tumors and major organs were harvested for histological H&E staining, weighing, and calculation of the tumor inhibition rate. Biocompatibility was further evaluated by incubating a 4% red blood cell suspension with serial concentrations of VARH for 4 h, followed by measurement of absorbance at 540 nm and serum biochemical analysis (ALT, AST, BUN). Finally, immunofluorescence was employed to observe the expression of HSP90, CRT, and HMGB1 in tumor tissues. Flow cytometric analysis was performed to quantify tumor‐infiltrating mature dendritic cells, CD4^+^ and CD8^+^ T cells, and Tregs cells. Serum levels of cytokines (TNF‐α, IFN‐γ, IL‐6) were determined by ELISA.

### 2.16 Statistical Analysis

Experimental data were statistically analyzed using Origin software and are presented as the mean ± standard deviation (Mean ± SD). A paired t‐test was used to analyze the significance between two experimental groups. Differences were considered statistically significant at *p < 0.05, **p < 0.01, ***p < 0.001, and ****p < 0.0001. *n* = 6 per group for in vivo experiments; *n* = 3 biological replicates for in vitro experiments.

## 3 Results and Discussion

### 3.1 Preparation and Characterization of VAR and VARH

**FIGURE 1:**
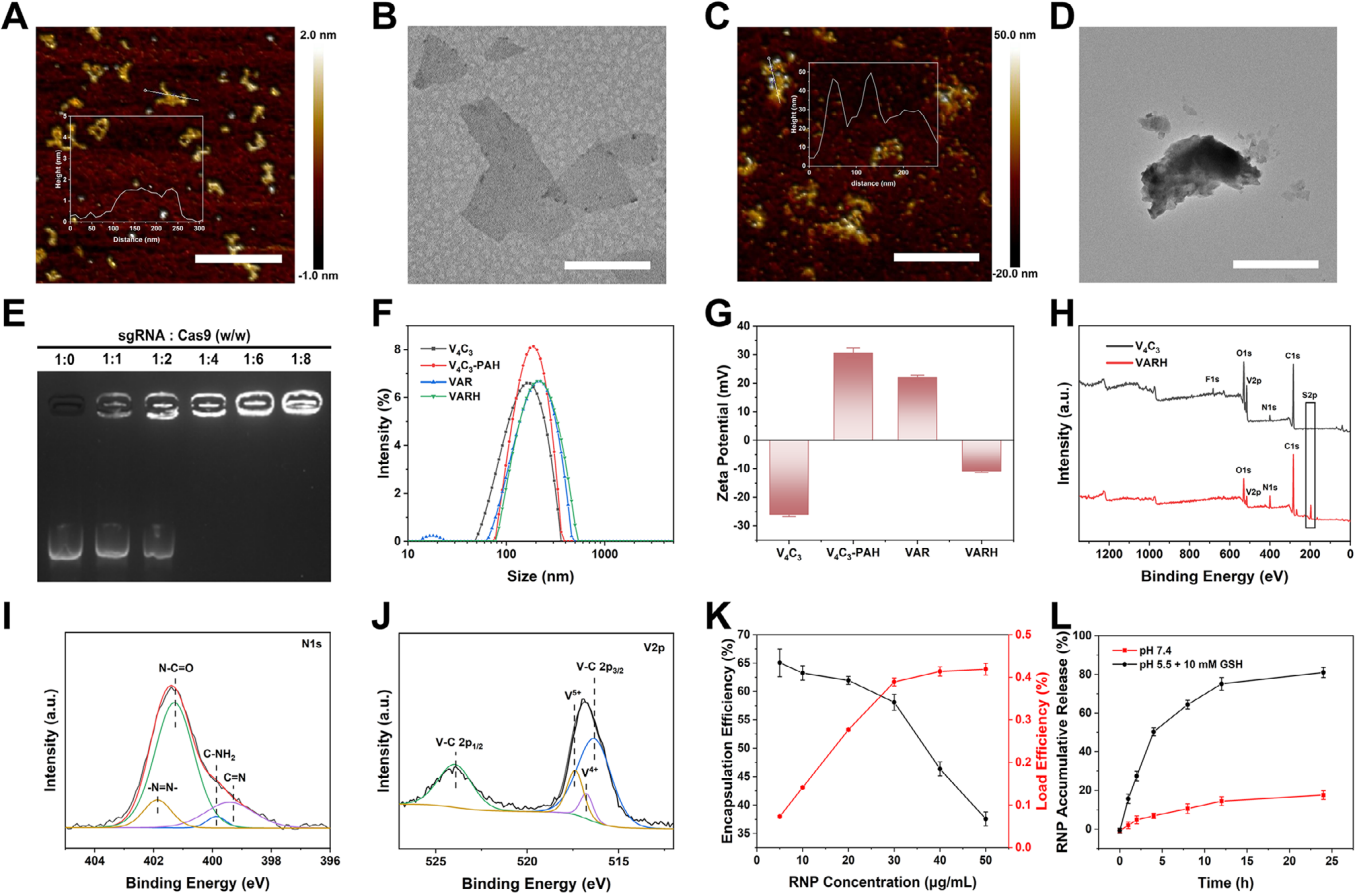
Synthesis and characterization of VARH. AFM images and thickness comparison of V_4_C_3_ (A) and VARH (C), scale bar = 500 nm. TEM images of V_4_C_3_ (B) and VARH (D), scale bar = 200 nm. (E) Agarose gel electrophoresis of RNP complexes prepared with different mass ratios of sgRNA and Cas9. (F) Particle size distribution and (G) Zeta potential of V_4_C_3_, V_4_C_3_‐PAH, VAR, and VARH. (H) Comparison of full XPS Spectra of V_4_C_3_ and VARH. (I) Nitrogen element XPS spectrum of VARH. (J) Vanadium element XPS spectrum of VARH. (K) Effects of different feeding amounts on RNP complex EE and LE. (L) Release of RNP in VARH under different environments (pH 7.4/pH 5.5 + 10 mm GSH).

The synthesis of VAR and VARH was conducted through a multi‐step process. Initially, the Al layers in the V_4_AlC_3_ MAX phase were etched using HF to obtain multilayered V_4_C_3_ which was subsequently intercalated and subjected to ultrasonication to yield monolayer V_4_C_3_ nanosheets with a uniform thickness of approximately 1.5 nm and homogeneous dimensions (Figure 1A,B). Subsequently, PAH was electrostatically adsorbed onto the nanosheet surfaces. At a V_4_C_3_‐to‐PAH mass ratio of 1:2 (Table S2), nanosheets with appropriate particle size and charge reversal were obtained. This step also introduced amino groups, which reacted with one end of the DSP crosslinker. The other end of DSP was then coupled via amide bonds to amino groups present on either AAPH or the RNP complex, resulting in positively charged VAR nanocomposites. Finally, HA coating was applied to achieve charge reversal, yielding VARH. Characterization by Figure 1C,D revealed an average thickness of approximately 25 nm and lateral dimensions of about 200 nm.

The RNP complex was prepared by mixing sgRNA and Cas nuclease at a molar ratio of 1:4. Agarose gel electrophoresis (Figure 1E) confirmed optimal complex formation at this ratio. Particle size and zeta potential analyses indicated that the ultrasonicated V_4_C_3_ had a size of 146.9 ± 0.3 nm, which increased to 213.8 ± 0.2 nm for the final VARH, consistent with AFM and TEM results. The zeta potential of V_4_C_3_ was initially –25.9 mV, which reversed to +30.7 mV after PAH modification. It remained positive after loading with AAPH and RNP, and finally reversed to –10.8 mV following HA coating (Figure 1F,G).

XPS analysis revealed the appearance of an S2p peak in VARH, indicating successful grafting of DSP containing disulfide bonds (─S─S─) (Figure 1H). Compared with the N 1s spectrum of V_4_C_3_ (Figure S1A), signals corresponding to azo bonds (─N═N─) and amide bonds were observed in VARH (Figure 1I), confirming the successful loading of AAPH and effective coupling via DSP. Elemental analysis further confirmed the presence of C, O, and other elements (Figure S1B,C). The appearance of the V^4+^ peak in the V element spectrum (Figure 1J and Figure S2) suggested that VARH possesses the capability to catalyze H_2_O_2_ to generate ·OH.

Using the reaction in which ∙R generated from thermal decomposition of AAPH react with ABTS to form ABTS^+^ [39, 40], a standard curve was constructed based on ultraviolet absorption at different AAPH concentrations (Figures S3 and S4). When the feeding concentration of AAPH was 10 mg/mL, the EE and LE reached 22.18% ± 1.24% and 28.30% ± 1.13%, respectively, approaching saturation (Figure S5). Subsequently, a standard curve for the RNP complex was established based on EGFP fluorescence intensity (Figure S6). As shown in Figure 1K, when the RNP concentration was 30 µg/mL, the EE and LE were 58.09% ± 1.41% and 0.39% ± 0.01%, respectively. All subsequent experiments were performed using the aforementioned drug concentrations.

In a simulated tumor microenvironment (pH 5.5, 10 mm GSH), the disulfide bonds in VARH were cleaved, leading to substantial release of RNP and AAPH. After 24 h, the cumulative release rates of AAPH and RNP reached 52.61% ± 1.21% (Figure S7) and 80.98% ± 2.60% (Figure 1L), respectively, significantly higher than those observed under normal physiological conditions (pH 7.4).

Over a period of 5 days, the particle size of VARH increased from 211.3 to 269.7 nm, accompanied by a pronounced Tyndall effect (Figure S8). VARH remained uniformly dispersed in various media after 5 days (Figure S9), indicating favorable stability and biocompatibility.

### 3.2 Photothermal Properties and Free Radical Release of VARH

**FIGURE 2:**
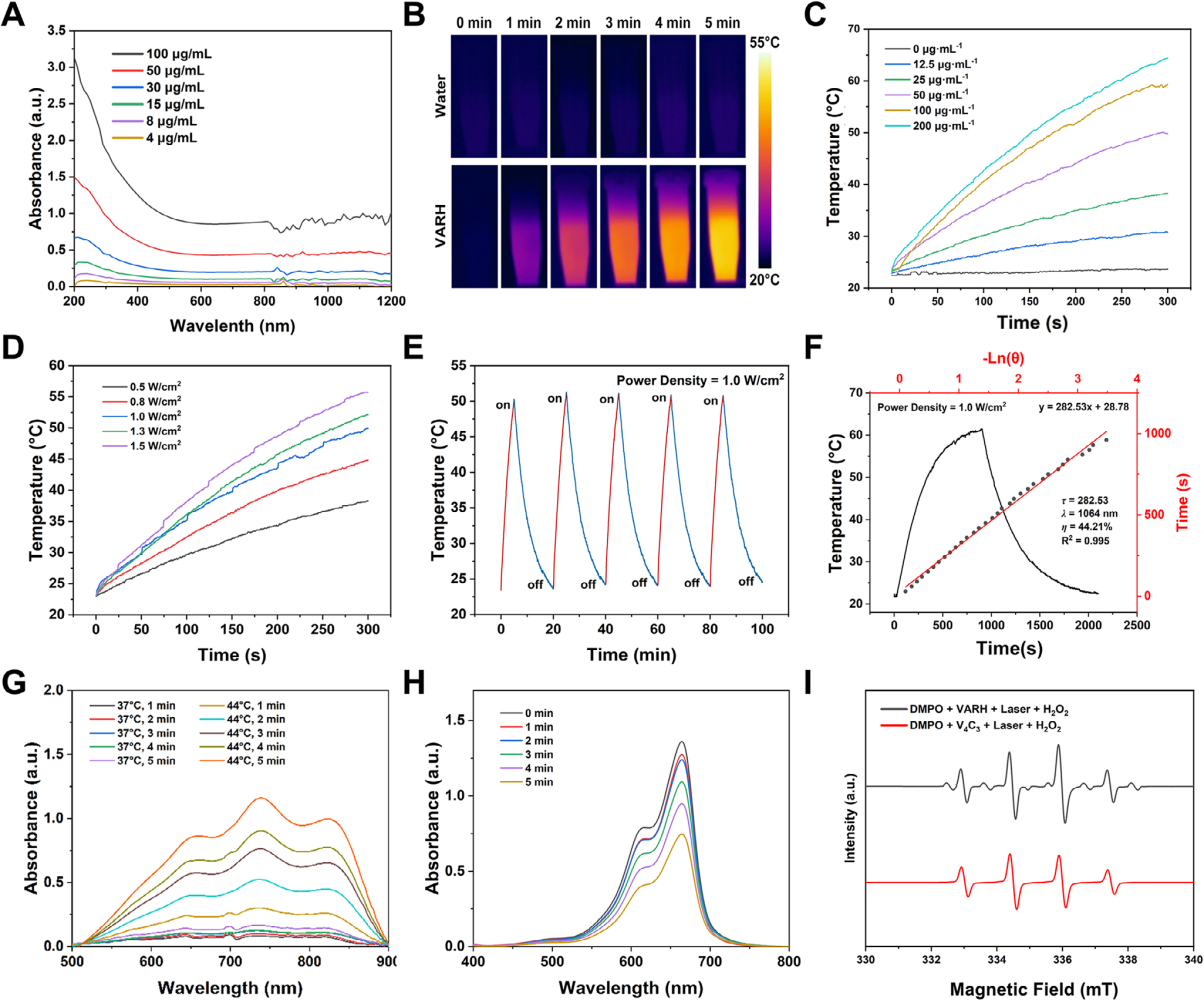
The photothermal properties and free radical release of VARH. (A) UV‐Vis‐NIR absorption spectrum of VARH at different concentrations. (B) Temperature variation of ultra‐pure water and VARH after irradiation with 1064 nm laser. (C) Temperature variation of VARH at different concentrations after irradiation with 1064 nm laser (1.0 W/cm^2^). (D) Temperature variation of VARH (V_4_C_3_ concentration at 50 µg/mL) after irradiation with 1064 nm laser at different power densities. (E) Photothermal stability of VARH after 5 “heating‐cooling” cycles. (F) Calculation of photothermal conversion efficiency of VARH. (G) UV–vis absorption spectrum of VARH after 5 min of heating in a water bath at 37°C and 44°C (reflect the alkyl radical). (H) UV–vis absorption spectrum of VARH After irradiation with laser for a certain duration (reflect the hydroxyl radical). (I) ESR spectra of V_4_C_3_ and VARH after laser irradiation.

VARH retained the strong light absorption capability of V_4_C_3_ in the NIR‐II window at 1064 nm, which exhibited a positive correlation with concentration, indicating excellent photothermal conversion potential (Figure 2A). Compared with ultrapure water, VARH showed a significant temperature increase under laser irradiation (Figure 2B). At a power density of 1.0 W/cm^2^, the temperature of a 200 µg/mL VARH solution rose to approximately 65°C after 5 min of irradiation (Figure 2C); at a fixed concentration of 50 µg/mL, the temperature reached 48.8°C under the same irradiation conditions (Figure 2D). After five laser on‐off cycles, no attenuation in the heating performance was observed, demonstrating excellent photothermal stability (Figure 2E). According to the calculation formula [41, 42], the photothermal conversion efficiency of VARH was determined to be 44.21% (Figure 2F), indicating that VARH possesses favorable properties for photothermal therapy of tumors.

The generation of ∙R by AAPH at different temperatures was assessed by monitoring the characteristic absorption of ABTS^+^·. As shown in Figure 2G, VARH released a considerable amount of ∙R when incubated in a 44°C water bath, whereas almost no release was detected at 37°C, indicating the temperature‐sensitive decomposition of AAPH, which occurs only at elevated temperatures. Upon irradiation with a 1064 nm laser, the release of ∙R from VARH was time‐dependent, confirming that photothermal heating effectively triggers AAPH decomposition (Figure S10).

MB is an aromatic heterocyclic compound that appears as a dark blue cation in solution. When ·OH reacts with MB in the system, it abstracts hydrogen atoms or disrupts the structure of MB, oxidizing it to a colorless radical cation [43, 44, 45]. In the absence of light, VARH caused a slight decrease in the UV absorption of MB due to ·OH generated from trace V^4+^ (Figure S11). After 5 min of irradiation, the temperature increase promoted substantial generation of V^4+^, accelerating the Fenton‐like reaction and leading to a marked increase in ·OH production. This resulted in rapid oxidation of MB and a significant decrease in absorbance (Figure 2H).

ESR spectroscopy was further employed to detect the types of free radicals generated by V_4_C_3_ and VARH after laser irradiation. As shown in Figure 2I, after 1064 nm laser irradiation, V_4_C_3_ exhibited a characteristic four‐line signal (peak intensity ratio 1:2:2:1) specific to ·OH. In contrast, VARH loaded with AAPH, upon thermal activation, produced ·R in addition to ·OH. The ESR spectrum of VARH displayed not only the four‐line signal of ·OH but also a distinctive six‐line signal (peak intensity ratio 1:1:1:1:1:1) characteristic of ·R (Figure S12). These results demonstrate that under laser excitation, VARH effectively generates both ·OH and ·R, indicating its potential for CDT and TDT in tumor cells.

### 3.3 In Vitro Uptake and Gene Expression of VARH

**FIGURE 3:**
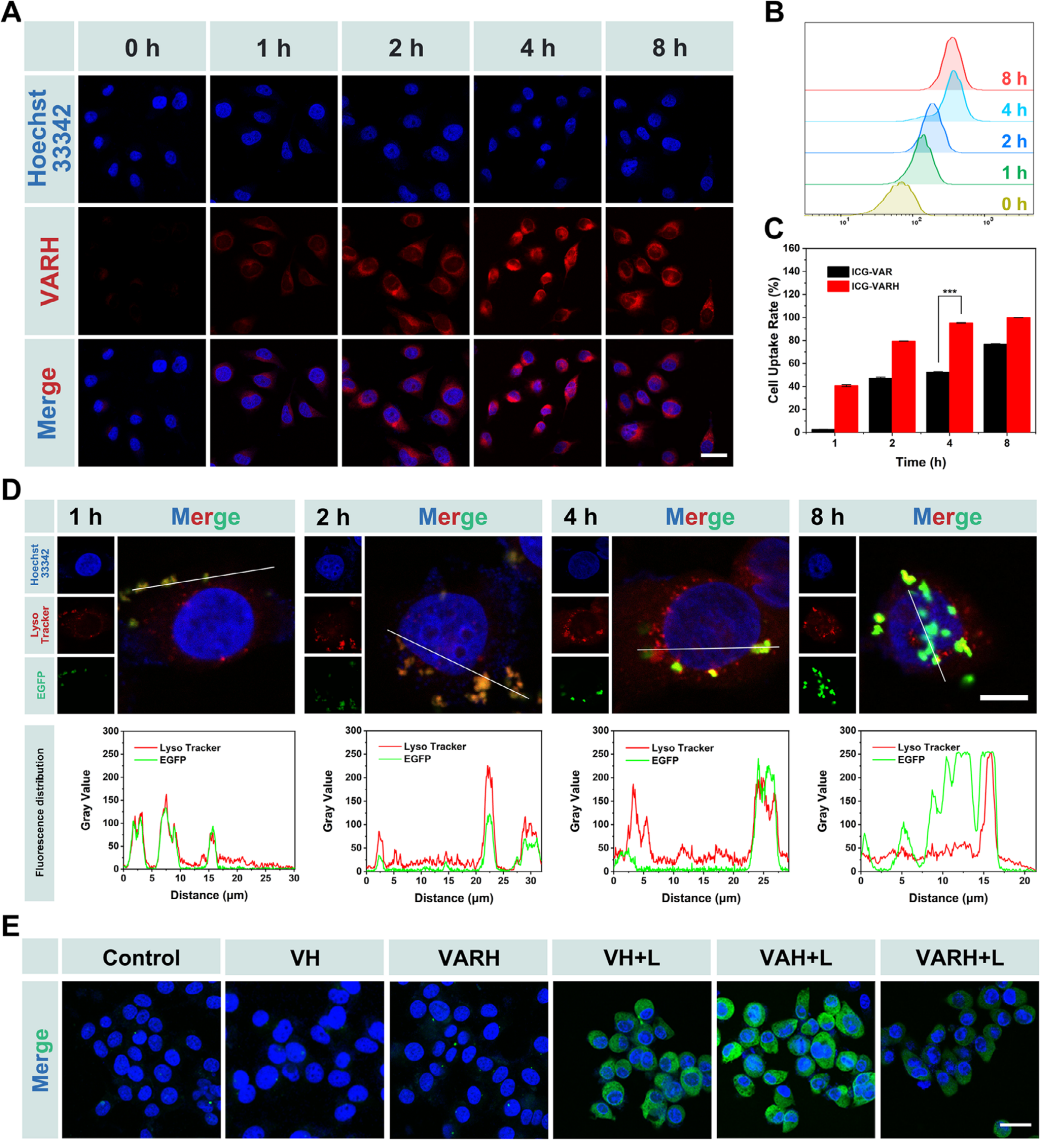
In vitro uptake and gene expression of VARH. (A) CLSM images of HepG2 cells at different time points after uptake of VARH, scale bar = 30 µm. (B) FCM results of HepG2 cells at different time points after uptake of VARH. (C) Uptake rate analysis of HepG2 cells for VAR and VARH nanocomposites. (D) Lysosomal escape and colocalization analysis of VARH nanocomposites, scale bar = 10 µm. (E) Immunofluorescence images of HSP90 protein in HepG2 cells after treatment with different formulations, scale bar = 30 µm.

To evaluate the targeting capability of HA modification, VARH was labeled with ICG, and its uptake by HepG2 cells was assessed using CLSM and FCM. CLSM results (Figure 3A) revealed a gradual increase in the cellular uptake of VARH from 0 to 4 h, peaking at 4 h and slightly decreasing by 8 h. Quantitative FCM analysis further indicated (Figure 3B and Figure S13) that cellular uptake approached saturation by 4 h, with no significant difference in fluorescence intensity observed between 4 and 8 h. In the free HA competition uptake experiments (Figure S14), the uptake efficiency by cancer cells pre‐incubated with free HA was significantly reduced. These results indicate that VARH can enhance cellular uptake by utilizing the CD44 receptor on cancer cells.

Using VAR without HA modification as a control, VARH exhibited higher uptake at all time points in CD44‑overexpressing HepG2 cells (Figure 3C), reaching 95.3% at 4 h. In contrast, the uptake rate in the VAR group reached only 76.8% by 8 h. These results demonstrate that HA modification not only enhances the targeted uptake of the nanocomplex but also promotes CD44‑mediated endocytosis.

The antitumor efficacy of VARH relies critically on lysosomal escape and nuclear entry of the ribonucleoprotein (RNP). As shown in Figure 3D, VARH was internalized and colocalized with lysosomes within 1 h. At 2 h, complete overlap was observed between Cas9 (green) and lysosomal (red) fluorescence. After 4 h, colocalization decreased, suggesting lysosomal disruption, likely attributable to the exposure of positively charged VAR following HA degradation by hyaluronidase (HAase), which may facilitate disruption via the proton sponge effect [46, 47]. By 8 h of incubation, RNP was released in response to GSH and translocated into the nucleus under the guidance of its nuclear localization sequence (NLS) to exert gene editing activity.

Analysis of GSH levels in the different treatment groups (Figure S15) revealed that VARH+L significantly downregulated intracellular GSH content through GSH‐responsive release and radical generation, thereby enhancing the therapeutic efficacy against tumors. To evaluate the gene editing capability of the RNP complex, HSP90 protein expression after heat stimulation was first examined via immunofluorescence (Figure S16). As shown in Figure 3E, weak fluorescence signals were detected in non‑irradiated cells. Upon irradiation, elevated HSP90 expression was observed in the VH+L, VAH+L, and VARH+L groups. However, in the VARH+L group, RNP‑mediated gene editing downregulated HSP90 expression, resulting in lower fluorescence intensity compared with other irradiated groups. Quantitative analysis further revealed (Figure S17) that HSP90 expression in the VH and VARH groups showed no significant difference from the Control group, while expression in the VH+L and VAH+L groups was upregulated to 5.47‑ and 6.70‑fold that of the Control, respectively, potentially due to alkyl radical‑induced oxidative stress promoting HSP90 expression [48]. In the VARH+L group, HSP90 expression was 3.33‑fold that of the Control, significantly lower than in other irradiated groups. The T7EI cleavage assay (Figure S18) showed cleavage bands only in the VAR and VARH groups loaded with RNP, indicating successful editing of the target gene. Compared with the VAR group, VARH exhibited enhanced tumor accumulation due to HA modification, achieving a gene editing efficiency of 27.3%. In the non‐targeting sgRNA control group and the HSP90 inhibitor 17‐AAG group (Figures S19 and S20), the expression level of HSP90 was significantly reduced in the VARH+L treatment group, demonstrating the reliable gene knock‐out efficacy of the Mxene‐based CRISPR/Cas9 system. These results confirm that the nanocomplex can effectively edit the HSP90 gene, demonstrating considerable potential for gene therapy.

### 3.4 Cytotoxicity of VARH

**FIGURE 4:**
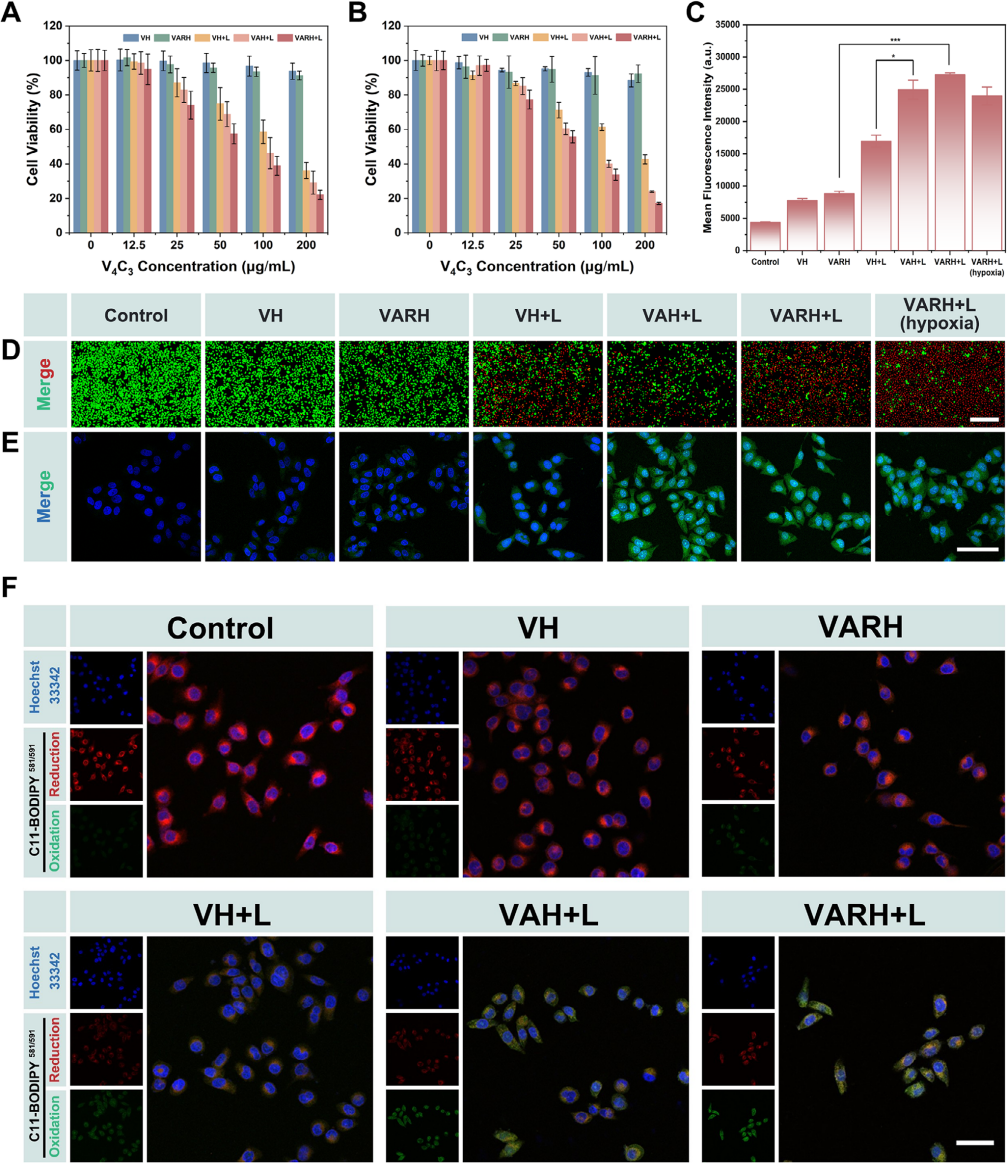
The cytotoxic effect of VARH. Cytotoxicity of different nanodrug formulations against (A) HepG2 cells and (B) H22 cells. (C) Fluorescence quantification of free radical generation in different administration groups. (D) Live/dead cell staining images of HepG2 cells treated with different nanodrug formulations, scale bar = 200 µm. (E) CLSM images of free radical generation in different administration groups, Scale bar = 100 µm. (F) CLSM images of lipid peroxidation in HepG2 cells induced by different treatment groups, scale bar = 50 µm.

In the cytotoxicity assays conducted on HUVEC, L929, and NIH‐3T3 cells (Figure S21), the VARH nanocomplex itself did not exhibit significant cytotoxicity. The effects of different nanoformulations and illumination conditions on the viability of HepG2 cells are shown in Figure 4A. At a V_4_C_3_ concentration of 50 µg/mL, the survival rate of the VH group decreased from 98.5% to 74.8% after irradiation with a 1064 nm laser, indicating that the photothermal effect alone exerted a certain cytotoxic effect. When the nanosystem was loaded with AAPH to generate ·R, the combination of photothermal therapy and free radical treatment (VAH+L group) further reduced the cell survival rate to 68.8%. Moreover, the incorporation of the RNP complex to mitigate thermal resistance and enhance photothermal efficacy (VARH+L group) resulted in a further decrease in the survival rate to 57.5%.

A similar trend was observed in H22 cells (Figure 4B). Under the condition of 200 µg/mL V_4_C_3_, the cell survival rate in the VARH+L group was only 17.1%. Overall analysis indicated that the cytotoxicity in the laser‐irradiated groups was concentration‐dependent, whereas no significant changes were observed in the non‐irradiated groups, suggesting that this strategy possesses favorable biosafety and controllable cytotoxicity. These results demonstrate that the strategy combining photothermally triggered free radicals with gene editing to amplify photothermal killing effectively inhibits tumor cell growth.

To further investigate the cytotoxicity of VARH triggered by the 1064 nm laser, Calcein AM/PI staining was employed to visually assess tumor cell killing (Figure S22). As shown in Figure 4D, without laser irradiation, the VH and VARH groups showed no significant killing effect on HepG2 cells, with green fluorescence intensity similar to that of the Control group. After laser irradiation, the combined action of photothermal effects and free radicals led to a gradual increase in red fluorescence and a rise in the number of dead cells, demonstrating significant toxicity. Under hypoxic conditions, the cytotoxic capacity of ·R was not markedly attenuated, indicating that these radicals can effectively overcome the tumor hypoxic microenvironment upon photothermal activation, thereby achieving efficient tumor suppression.

The generation of ·OH and ·R was detected using the DCFH‐DA probe (Figure S23). As shown in Figure 4C,E, only weak free radical signals were detected when VH or VARH was used alone. After laser irradiation, the VH group exhibited significantly enhanced fluorescence due to intracellular catalysis of H_2_O_2_ to generate ·OH, a reaction accelerated under elevated temperature. In the VAH+L and VARH+L groups loaded with AAPH, further increase in fluorescence intensity was observed due to the thermal decomposition of AAPH producing ·R. In an artificially constructed tumor hypoxia model, free radical generation remained considerable, with fluorescence intensity close to that of the VAH+L group, indicating that a high concentration of free radicals could be maintained within the cells.

Lipid peroxides (LPO) are products formed by the oxidation of lipid molecules via free radicals or enzymatic catalysis [49]. Changes in LPO levels in HepG2 cells across different treatment groups were detected using the C11‐BODIPY probe. As shown in Figure 4F, LPO levels in the VH and VARH groups showed no significant difference compared to the Control group. However, after laser irradiation (VH+L group), the reduced red fluorescence decreased while the oxidized green fluorescence increased, indicating that ·OH promoted LPO generation. Following the introduction of AAPH to generate ·R, LPO accumulation was further enhanced, demonstrating that excessive free radicals can induce lipid peroxidation, thereby disrupting normal cellular physiological activities.

### 3.5 Assessment of Mitochondrial Membrane Potential and DAMP Release

**FIGURE 5:**
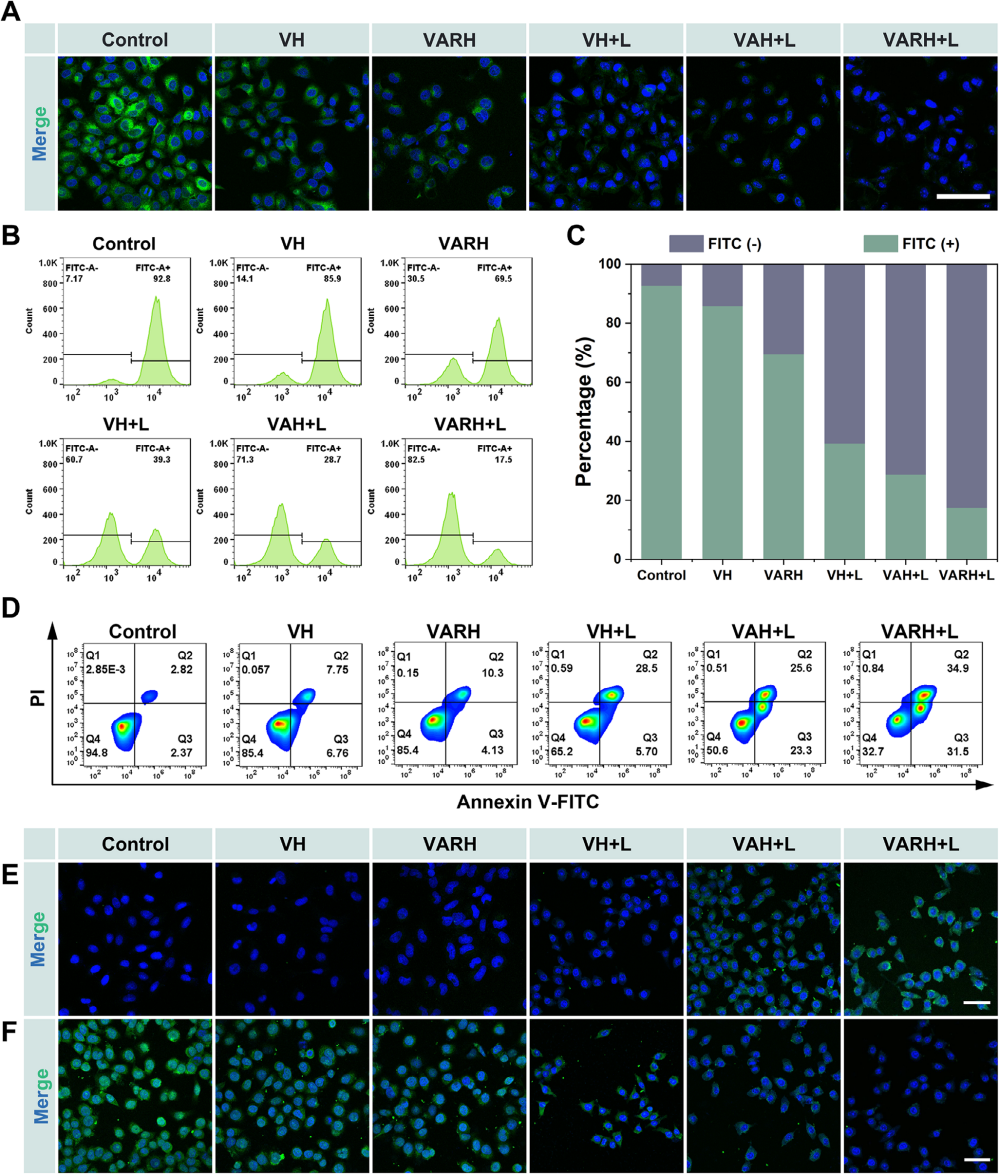
Mitochondrial changes and DAMP release. (A) CLSM images of mitochondrial membrane potential changes in HepG2 cells induced by different treatment groups, scale bar = 100 µm. (B,C) FCM quantitative analysis of mitochondrial membrane potential changes in HepG2 cells induced by different treatment groups. (D) FCM quantitative analysis of apoptosis in HepG2 cells after treatment with different formulations. (E) CRT exposure in HepG2 cells after different treatment, scale bar = 50 µm. (F) Release of HMGB1 in HepG2 cells after different treatment, scale bar = 50 µm.

Photothermal stimulation, aberrant elevation of radical concentrations, and accumulation of lipid peroxides can induce mitochondrial dysfunction in tumor cells [50], subsequently leading to a decrease in mitochondrial membrane potential (ΔΨ_m_). The ΔΨ_m_ reflects the functional status of mitochondria, and its alteration is closely associated with apoptosis. Changes in ΔΨm were detected using the Rhodamine 123 probe (Figure S24). The results in Figure 5A show that compared to the Control group, the green fluorescence intensity slightly decreased in the VH and VARH groups, but the mitochondria in most cells remained in a normal polarized state. After irradiation with a 1064 nm laser, significant fluorescence attenuation was observed in the VH+L, VAH+L, and VARH+L groups, with the green fluorescence almost completely disappearing in the VARH+L group, indicating that the combined action of photothermal effects and free radicals severely disrupted mitochondrial function. Further quantitative analysis by FCM (Figure 5B,C) revealed that the proportion of cells with altered ΔΨ_m_ was 14.1% in the VH group, which increased to 60.7% after laser irradiation. In the VARH group, due to its inherent slow Fenton‐like reaction and decomposition of AAPH generating a small amount of radicals, this proportion increased from 7.17% in the Control group to 30.5%. Under the combined action of photothermal effects, the Fenton‐like reaction, and a large amount of ·R, the proportion in the VARH+L group reached as high as 82.5%. These results demonstrate that photothermal effects and free radicals can synergistically damage the mitochondrial membrane structure, promoting the release of pro‐apoptotic factors into the cytoplasm, thereby inducing mitochondria‐mediated apoptosis.

Based on the detected changes in ΔΨ_m,_ cell apoptosis was further evaluated by Annexin V/PI staining. The results in Figure 5D show that compared to the Control group, the apoptosis rate slightly increased in the VH and VARH groups, a trend consistent with the ΔΨ_m_ changes. The apoptosis rate in the VARH+L group reached 66.4%. Furthermore, compared to the VH+L group, it was observed that ∙R significantly induced early apoptosis. After knocking down HSP90 expression using RNP complexes to enhance thermal sensitivity, the early apoptosis rate further increased from 23.3% to 31.5%, indicating that the combination of photothermal therapy and free radicals effectively promotes cell apoptosis.

Related studies have shown that after tumor cells undergo apoptosis induced by PTT, CDT, or TDT treatments, a series of cytological responses are initiated through the release of DAMPs, such as CRT exposure and HMGB1 secretion, thereby inducing ICD. The extent of ICD can be assessed by detecting CRT and HMGB1 using immunofluorescence (Figures S25–S28).

The results in Figure 5E show that the fluorescence intensity was weakest in the Control, VH, and VARH groups. Weak fluorescence was observed in the VH+L group, while the fluorescence intensity was markedly increased in the VAH+L and VARH+L groups, with fluorescence primarily distributed on the cell membrane. This indicates that VARH+L treatment successfully induced ICD, promoting CRT exposure on the cell surface. The results of HMGB1 release detection (Figure 5F) show that compared to the Control group, the fluorescence intensity began to decrease in the VH and VARH groups. After laser irradiation, the content of HMGB1 released extracellularly by tumor cells increased, accompanied by a significant decrease in intracellular fluorescence intensity. These results suggest that the combined action of thermal effects and free radicals can effectively induce ICD.

### 3.6 Transcriptomic Analysis of VARH Laser Treatment

**FIGURE 6:**
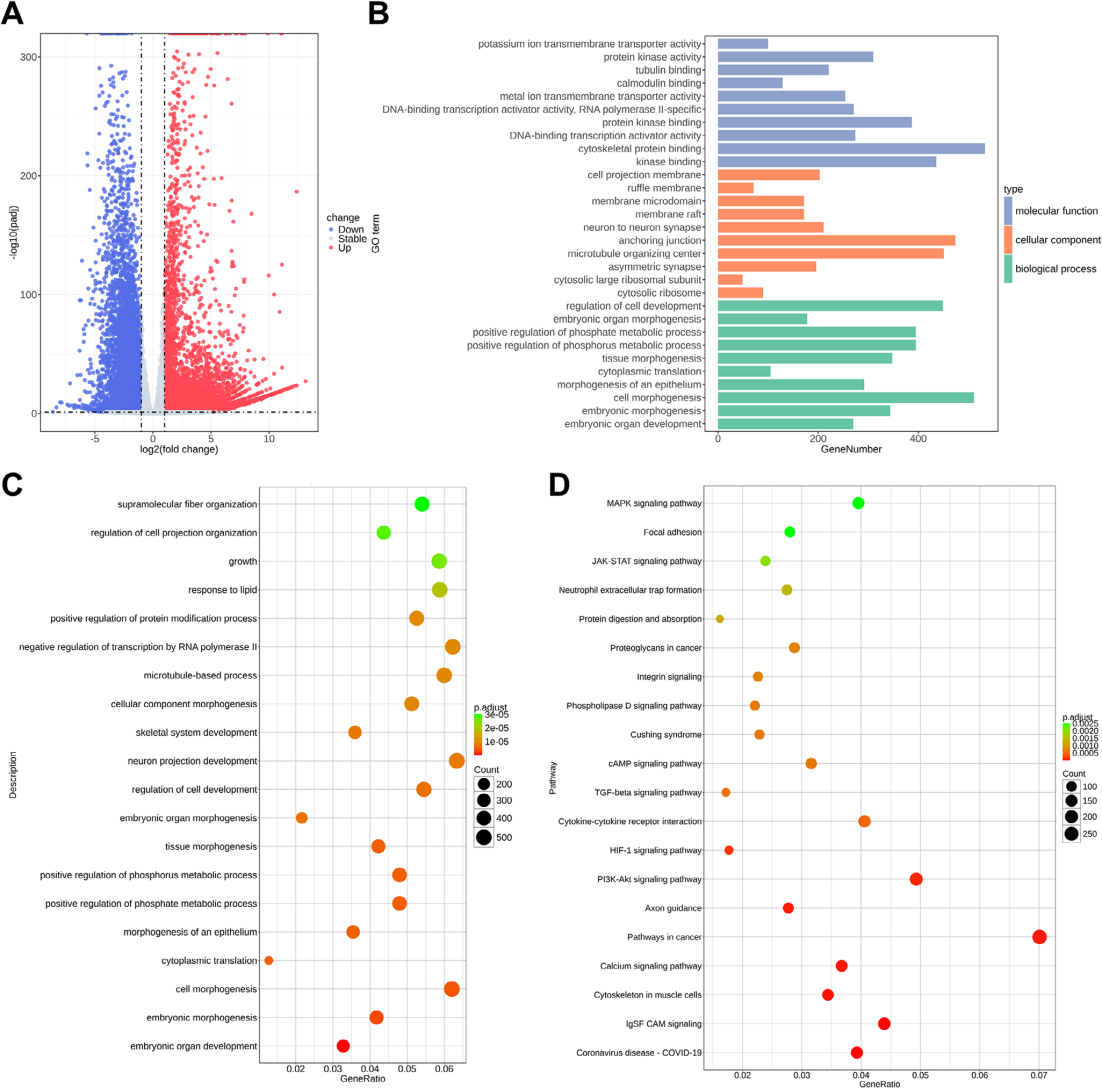
Transcriptomic analysis of VARH laser treatment. (A) Volcano plot showing the differentially expressed genes identified from the comparison of VARH+L versus Control. (B) GO enrichment plot showing categorized results from the comparative analysis of VARH+L versus Control. (C) Top 20 Enriched GO Terms (Biological Process) of VARH+L versus Control. (D) Bubble plot of KEGG pathway enrichment of VARH+L versus Control.

To investigate the molecular mechanism underlying VARH‐induced HepG2 cell death, RNA‐seq transcriptomic analysis was performed to identify differentially expressed genes (DEGs) between the Control group and the VARH+L group. The sample correlation heatmap revealed highly consistent expression profiles among biological replicates within each group, while inter‐group correlations were significantly reduced, indicating substantial transcriptomic reprogramming induced by VARH+L treatment (Figure S29). Volcano plot analysis visualized 15 298 genes with altered expression in the VARH+L group compared to the Control group, comprising 10 288 upregulated and 5010 downregulated genes (Figure 6A and Figure S30). The DEG clustering heatmap further confirmed that samples from the two groups formed distinct branches with high intra‐group reproducibility and significant inter‐group differences. The DEGs exhibited clear co‐expression patterns, with some genes highly expressed in the control group and lowly expressed in the treatment group, and vice versa (Figure S31). These findings demonstrate that VARH+L treatment significantly modulates gene expression.

To elucidate the biological significance of the DEGs, Gene Ontology (GO) and Kyoto Encyclopedia of Genes and Genomes (KEGG) enrichment analyses were conducted. GO analysis revealed significant enrichment of DEGs in biological processes (BP), cellular components (CC), and molecular functions (MF) (Figure 6B,C and Figures S32–S33). The results suggest that VARH may block the core life activities of tumor cells in multiple dimensions by influencing cytoskeletal protein binding, kinase binding, anchoring junction, microtubule organizing center, cell morphogenesis, regulation of cell development and other pathways, thereby achieving anti‐tumor effects.

Further analysis of the top 30 most significant GO terms (Figure S34) indicated that in BP, VARH primarily suppressed genes related to “tumor proliferation/metabolism”; in CC, it mainly inhibited genes associated with “pro‐tumor structures”; and in MF, it predominantly downregulated genes involved in “pro‐tumor signaling/transcription”. This aligns with the notion that VARH directly damages cellular structures via PTT/free radical effects and interferes with kinase/transcription factor functions through HSP90 gene editing. The GO gene‐pathway association network further supported a “modular regulatory” role for VARH, suggesting its ability to coordinately target core gene modules at the molecular, cellular, and physiological levels in tumors (Figures S35–S37).

KEGG enrichment analysis demonstrated significant alterations in pathways including calcium signaling, cytoskeleton‐related pathways, extracellular signal transduction, and certain cancer pathways (Figure 6D and Figure S38). These findings are consistent with the proposed mechanism wherein VARH inhibits pro‐tumor signaling via HSP90 gene editing while simultaneously disrupting the cytoskeleton through photothermal/free radical effects, ultimately achieving multi‐dimensional anti‐tumor efficacy by regulating a coordinated “pathway‐gene” network. Collectively, these results systematically untangle the key gene functions and signaling pathways modulated by VARH treatment.

### 3.7 In Vivo Antitumor Efficacy of VARH

To evaluate the biocompatibility of the VARH nanocomposite, hemolysis assay results (Figure S39) showed that no erythrocyte rupture was observed when the concentration of V_4_C_3_ in VARH reached 200 µg/mL, and the hemolysis rate was less than 5%. Furthermore, a comprehensive blood safety analysis (Figure S40) was conducted to examine indicators such as white blood cells, red blood cells, and platelets in mice from each treatment group after administration. The results indicated no significant abnormalities in any group, demonstrating that VARH exhibits favorable biocompatibility.

**FIGURE 7:**
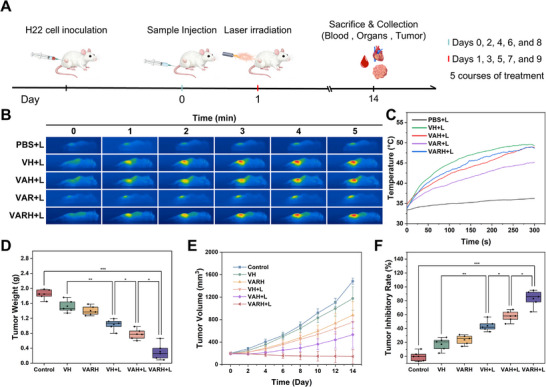
In vivo anti‐tumor efficacy of VARH. (A) Schematic illustration of in vivo treatment. (B) Infrared thermal imaging of H22 tumor‐bearing mice 24 h after tail vein injection of different drugs under laser irradiation, and (C) Temperature change curves at the tumor site within 0–5 min. (D) Tumor weight of mice in different groups after treatment. (E) Tumor volume changes in H22 tumor‐bearing mice during treatment. (F) Tumor inhibition rate in mice treated with different formulations.

The in vivo antitumor efficacy of VARH combined with laser irradiation was assessed in H22 tumor‐bearing mice. Experimental groups included Control, VH, VARH, VH+L, VAH+L, and VARH+L. Administration was performed every 2 days, and laser irradiation was conducted 24 h post‐administration. This cycle was repeated five times, and the mice were euthanized and samples were collected on day 14 (Figure 7A).

First, the in vivo distribution of VAR and VARH was evaluated. VAR and VARH were labeled with ICG to monitor their distribution in tumor‐bearing mice from 2 to 24 h (Figure S41). The results showed that the agents gradually accumulated in the tumor tissue via blood circulation, reaching peak concentrations at 4 and 8 h, respectively. Compared to VAR, which relies solely on the EPR effect for tumor enrichment, VARH, leveraging the active targeting capability of HA, achieved higher and more sustained accumulation, maintaining a relatively high concentration even at 24 h. This is conducive to its gene editing function, subsequently downregulating HSP90 expression and enhancing the subsequent photothermal therapeutic effect. Distribution analysis of ex vivo fluorescence in tumors and major organs showed significantly lower hepatic uptake and higher tumor accumulation of VARH, indicating that HA modification enhances tumor targeting.

Given the excellent photothermal conversion performance of VARH ex vivo and its favorable targeting ability in vivo, its in vivo photothermal effect was further evaluated. 24 h after tail vein injection of the agents, the tumor region was irradiated with a 1064 nm laser for 5 min. The results in Figure 7B,C show that the tumor temperature in the Control group did not increase significantly, whereas the nanoagent groups containing V_4_C_3_ exhibited varying degrees of temperature elevation. The VAR+L group, lacking HA modification, showed limited temperature increase due to lower drug concentration at the tumor site. In contrast, the VH+L, VAH+L, and VARH+L groups all demonstrated good photothermal performance, with maximum temperatures reaching 49.6°C, 49.0°C, and 48.2°C, respectively, effectively achieving tumor photothermal ablation and inhibiting tumor progression.

The tumor inhibition results (Figure 7D–F) showed rapid tumor growth in the control group. In the VH group, the Fenton‐like reaction of V_4_C_3_, and in the VARH group, the slow decomposition of AAPH, both generated a small amount of free radicals, which partially inhibited tumor growth. After laser irradiation, the VH+L group exhibited a significant therapeutic effect, attributable to the efficient photothermal conversion capability of V_4_C_3_ for tumor ablation and the temperature‐accelerated catalysis of H_2_O_2_ by V^4+^ to generate ·OH, enhancing tumor cell killing, with a tumor inhibition rate of 44.10%. In the VAH+L group loaded with AAPH, a substantial amount of ·R was additionally generated upon thermal triggering, further increasing the concentration of free radicals at the tumor site and resulting in a tumor inhibition rate of 57.96%. On this basis, the RNP complex enhanced the thermal sensitivity of tumor cells by knocking down HSP90 expression, thereby amplifying the therapeutic effect of photothermal therapy. This led to a tumor inhibition rate of 83.21% in the VARH+L group, demonstrating significant antitumor efficacy. Furthermore, the expression of the hypoxia marker HIF‐1α in tumor tissues from each group was detected via immunohistochemical assays (Figure S42). It was found that the HIF‐1α‐positive results were comparable across all groups, confirming the presence of a stable hypoxic microenvironment in the H22 tumor model. Combined with the therapeutic efficacy data, these findings further substantiate the advantages of TDT under hypoxic conditions.

Tumor tissue morphology was observed via H&E staining (Figure S43). In the Control group, the cells exhibited an intact morphology with densely packed nuclei, indicative of active proliferation. In all other groups, nuclear fragmentation or karyolysis was observed in the blue nuclear regions. Furthermore, cytoplasmic vacuolization was evident in the VARH+L group, suggesting severe apoptosis or necrosis of tumor cells.

### 3.8 Biosafety and In Vivo Immune Activation of VARH

No abnormal body weight loss was observed in mice from any treatment group (Figure S44), indicating favorable in vivo safety of the VARH nanomedicine. After treatment completion, liver and kidney function were assessed by measuring serum biochemical markers ALT, AST, and BUN [51]. Markedly elevated levels of these three indicators in the Control group suggested severe hepatic and renal injury, whereas all treatment groups exhibited varying degrees of reduction in these markers (Figure S45). H&E staining was performed on major organs (Figure S46). In the heart, liver, spleen, lungs, or kidneys of mice in the treatment group, no significant damage to cellular morphology, distribution, or structure was observed. The T7E1 digestion assay (Figure S47) indicated no detectable editing of the HSP90 gene across the five major organs. Concurrently, it was found in our previous studies that V_4_C_3_ MXene undergoes controlled in situ degradation only under the action of high concentrations of H_2_O_2_ or GSH, which are characteristic of the tumor microenvironment, and can be smoothly excreted from the body via the kidneys [52]. These findings demonstrate the favorable biosafety profile of the administered nanomedicine.

**FIGURE 8:**
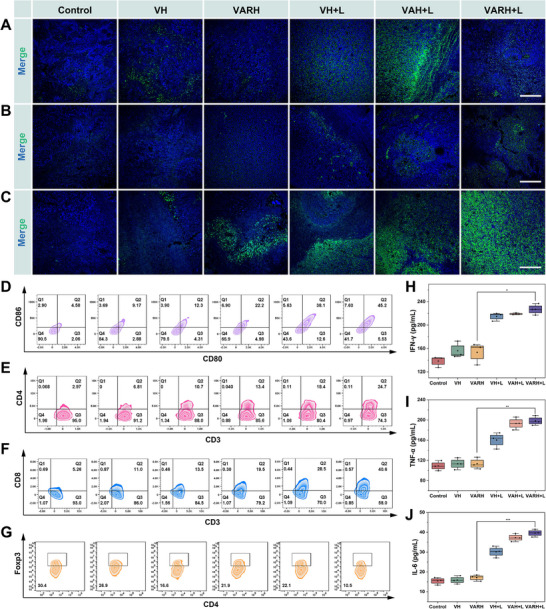
In vivo immune activation of VARH. (A) Immunofluorescence images of HSP90 protein in tumor tissues of tumor‐bearing mice after treatment with different therapeutic groups, Scale bar = 200 µm. (B) CRT exposure in tumor tissues of tumor‐bearing mice after treatment with different groups, scale bar = 200 me. (C) HMGB1 release in tumor tissues of tumor‐bearing mice after treatment with different groups, scale bar = 200 µm. Changes in the cell population proportion of (D) DC cells, (E) CD4^+^ T cells, (F) CD8^+^ T cells, and (G) Treg cells in tumor tissues of tumor‐bearing mice across different groups. Changes in the levels of immune‐related factors in the serum of tumor‐bearing mice from each group: (H) IFN‐γ, (I) TNF‐α, and (J) IL‐6.

After confirming the robust gene editing capability of VARH ex vivo, its inhibitory effect on HSP90 protein expression in vivo was further evaluated via immunofluorescence (Figure S48). Figure 8A shows a low baseline level of HSP90 expression in the non‐irradiated group. Fluorescence intensity was significantly enhanced in the VH+L and VAH+L groups, with the VAH+L group exhibiting the most pronounced increase, consistent with findings from ex vivo experiments. In contrast, HSP90 expression was significantly reduced in the VARH+L group, with fluorescence intensity comparable to that of the non‐irradiated group. Quantitative analysis further confirmed this trend (Figure S49). The expression levels in the VH and VARH groups were 1.00‐ and 1.50‐fold that of the Control group, respectively. Upon laser irradiation, levels in the VH+L and VAH+L groups increased to 2.05‐ and 2.10‐fold, respectively. However, following HSP90 gene knockout, the expression level in the VARH+L group was measured at only 1.11‐fold relative to the Control, a value markedly lower than that of other irradiated groups. This substantial reduction indicates a significant inhibitory effect on the target protein.

To investigate the mechanism by which VARH induces ICD in vivo, its effect on the release of DAMPs under laser irradiation was first validated in vivo, including CRT exposure (Figure S50) and HMGB1 release (Figure S51). As shown in Figure 8B, minimal green fluorescence was observed in the Control, VH, and VARH groups, whereas fluorescence intensity was enhanced in the VH+L, VAH+L, and VARH+L groups, indicating increased CRT exposure. Results for HMGB1 release (Figure 8C) showed an increase in fluorescent area in the VH and VARH groups compared to the Control group. After laser irradiation, HMGB1 release from tumor cells increased in the VH+L and VARH+L groups, with a significant enhancement in intercellular fluorescence intensity. These results indicate that photothermal effects and free radical generation are key factors promoting the release of CRT and HMGB1.

The activation status of the tumor immune microenvironment (TIME) was assessed by analyzing the proportions of immune cell populations. Previous immunofluorescence experiments demonstrated that VARH, upon irradiation, effectively induces CRT exposure, an “eat me” signal that enhances DC phagocytosis and recognition, leading to their subsequent maturation [53]. Figure 8D shows that the proportion of mature DCs increased from 9.17% to 22.2% in the VH group after irradiation, while in the VARH group, it increased from 12.3% to 45.2% post‐irradiation, indicating that VARH combined with photothermal therapy effectively induces ICD and promotes DC maturation via DAMP release.

Mature DCs enhance antigen‐presenting capacity, subsequently activating CD4^+^ T cells and CD8^+^ T cells. Results in Figure 8E,F show that the proportions of CD4^+^ T cells (24.7%) and CD8^+^ T cells (40.6%) in the VARH+L group were 8.32‐ and 7.72‐fold higher than those in the Control group, respectively, indicating that this treatment significantly promotes T cell infiltration and enhances their direct tumor‐killing effects.

Tregs possess immunosuppressive functions within the TIME, and it has been reported that HSP90 inhibition can lead to their reduction [54]. Figure 8G shows that compared to the Control group (30.4%), the proportions of Tregs were decreased in the VH, VARH, VH+L, VAH+L, and VARH+L groups, with values of 26.9%, 21.9%, 22.1%, 16.6%, and 10.5%, respectively. The decrease in Treg proportion indicates a weakened immunosuppressive function, thus relieving CD8^+^ T cells from suppression and leading to a more robust anti‐tumor immunity.

These soluble factors, including IFN‐γ, TNF‐α, and IL‐6, act locally against tumors and are capable of disseminating into the bloodstream to trigger body‐wide immune activation [55]. Figure 8H–J shows that the VARH+L group exhibited significantly higher levels of IFN‐γ, TNF‐α, and IL‐6 compared to all other groups, and the cytokine levels in all irradiated groups were markedly higher than those in non‐irradiated groups. These cytokines are primarily secreted by activated T cells and mature DCs. Elevated levels of secreted pro‐inflammatory cytokines potentiate the cytotoxic function of T cells, resulting in the elimination of tumor cells, indicating that the combination of PTT with CDT and TDT effectively stimulates a pro‐inflammatory response and activates the TIME.

## 4 Conclusion

In summary, this study successfully constructed a multifunctional nanoplatform, VARH, based on V_4_C_3_ MXene. This platform enables GSH‐responsive drug release within the tumor microenvironment, synergistically integrating PTT, free radical‐induced therapy, and gene editing, thereby significantly enhancing the synergistic killing effect on cancer.

Leveraging the strong NIR‐II absorption capacity and excellent photothermal conversion efficiency of V_4_C_3_ MXene, the VARH system achieves deep tissue penetration and induces tumor ablation upon 1064 nm laser irradiation. The intrinsic V^4+^ catalyzes the conversion of endogenous H_2_O_2_ into ·OH via a Fenton‐like reaction. Moreover, under hypoxic conditions, it triggers the on‐demand release of ·R from the thermosensitive compound AAPH, initiating TDT. Concurrently, the released RNP specifically targets the HSP90 gene, inhibiting heat shock protein expression and effectively reversing thermal resistance commonly encountered in conventional PTT, thereby significantly improving photothermal therapeutic outcomes. Furthermore, under the triple‐combination therapy of PTT/CDT/TDT, the system promotes the release of DAMPs and induces ICD, subsequently triggering an immune response.

This study demonstrates the promising application of spatiotemporally controlled integration of photothermal agents with gene editing and free radical‐based therapies, providing a valuable paradigm for the development of next‐generation minimally invasive and precise antitumor platforms. However, challenges such as long‐term toxicological evaluation and clinical standardization remain to be addressed before clinical translation can be realized, representing critical hurdles for our future research.

## Conflicts of Interest

The authors declare no conflicts of interest.

## Supporting information




**Supporting File**: advs74780‐sup‐0001‐SuppMat.docx.

## Data Availability

The data that support the findings of this study are available from the corresponding author upon reasonable request.
