# Supplementary material for: CRISPR/Cas9‐Based Vanadium MXene‐Free Radical Spatiotemporally Controlled Nanoreactor for Photothermal‐Induced Multi‐Effect Synergistic Antitumor Therapy
Source: Adv Sci (Weinh). 2026 Mar 12;13(29):e22535. doi: 10.1002/advs.202522535 (PMC13205661; doi:10.1002/advs.202522535)
Supplement: Supplementary file 1 — Supporting File: advs74780‐sup‐0001‐SuppMat.docx. [file ADVS-13-e22535-s001.docx]

**Supporting Information**

**CRISPR/Cas9-Based Vanadium MXene-Free Radical Spatiotemporally Controlled Nanoreactor for Photothermal-Induced Multi-Effect Synergistic Antitumor Therapy**

Zi-Jian Huang^1^, Feng-Ming Li^1^, Yi-Fan Tu^1^, Ke-Ke Feng^1^, Cheng-Lei Li^1^, Shi-Cheng Tian^1^, Yong-Shan Hu^1^, Jing-Wei Shao^1*^, Zhen-Hua Liu^2*^

1. College of Chemistry, Fuzhou University, Fuzhou, Fujian 350108, China.

2. Department of Oncology, Shengli Clinical Medical College of Fujian Medical University, Fuzhou University Affiliated Provincial Hospital, Fuzhou, China.

* Correspondences: E-mail: shaojingwei@fzu.edu.cn (Jing-Wei Shao); liuzhenhua6909@163.com (Zhen-Hua Liu)

**Supplementary Table**

**Table S1.** Oligonucleotide sequence.

| Oligo Name | Sequence (5′ to 3′) |
| --- | --- |
| sgHSP90α (Human) | 5′-GATCAAAAGGAGCACGTCGT-3′ |
| HSP90-F | 5′-ATCACCAGAGGCAGATGTGT-3′ |
| HSP90-R | 5′-GAGTCCTCAGCAGGGGGATC-3′ |
| sgHSP90α (Mouse) | 5′-ATGGTCTGCCCGGACGGTGA-3′ |

**Table S2.** Optimization of the ratio between V_4_C_3_ and PAH.

| V_4_C_3_:PAH (w/w) | particle size（nm） | PDI | zeta potential（mV） |
| --- | --- | --- | --- |
| 1:0 | 146.9 ± 1.5 | 0.258 ± 0.003 | -25.93 ± 0.85 |
| 1:1 | 167.1 ± 1.6 | 0.210 ± 0.032 | 6.41 ± 2.53 |
| 1:2 | 168.3 ± 0.9 | 0.225 ± 0.005 | 30.67 ± 1.76 |
| 1:4 | 221.5 ± 4.9 | 0.400 ± 0.035 | 43.33 ± 0.21 |
| 1:8 | 303.5 ± 3.8 | 0.403 ± 0.019 | 51.77 ± 0.25 |

**Table S3.** The names and RRIDs of the cells used in the experiment.

| Cell name | HepG2 | H22 | HUVEC | L929 | NIH-3T3 |
| --- | --- | --- | --- | --- | --- |
| RRID | CVCL_0027 | CVCL_H613 | CVCL_2959 | CVCL_0462 | CVCL_0594 |

**Table S4.** mRNA-seq Data Filtering Summary.

| Sample | RawReads (M) | CleanReads (M%) | Adapter (M%) | LowQuality (M%) | N (M%) |
| --- | --- | --- | --- | --- | --- |
| Control-1 | 56.11 | 56.1(99.98) | 0.71(1.27) | 0.0(0.0) | 0.01(0.02) |
| Control-2 | 54.74 | 54.73(99.98) | 0.65(1.19) | 0.0(0.0) | 0.01(0.02) |
| Control-3 | 52.67 | 52.66(99.98) | 0.7(1.33) | 0.0(0.0) | 0.01(0.02) |
| VARH+L-1 | 52.48 | 52.47(99.98) | 1.05(2.0) | 0.0(0.0) | 0.01(0.02) |
| VARH+L-2 | 60.39 | 60.38(99.98) | 1.33(2.2) | 0.0(0.0) | 0.01(0.02) |
| VARH+L-3 | 49.09 | 49.08(99.98) | 0.81(1.65) | 0.0(0.0) | 0.01(0.02) |

**Table S5.** mRNA-seq Base Composition Statistics.

| Sample | Raw  Bases(G) | BF_Q20  (G%) | BF_Q30  (G%) | BF_GC(%) | Clean  Bases  (G) | AF_Q20  (G%) | AF_Q30  (G%) | AF_GC(%) |
| --- | --- | --- | --- | --- | --- | --- | --- | --- |
| Control-1 | 8.42 | 8.24  (97.93) | 8.11  (96.31) | 49.76 | 8.39 | 8.22  (97.94) | 8.08  (96.31) | 49.75 |
| Control-2 | 8.21 | 8.04  (97.89) | 7.9  (96.25) | 49.75 | 8.19 | 8.02  (97.9) | 7.88  (96.25) | 49.74 |
| Control-3 | 7.9 | 7.75  (98.08) | 7.63  (96.55) | 49.68 | 7.88 | 7.73  (98.08) | 7.61  (96.55) | 49.67 |
| VARH+L-1 | 7.87 | 7.73  (98.17) | 7.61  (96.7) | 49.86 | 7.83 | 7.69  (98.17) | 7.57  (96.7) | 49.84 |
| VARH+L-2 | 9.06 | 8.89  (98.12) | 8.75  (96.64) | 49.78 | 9.01 | 8.84  (98.12) | 8.71  (96.65) | 49.77 |
| VARH+L-3 | 7.36 | 7.22  (98.04) | 7.11  (96.5) | 49.18 | 7.33 | 7.19  (98.04) | 7.07  (96.5) | 49.17 |

**Supplementary Figure**


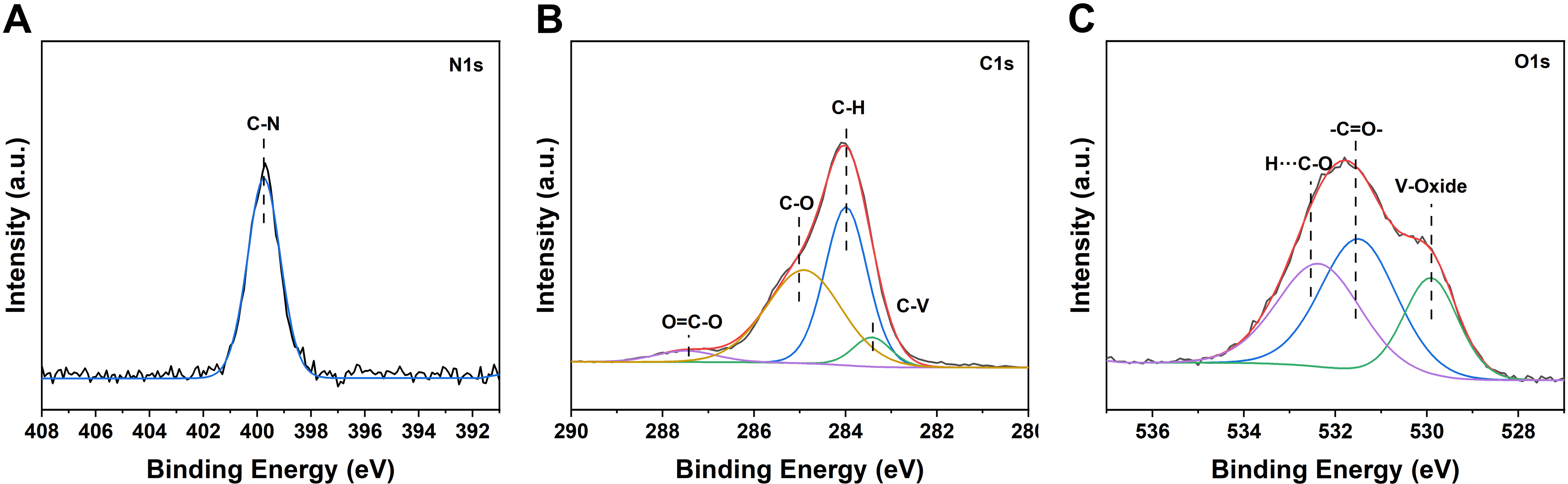


**Figure S1.** (A) Nitrogen element XPS spectrum of V_4_C_3_, (B) Carbon element XPS spectrum of VARH, (C) Oxygen element XPS spectrum of VARH.


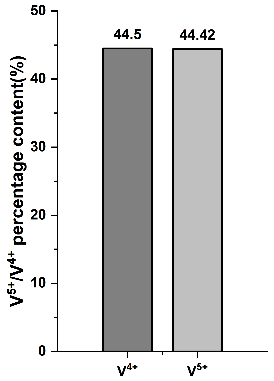


**Figure S2.** The content proportion of V^4+^/V^5+^ in VARH..


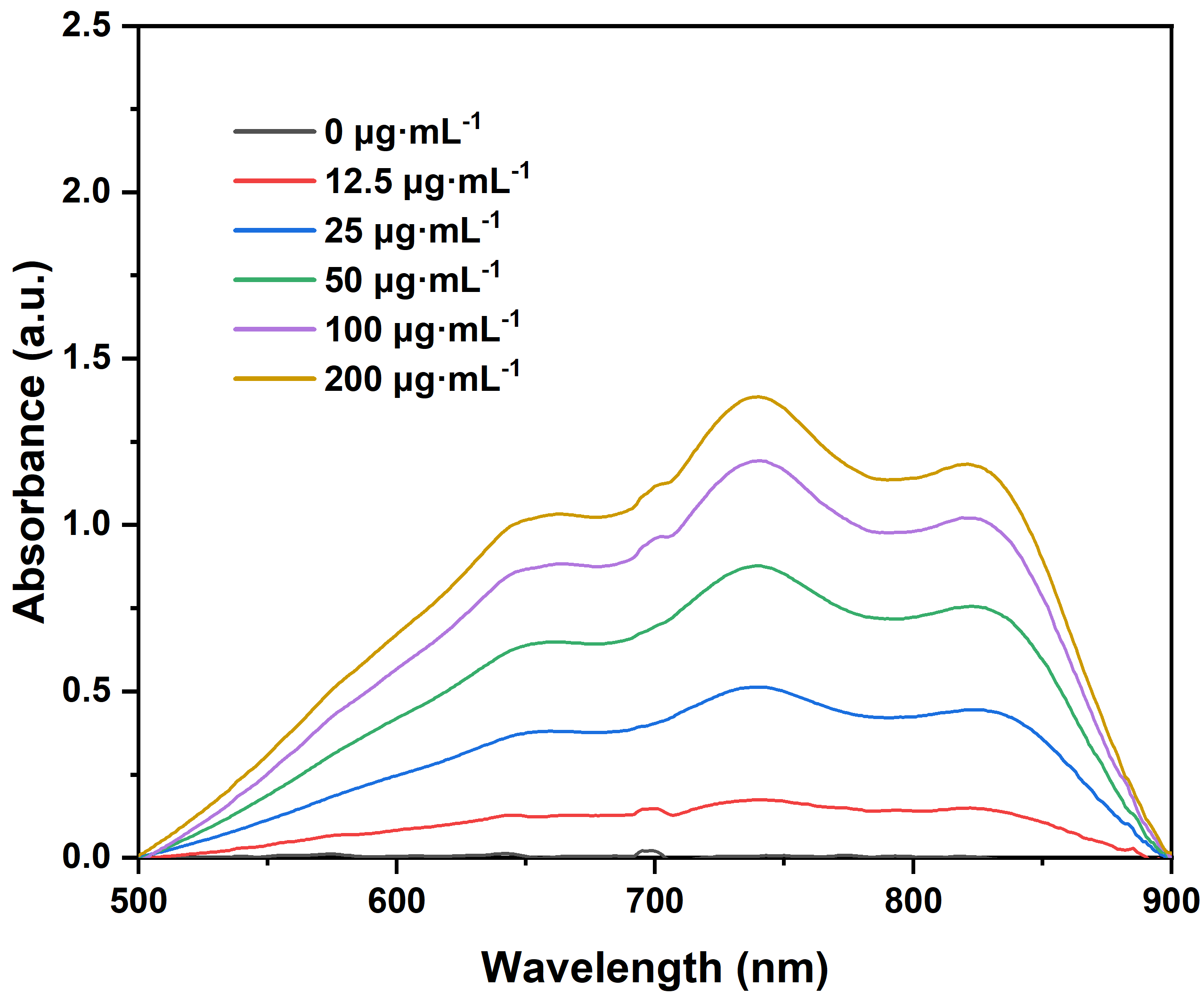


**Figure S3.** UV-Vis absorption spectrum of VARH after 5 minutes of heating in a water bath at 37℃ and 44℃.


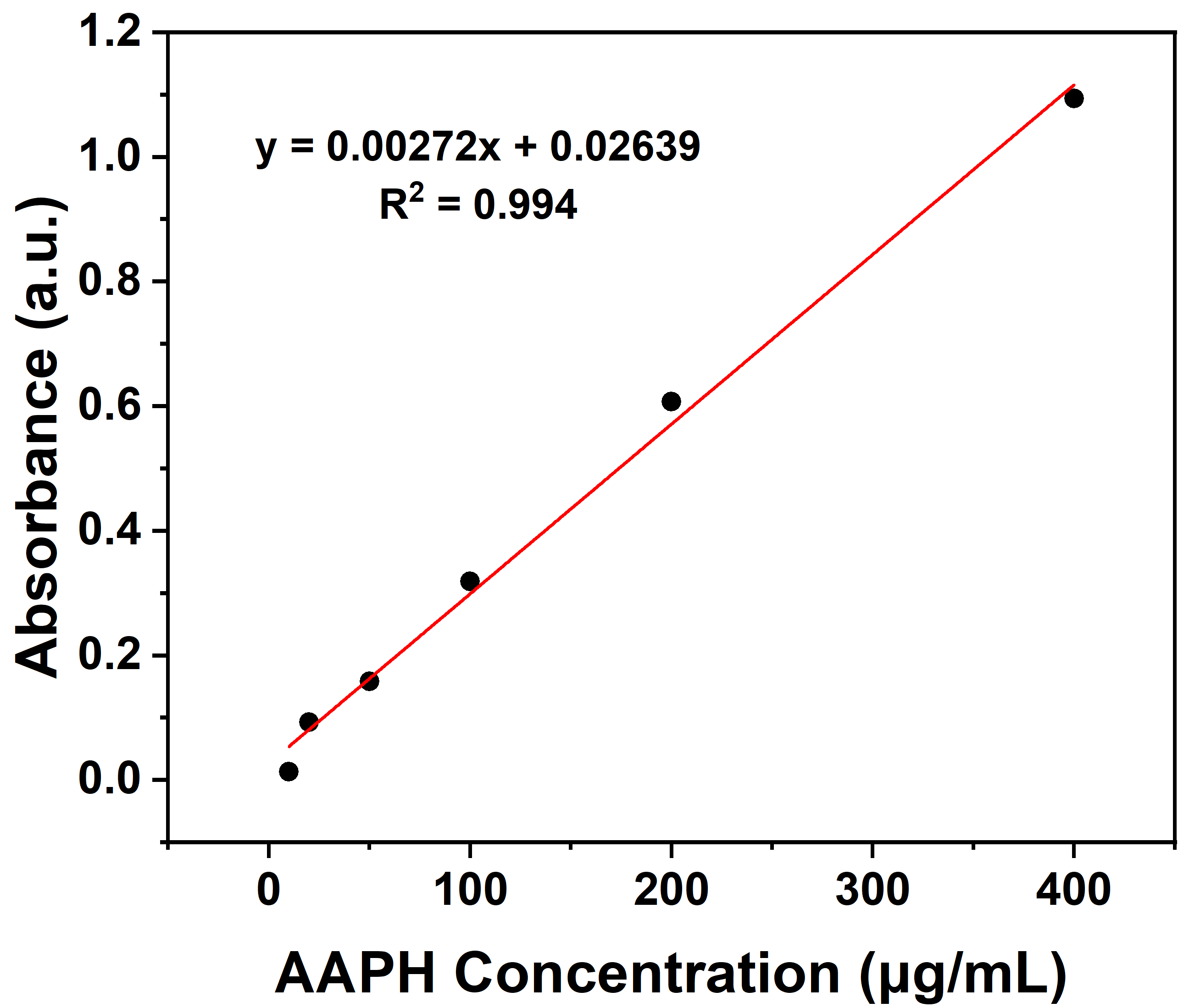


**Figure S4.** Standard curve for AAPH concentration determination.


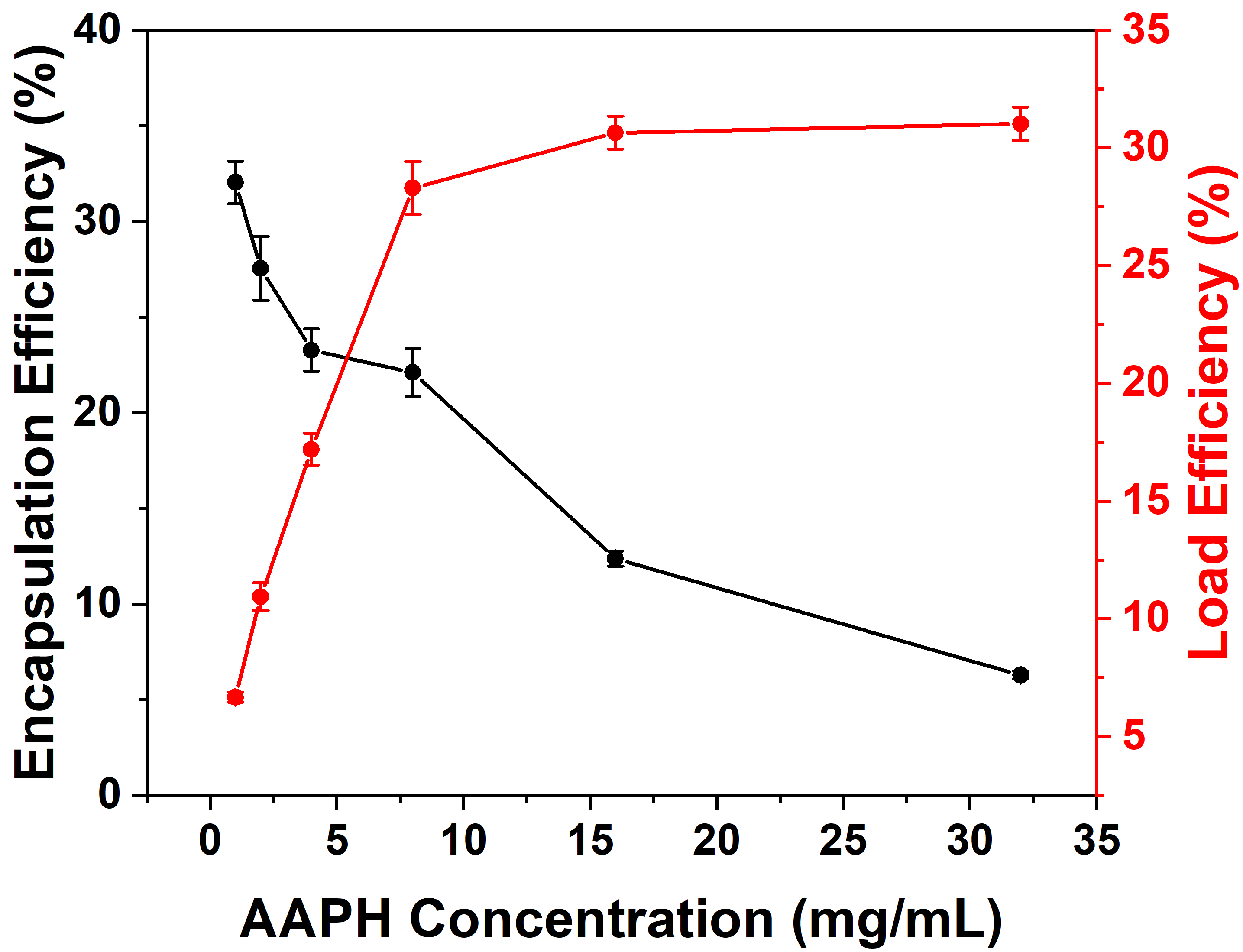


**Figure S5.** Effects of different feeding amounts on AAPH EE and LE.


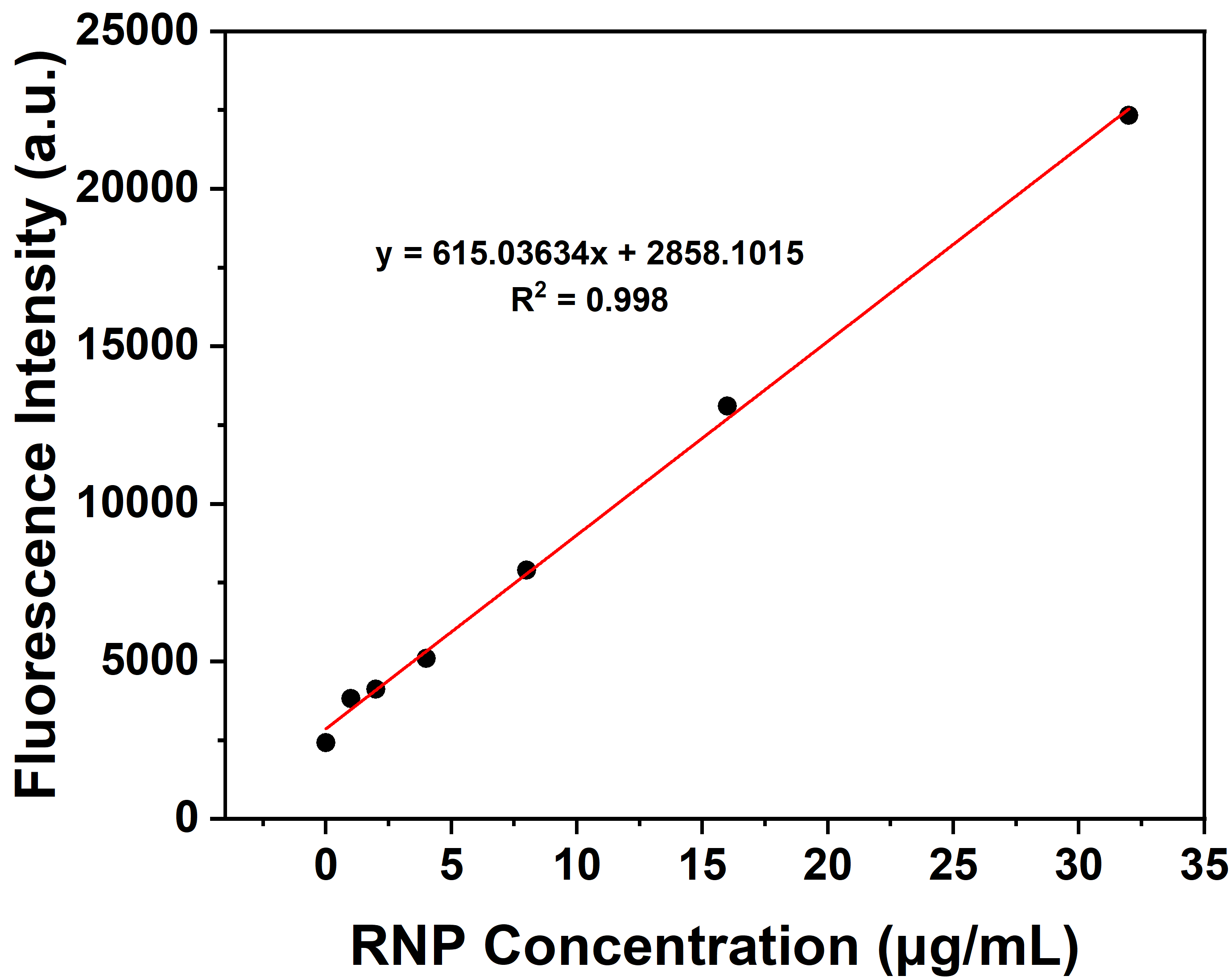


**Figure S6.** Standard curve for RNP complex concentration determination.


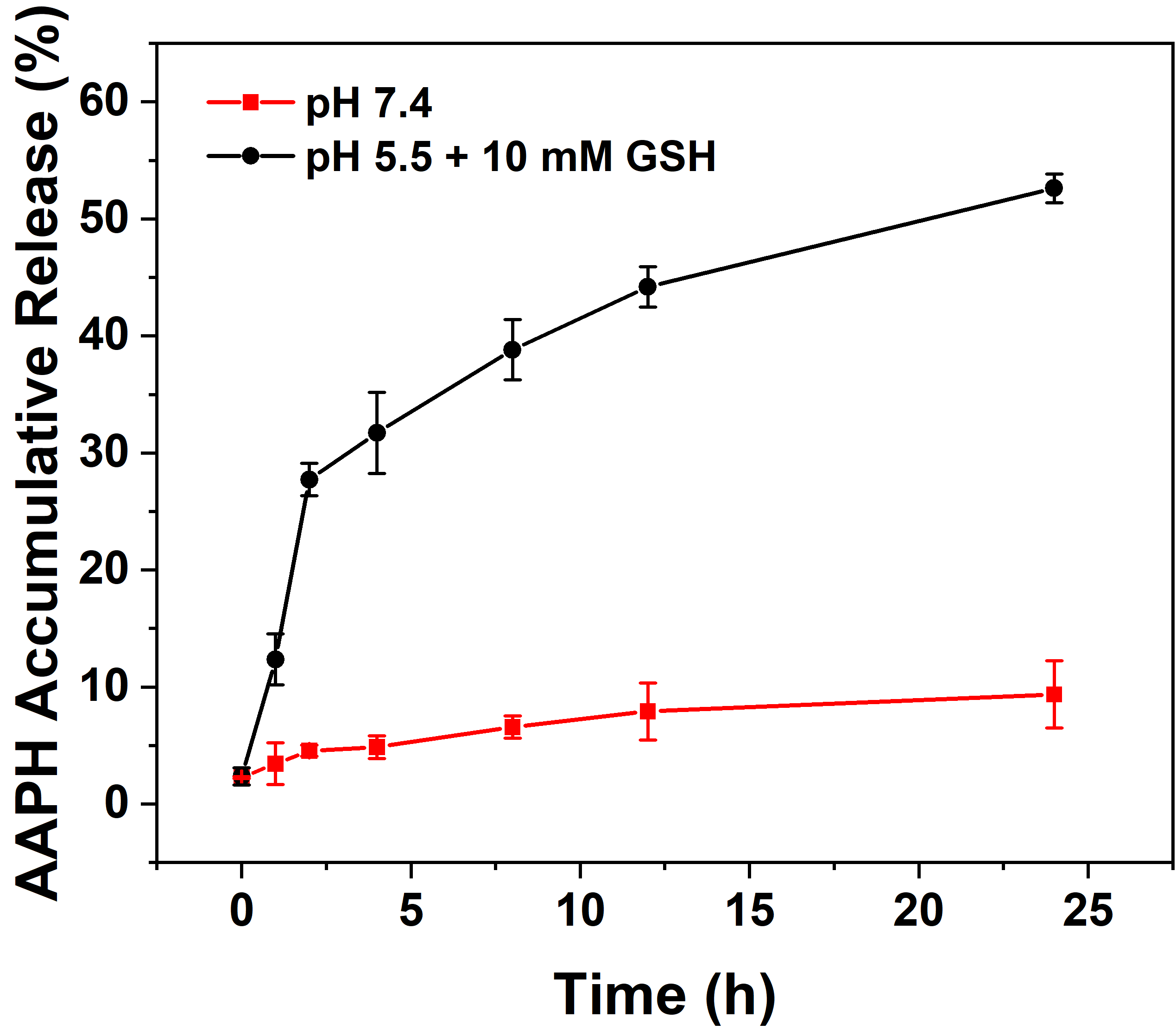


**Figure S7.** AAPH in VARH under different environments.


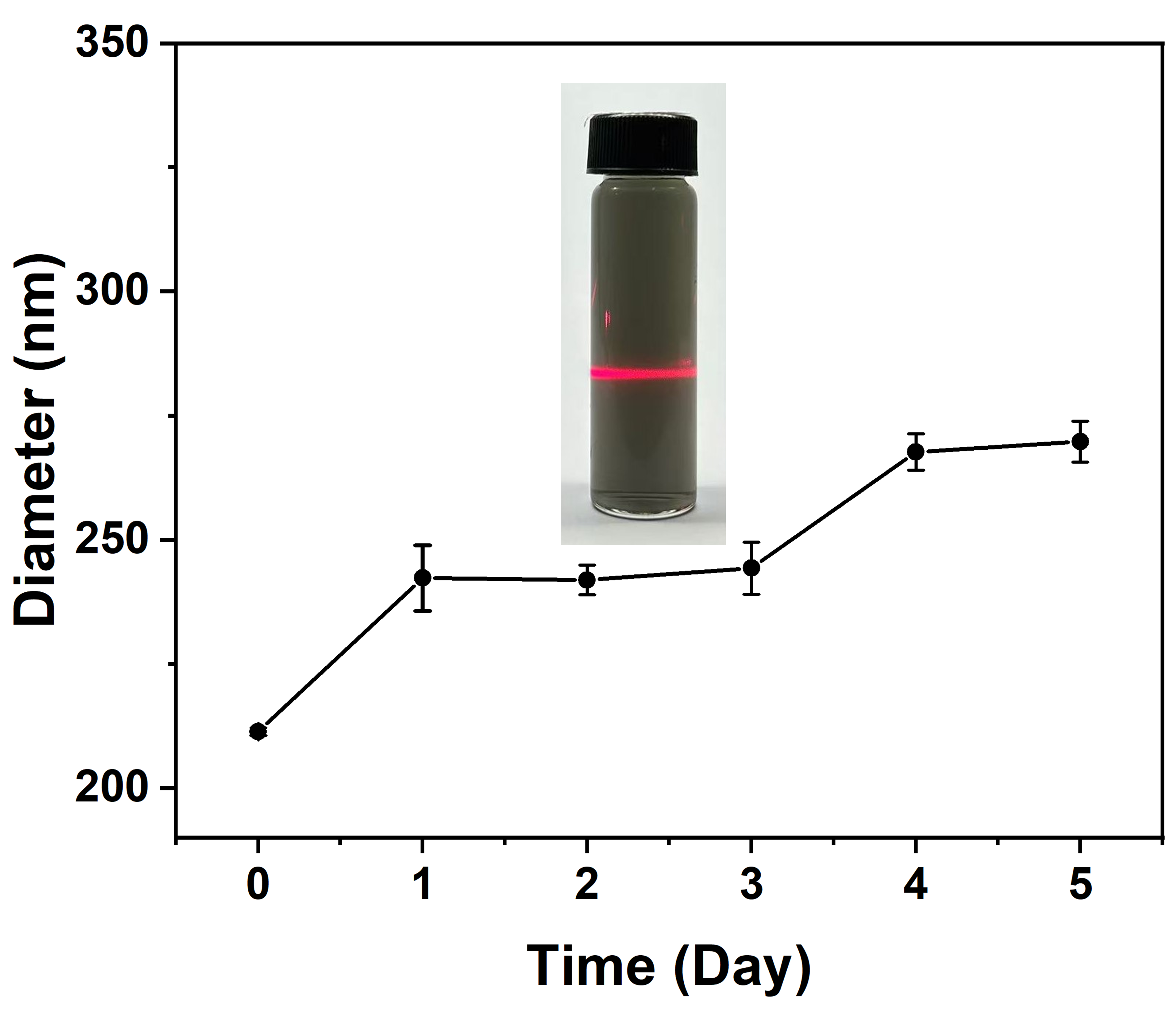


**Figure S8.** Particle size variation and tyndall effect of VARH dispersed in water over 5 days.


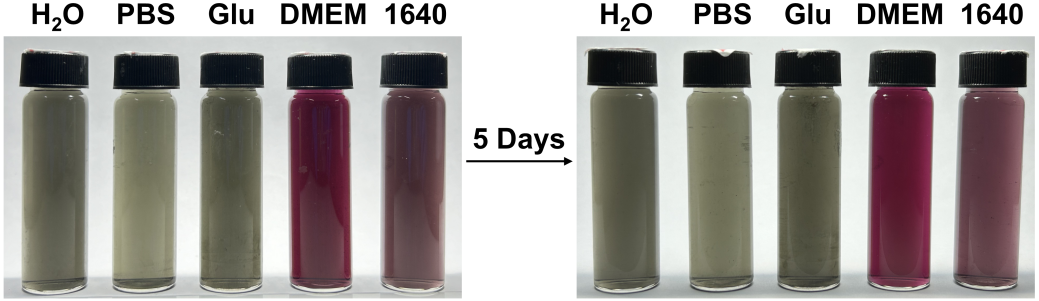


**Figure S9.** Dispersion of VARH in different media over 5 days.


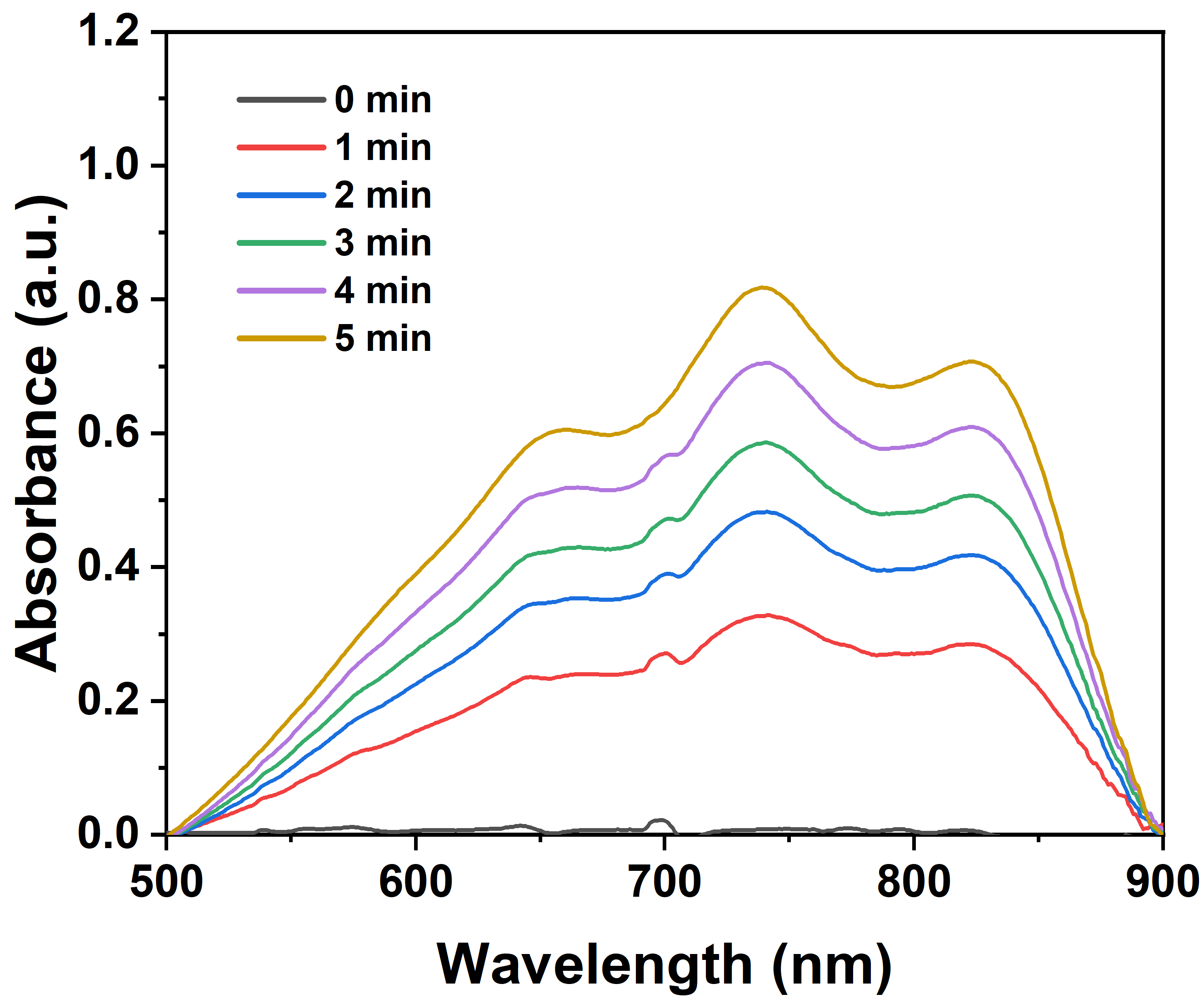


**Figure S10.** UV-Vis absorption spectrum of VARH after 5 minutes of irradiation with 1064 nm laser.


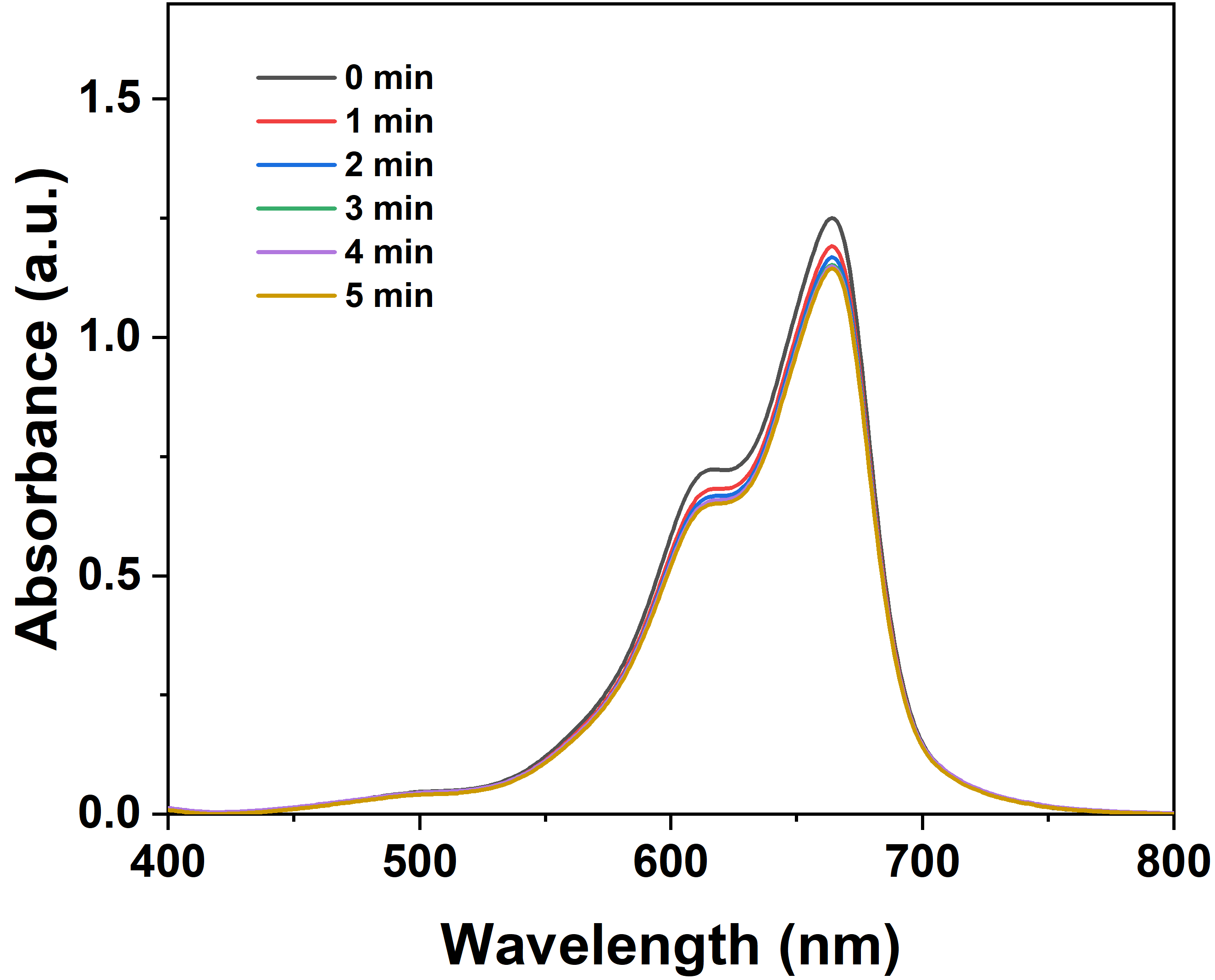


**Figure S11.** UV-Vis absorption spectrum of VARH without light irradiation after a certain period of time.


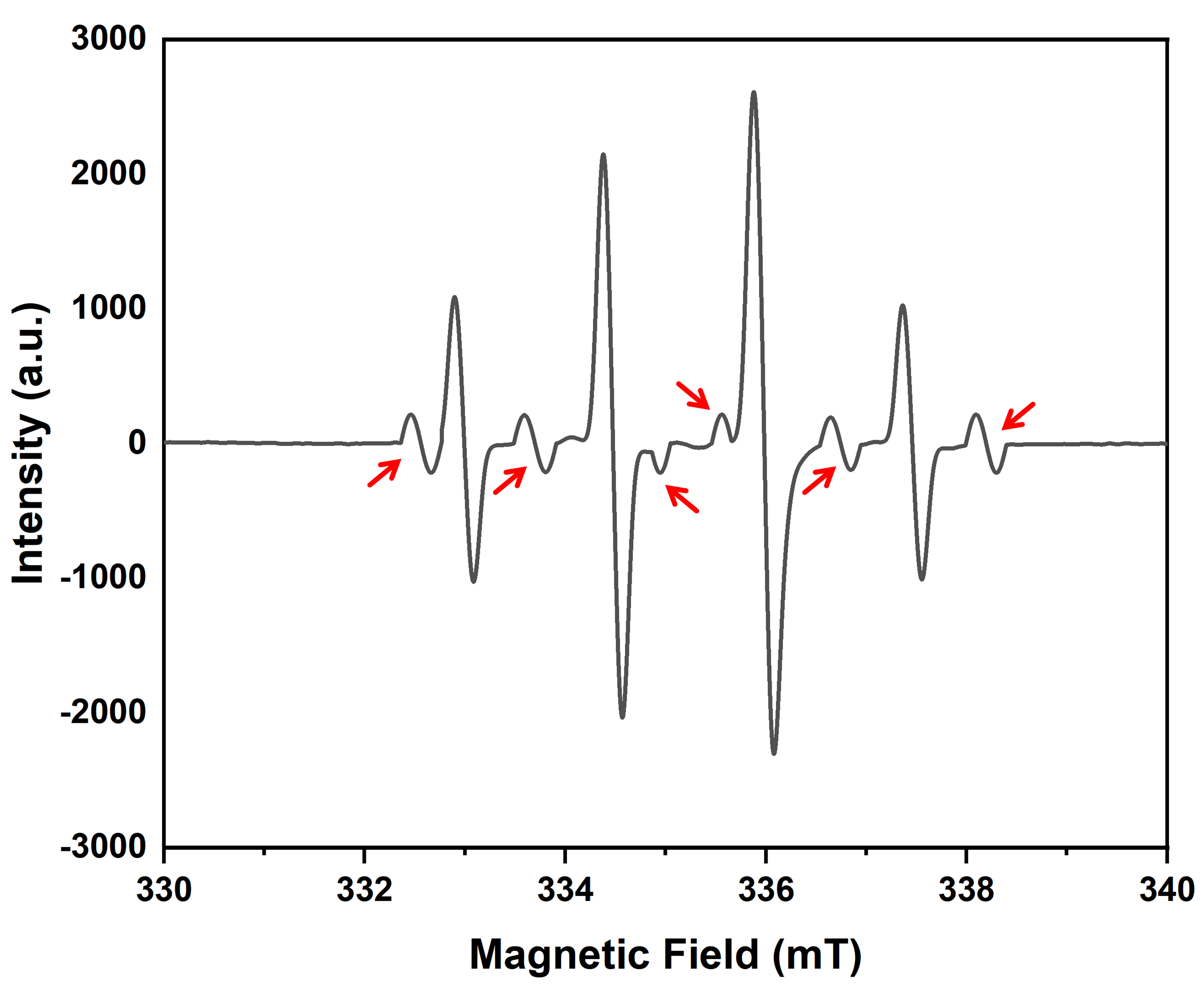


**Figure S12.** The ESR spectrum indicating alkyl radicals in VARH.


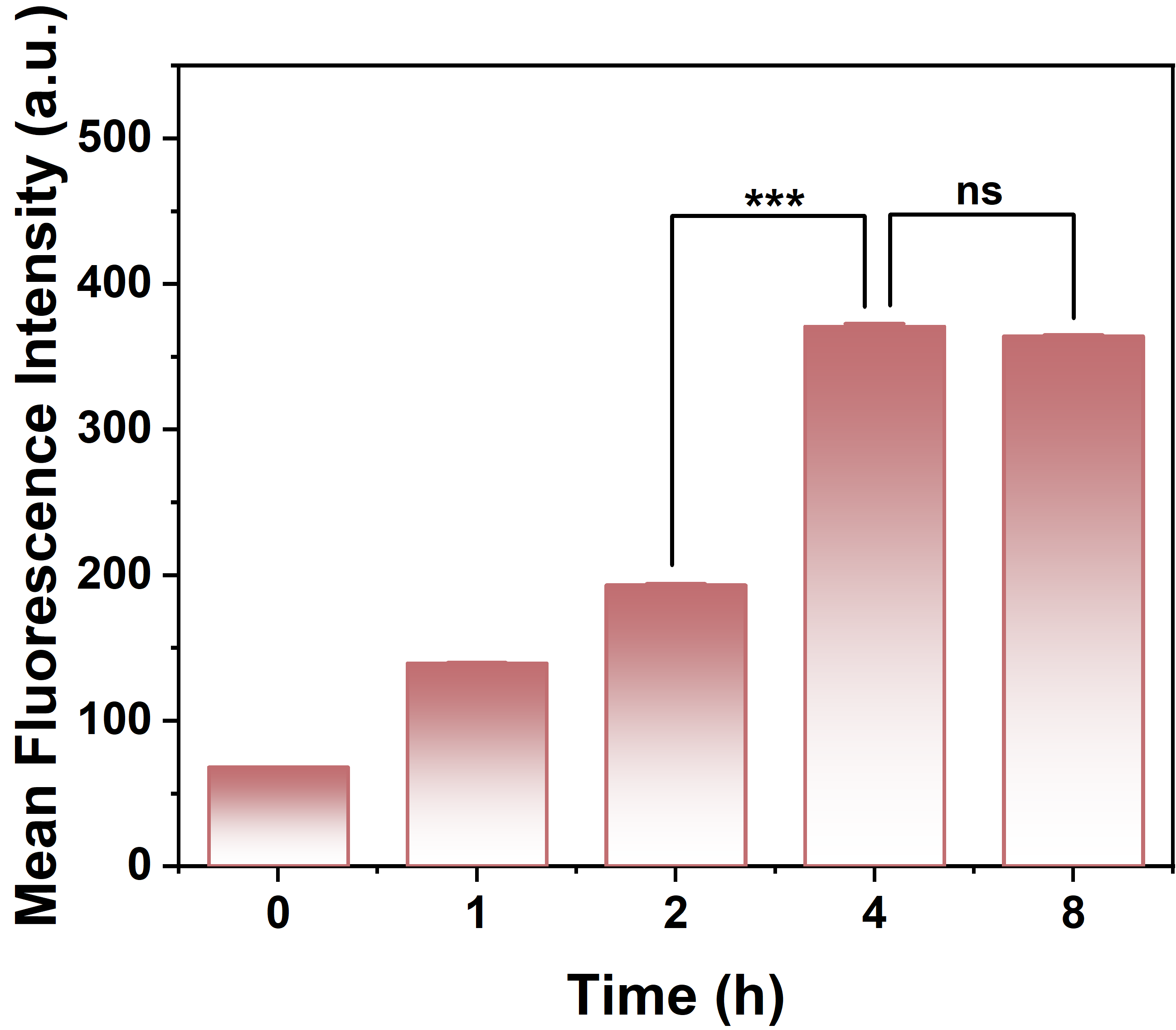


**Figure S13.** The average fluorescence intensity of FCM results for VARH uptake in HepG2 cells at different times.


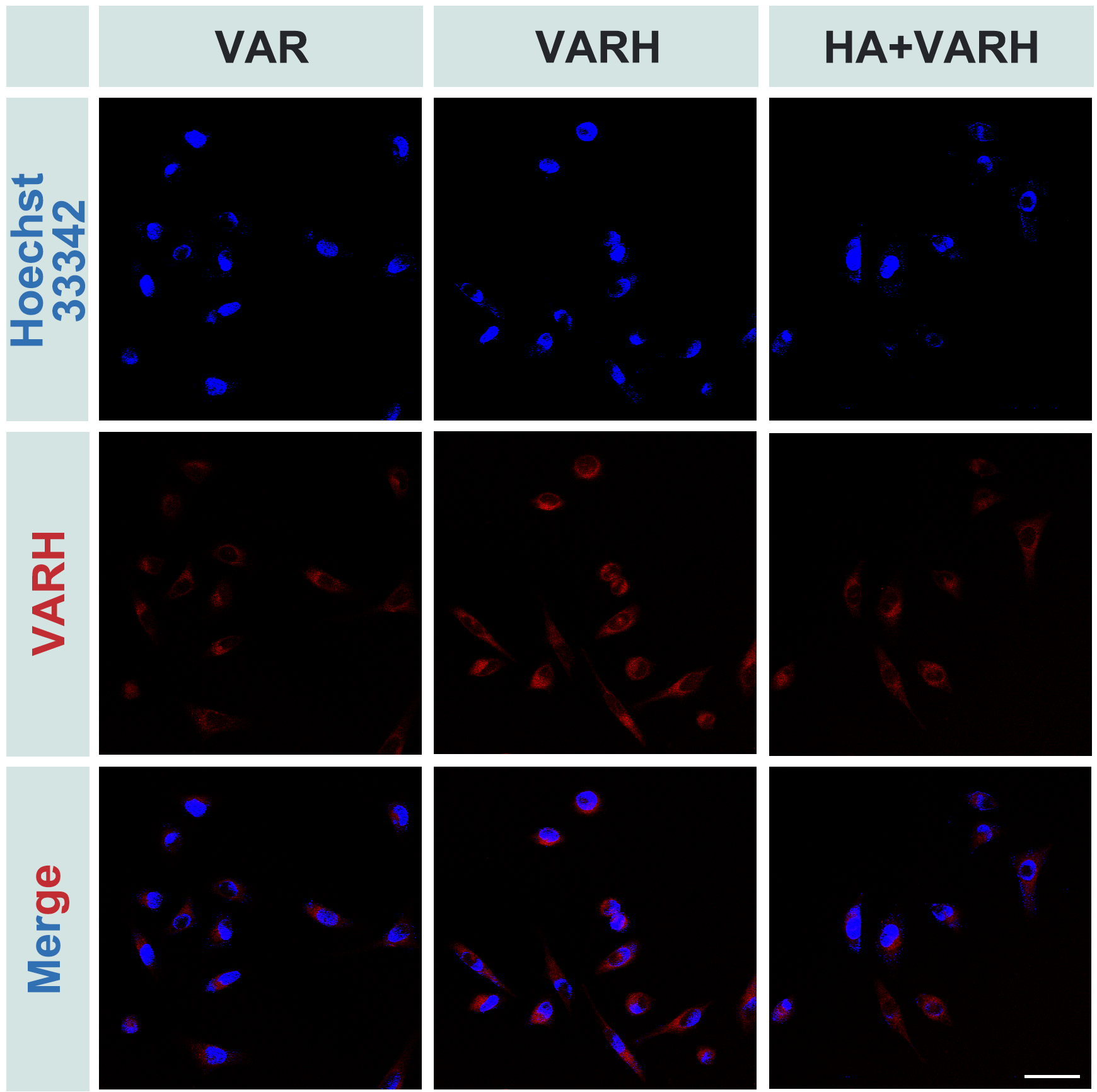


**Figure S14.** CLSM images of cellular uptake of VARH under free HA competition or in a low CD44-expressing environment, scale bar = 50 μm.


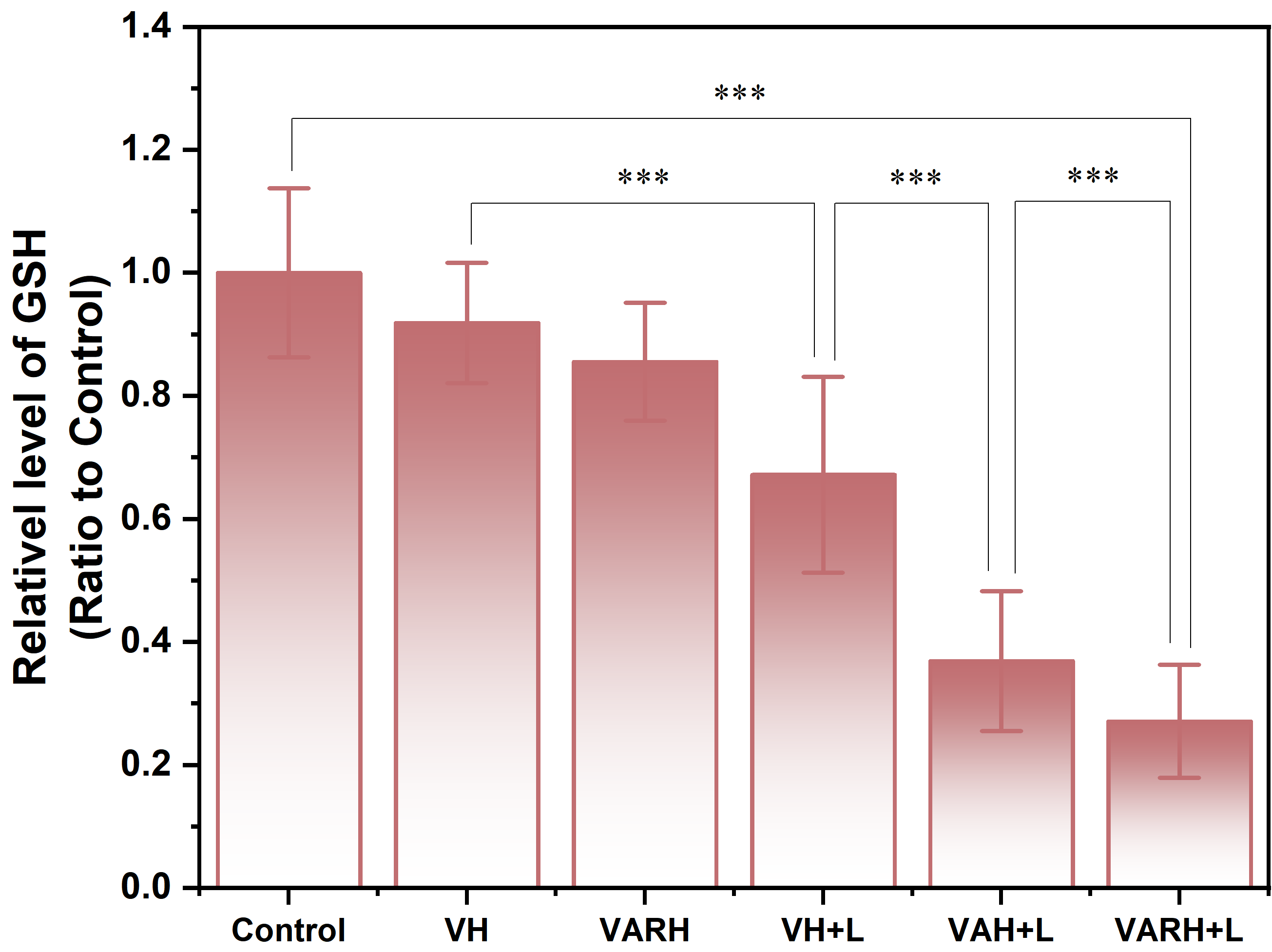


**Figure S15.** GSH depletion in HepG2 cells across different treatment groups.


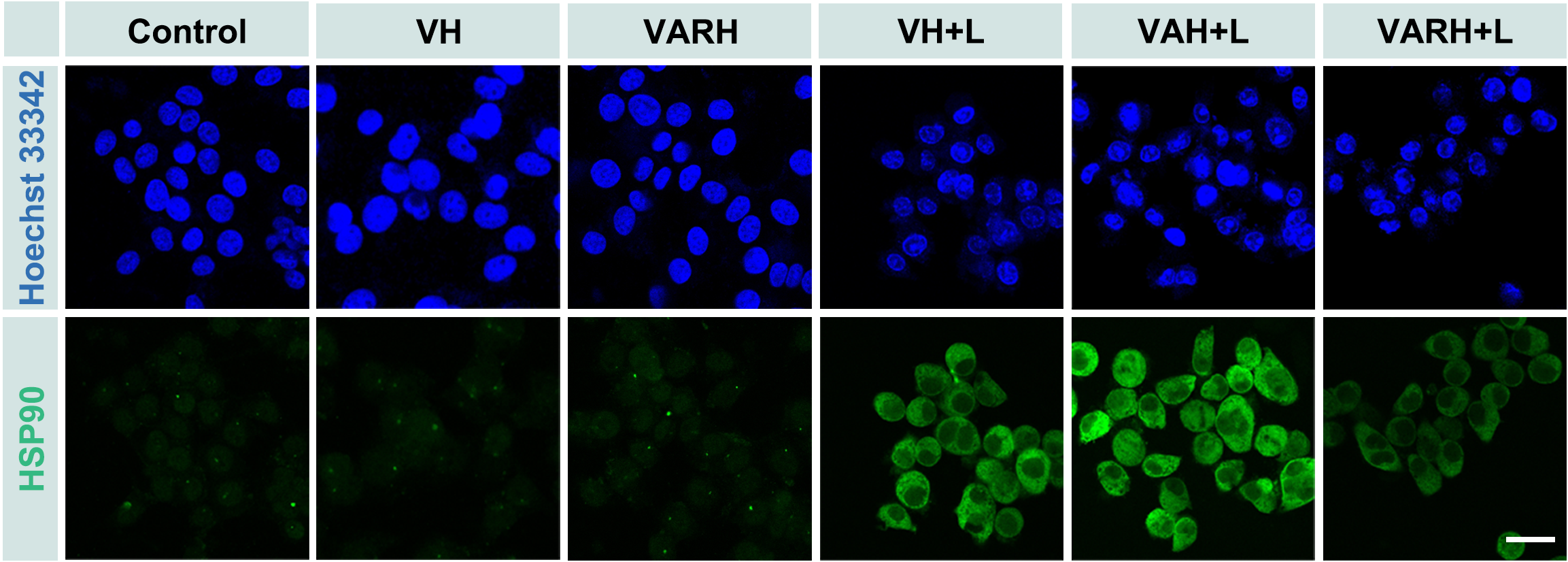


**Figure S16.** Immunofluorescence images of HSP90 protein in HepG2 cells after treatment with different formulations, scale bar = 30 μm.


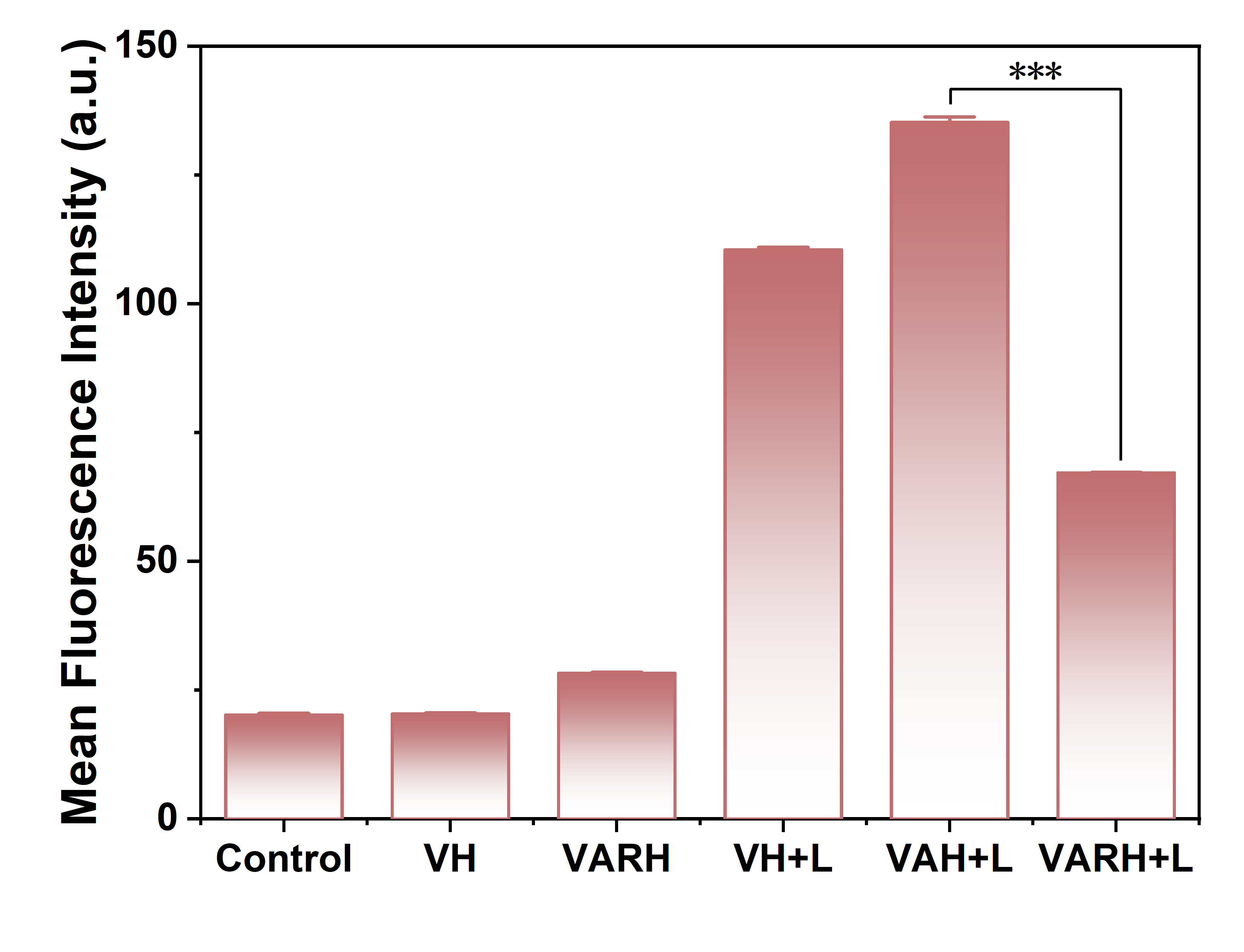


**Figure S17.** Immunofluorescence Quantitative Analysis of HSP90 Protein in HepG2 Cells After Treatment with Different Administration Groups.


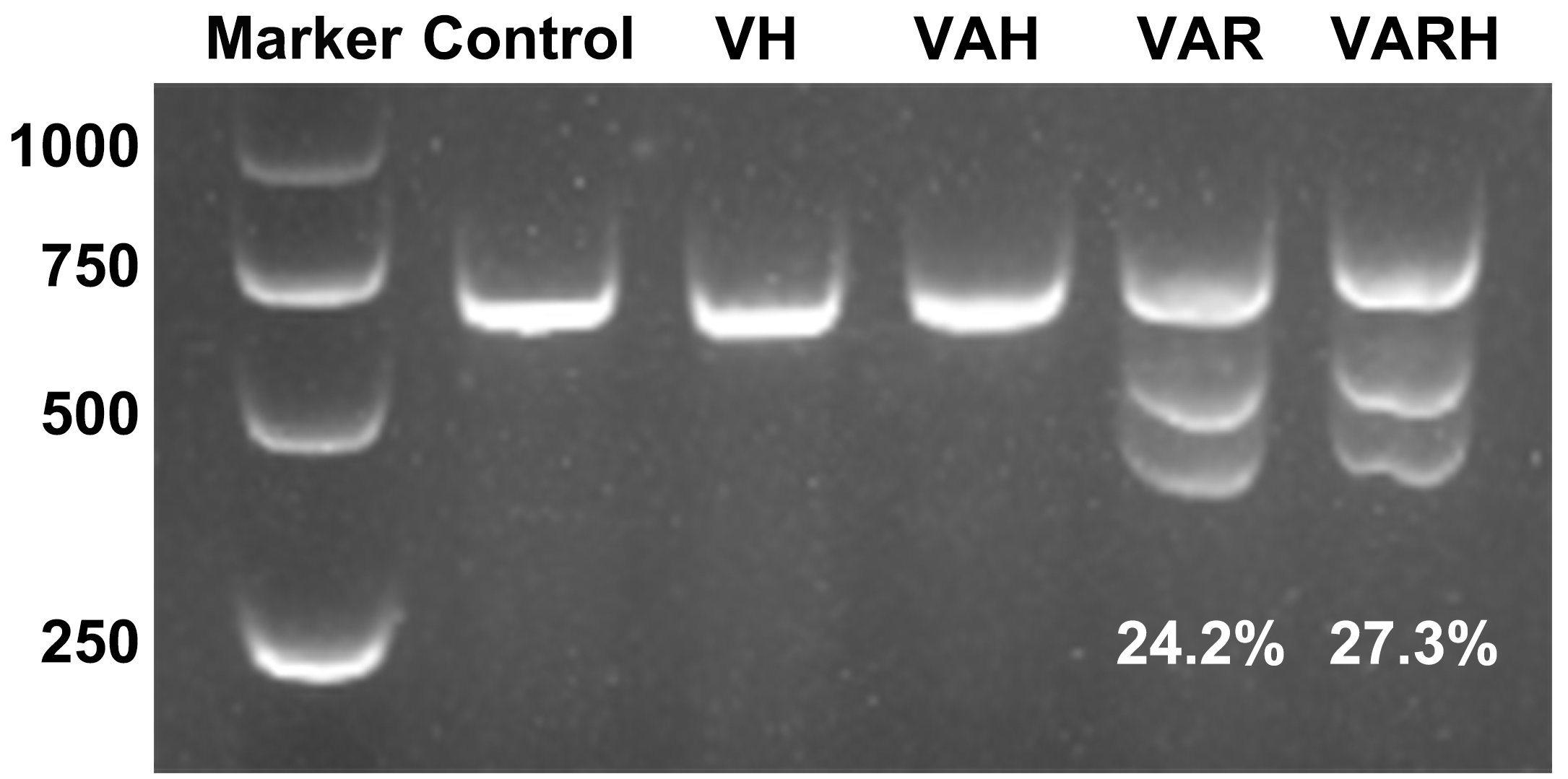


**Figure S18.** T7EⅠ assay for gene editing efficiency of HSP90 gene by different nanodrugs.


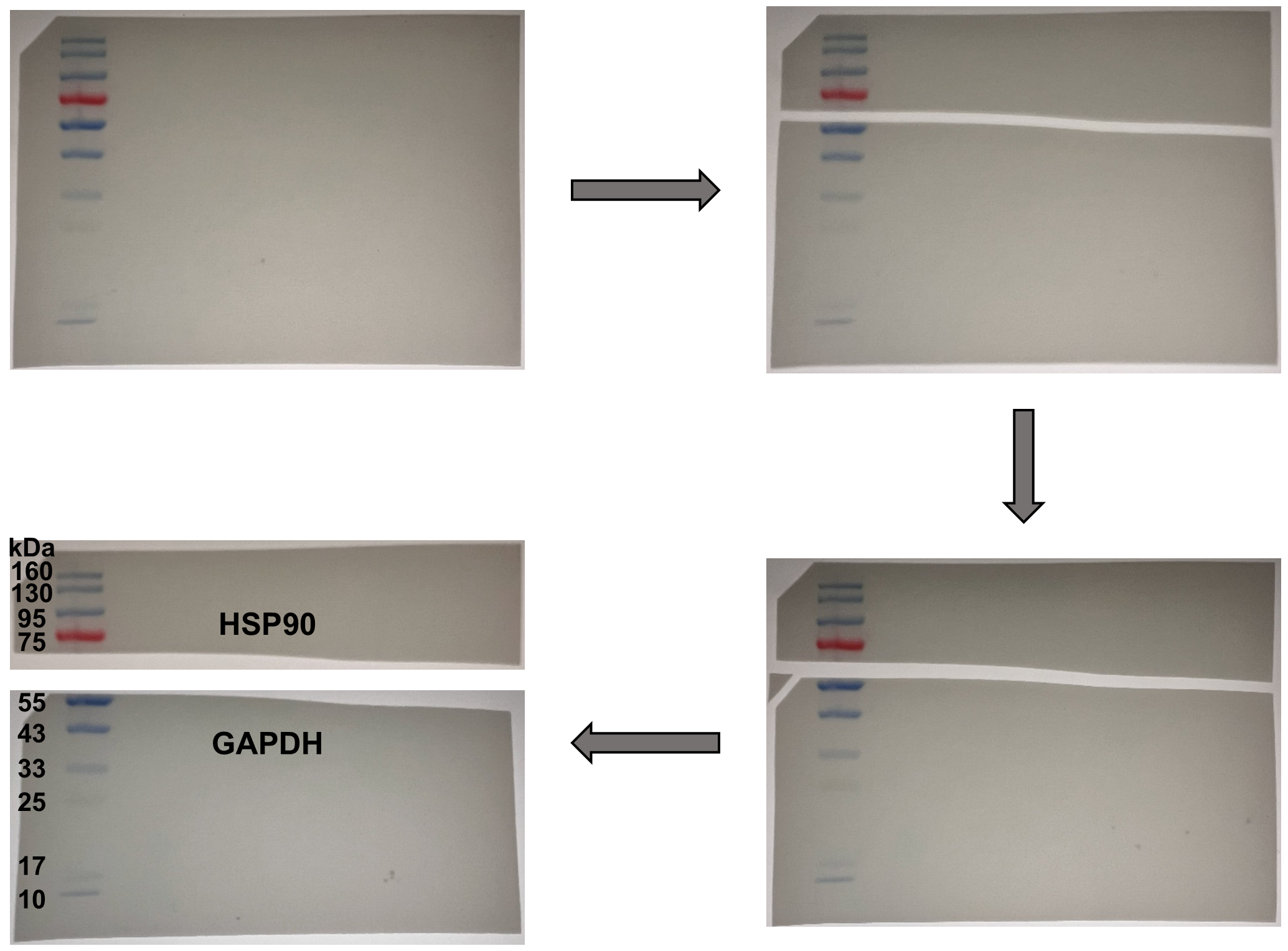


**Figure S19.** The membrane cropping process of Western blot analysis for HSP90 protein expression in HepG2 cells treated with non-targeting sgRNA, 17‑AAG inhibitor, or VARH + L.


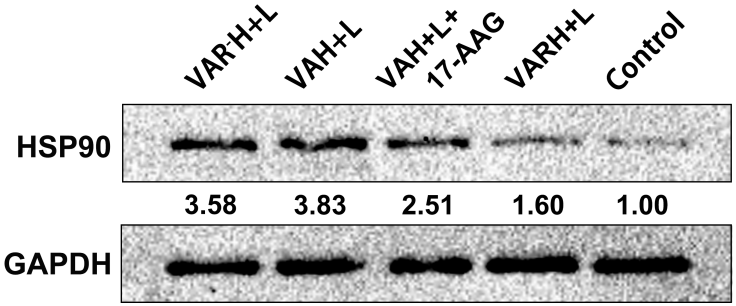


**Figure S20.** Western blot analysis of HSP90 protein expression levels in HepG2 cells treated with non-targeting sgRNA, the 17-AAG inhibitor, or VARH+L.


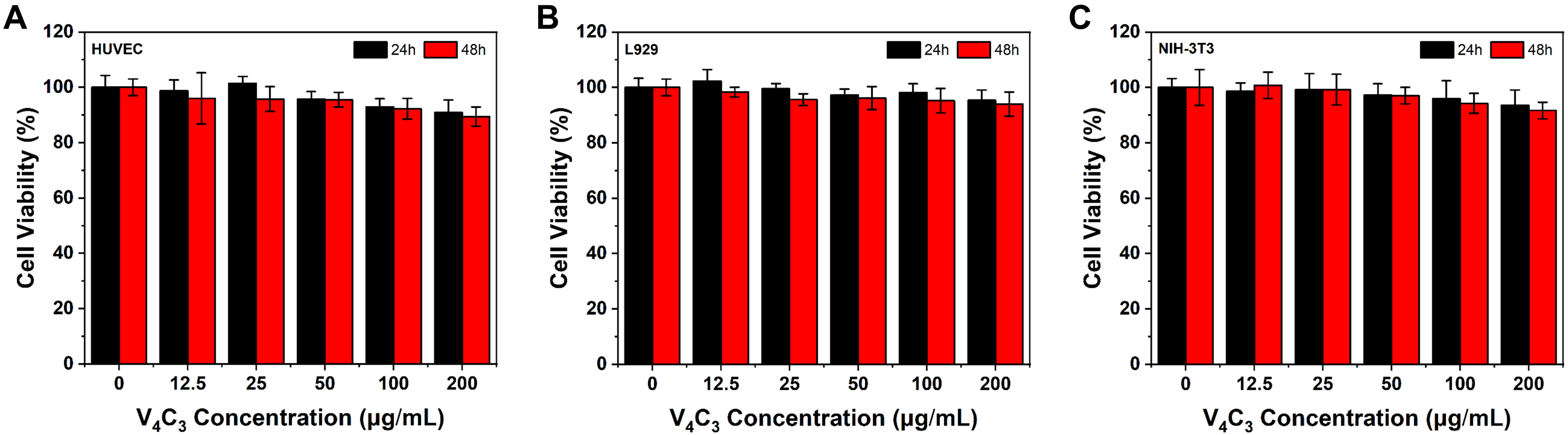


**Figure S21.** Cytotoxicity assay in HUVEC, L929, and NIH-3T3 cells treated with various concentrations.


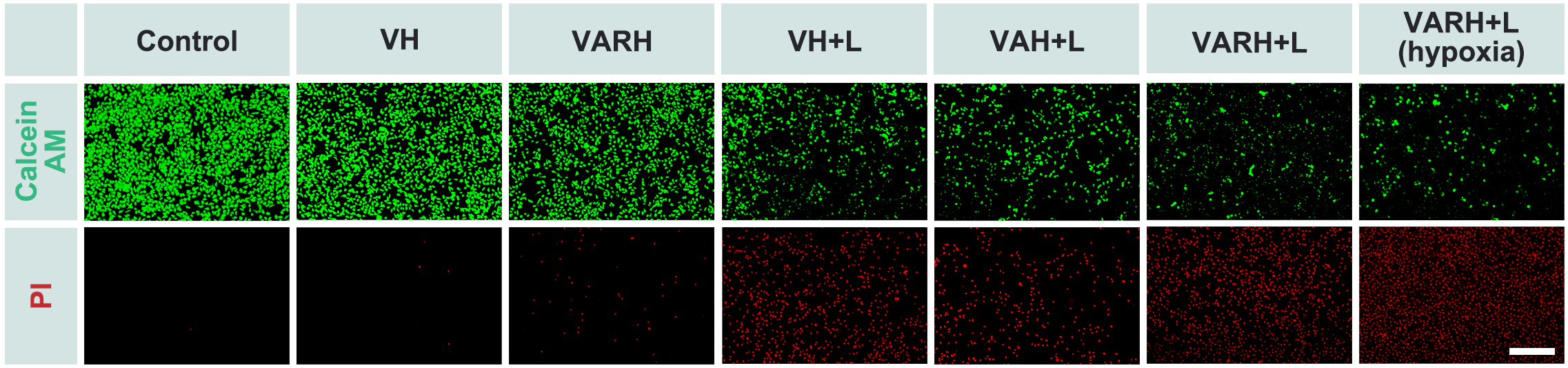


**Figure S22.** Live/dead cell staining images of HepG2 cells treated with different nanodrug formulations,scale bar = 200 μm.


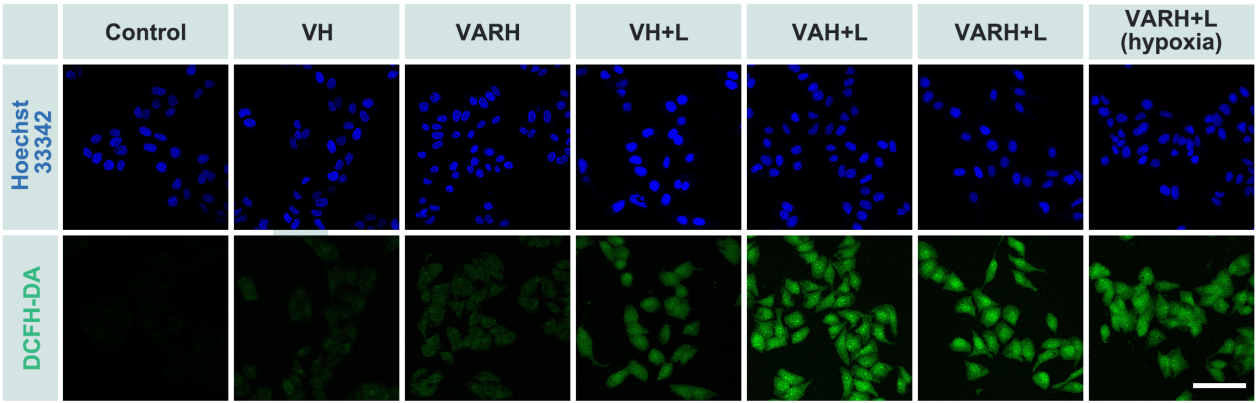


**Figure S23.** CLSM images of reactive oxygen species (ROS) generation in different treatment groups,scale bar = 100 μm.


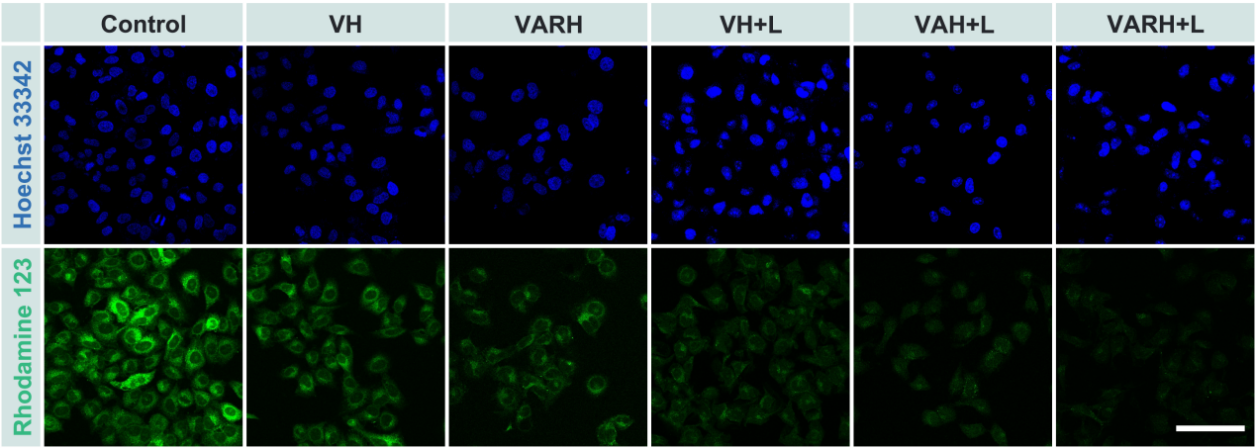


**Figure S24.** CLSM images of mitochondrial membrane potential changes in HepG2 cells induced by different treatment groups, scale bar = 100 μm.


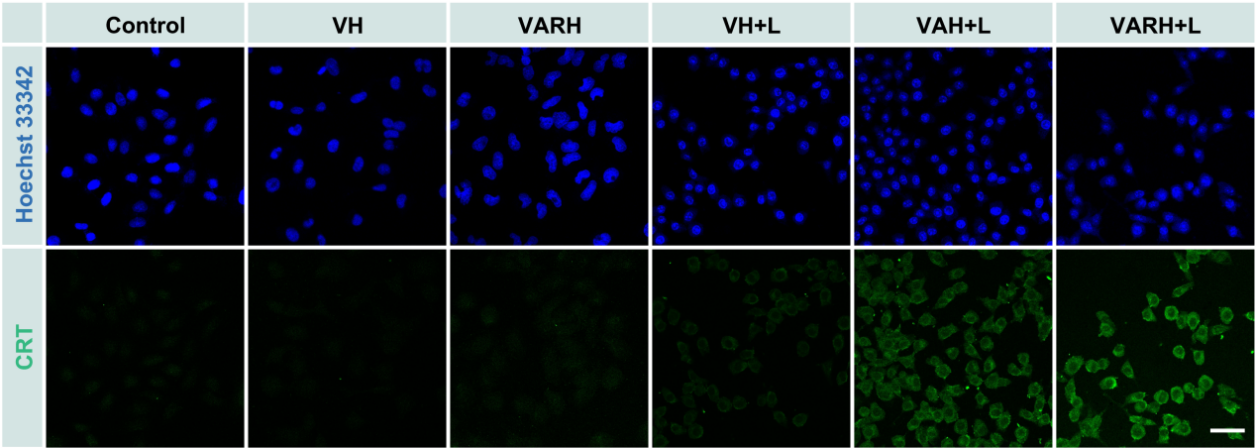


**Figure S25.** CRT exposure in HepG2 cells after different treatment, scale bar = 50 μm.


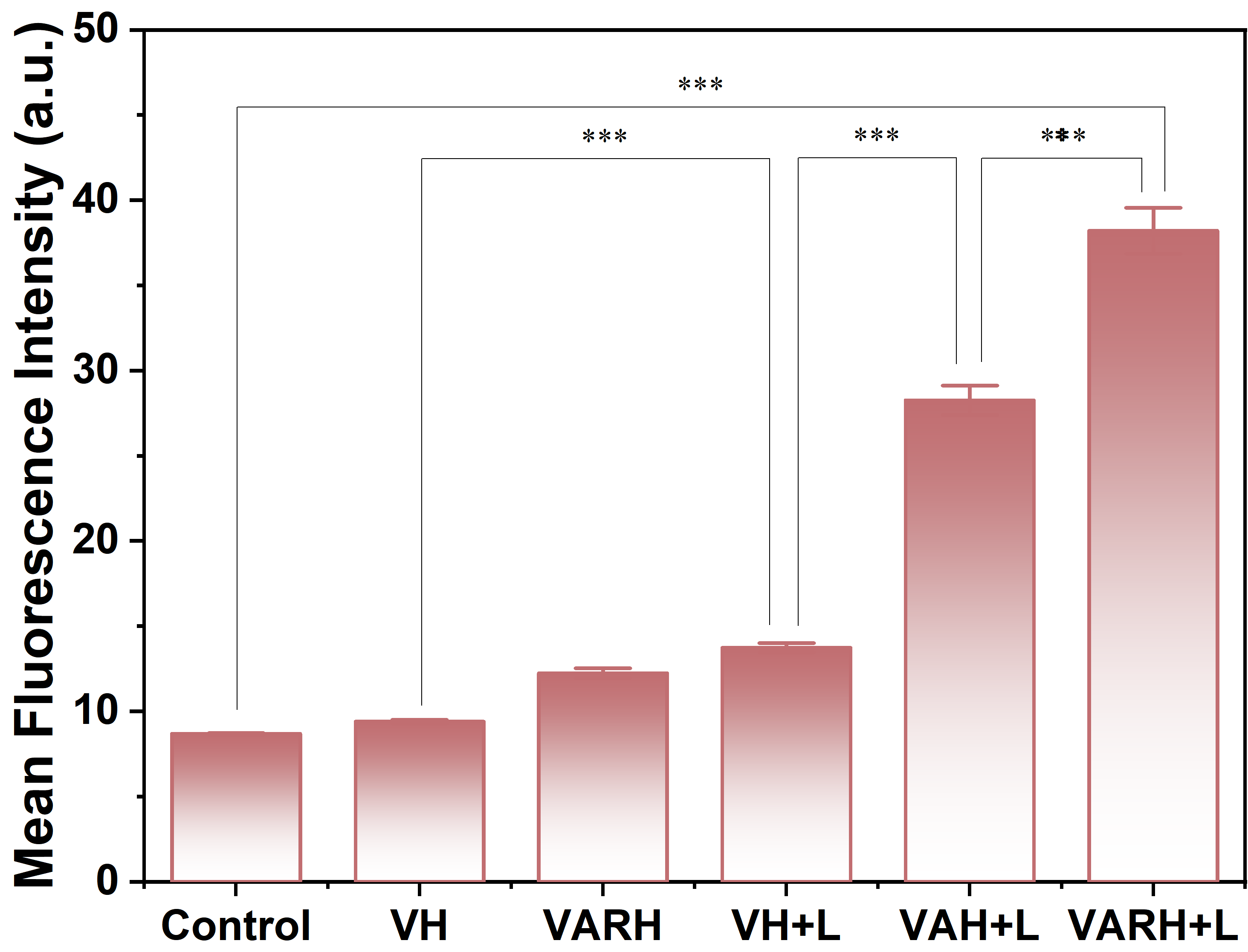


**Figure S26.** CRT exposure was quantitatively evaluated in HepG2 cells after various treatments.


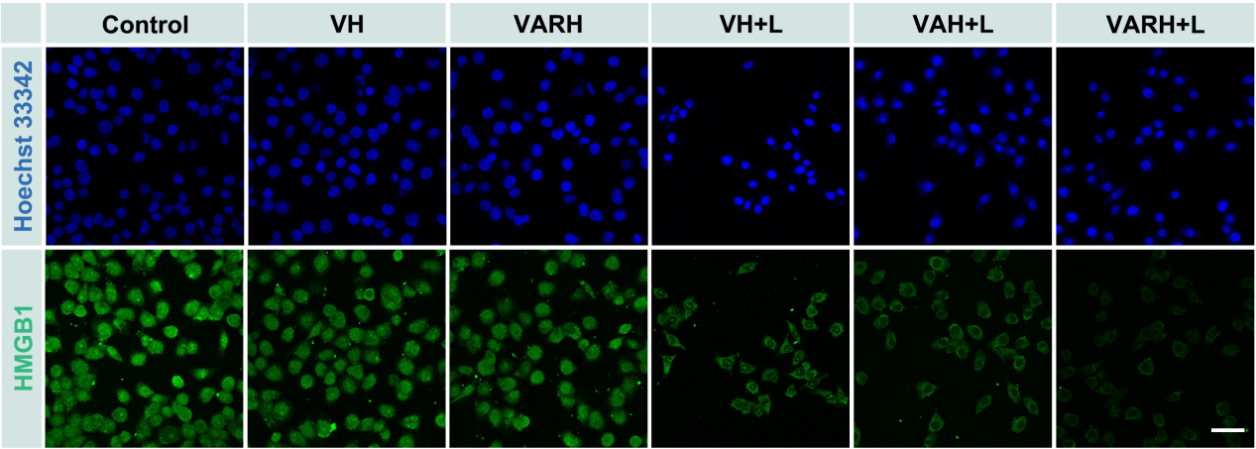


**Figure S27.** Release of HMGB1 in HepG2 cells after different treatment, scale bar = 50 μm.


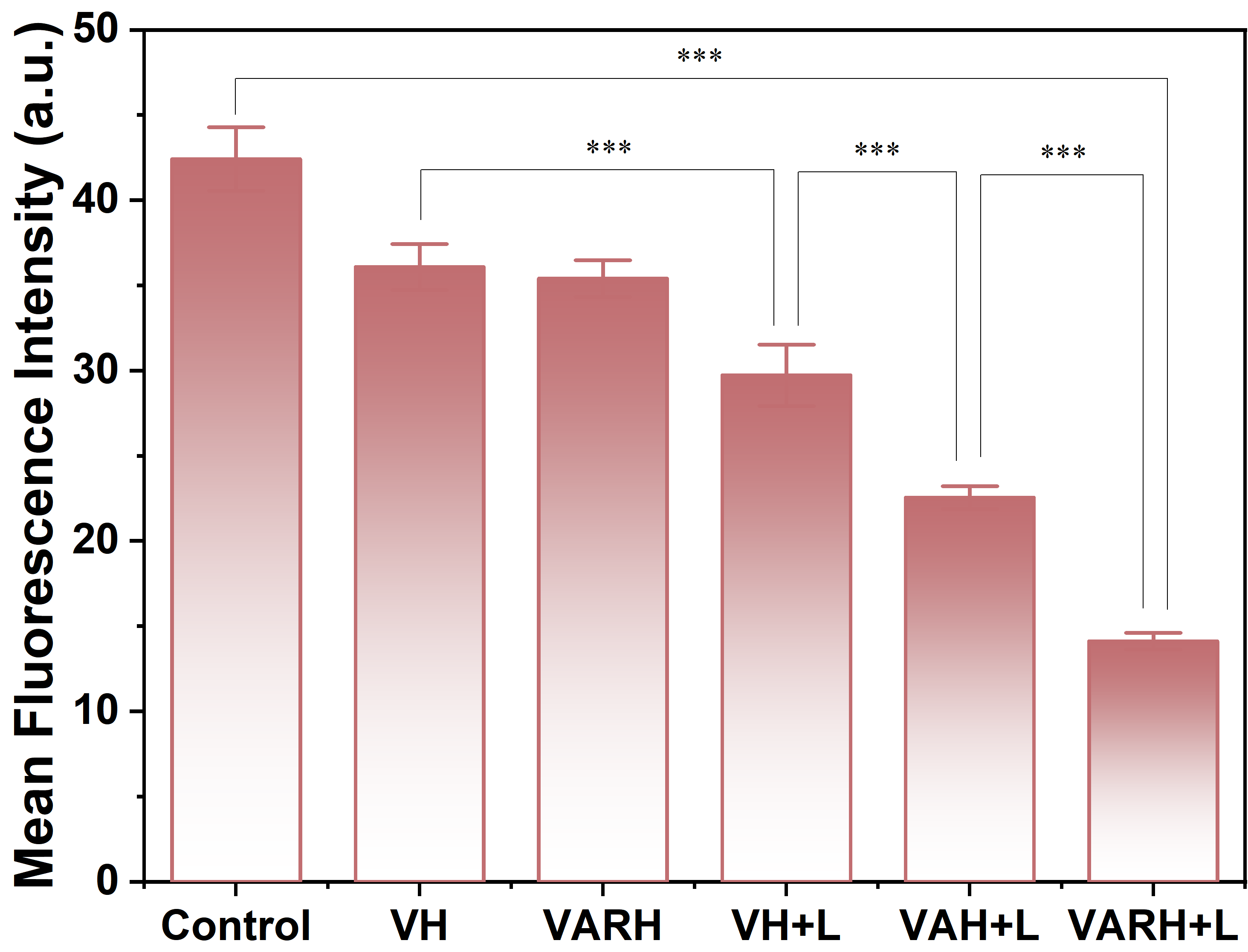


**Figure S28.** The release of HMGB1 was quantified in HepG2 cells following various treatments.


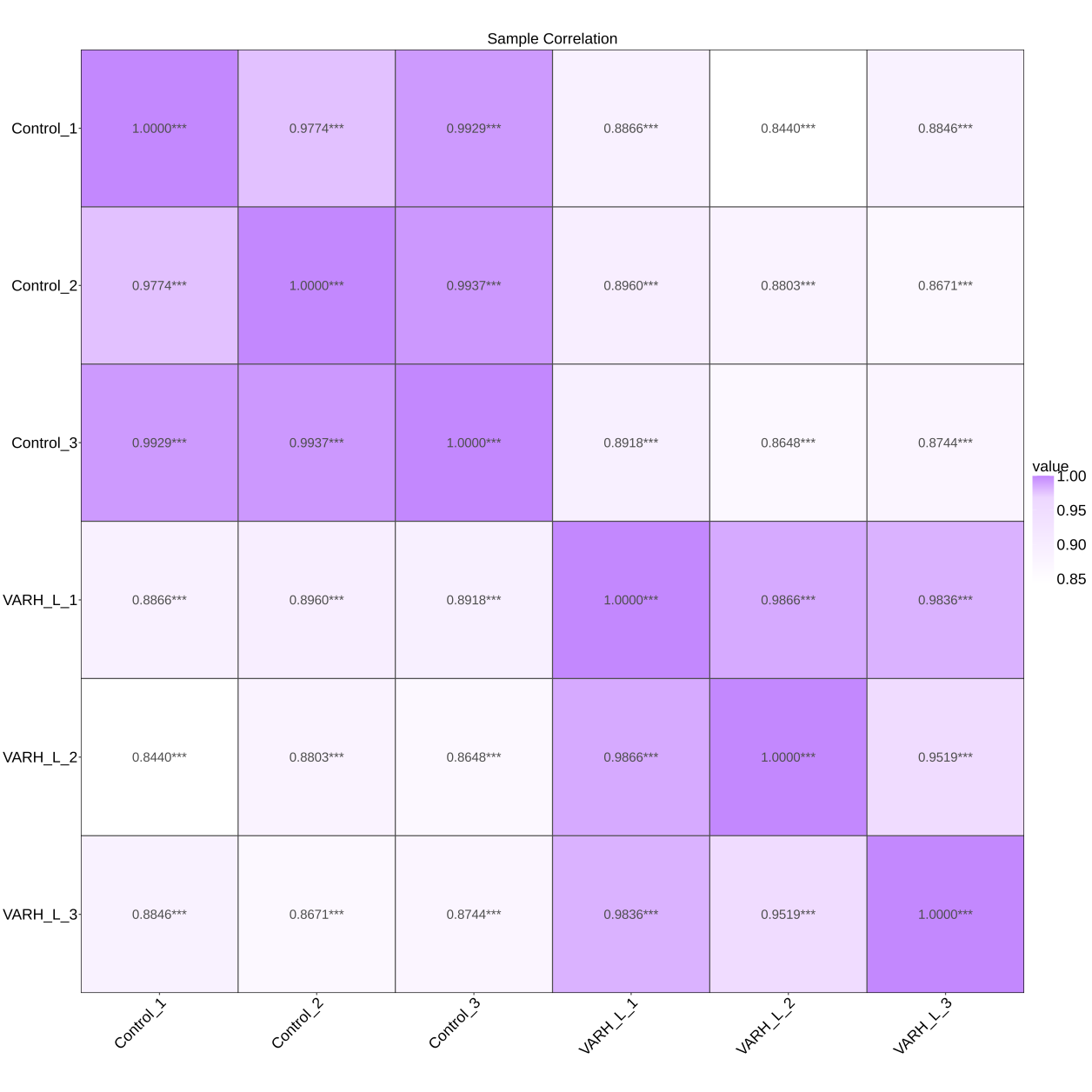


**Figure S29.** Heatmap showing correlations between samples. (Note: "VARH_L_1" denotes "VARH+L-1" in this and all subsequent instances, owing to a systematic formatting constraint.)


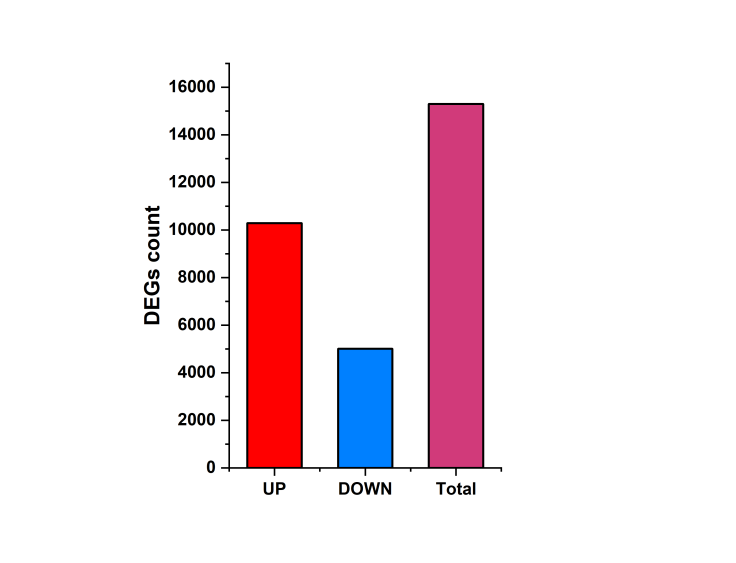


**Figure S30.** The number of differentially expressed genes identified in VARH+L versus Control.


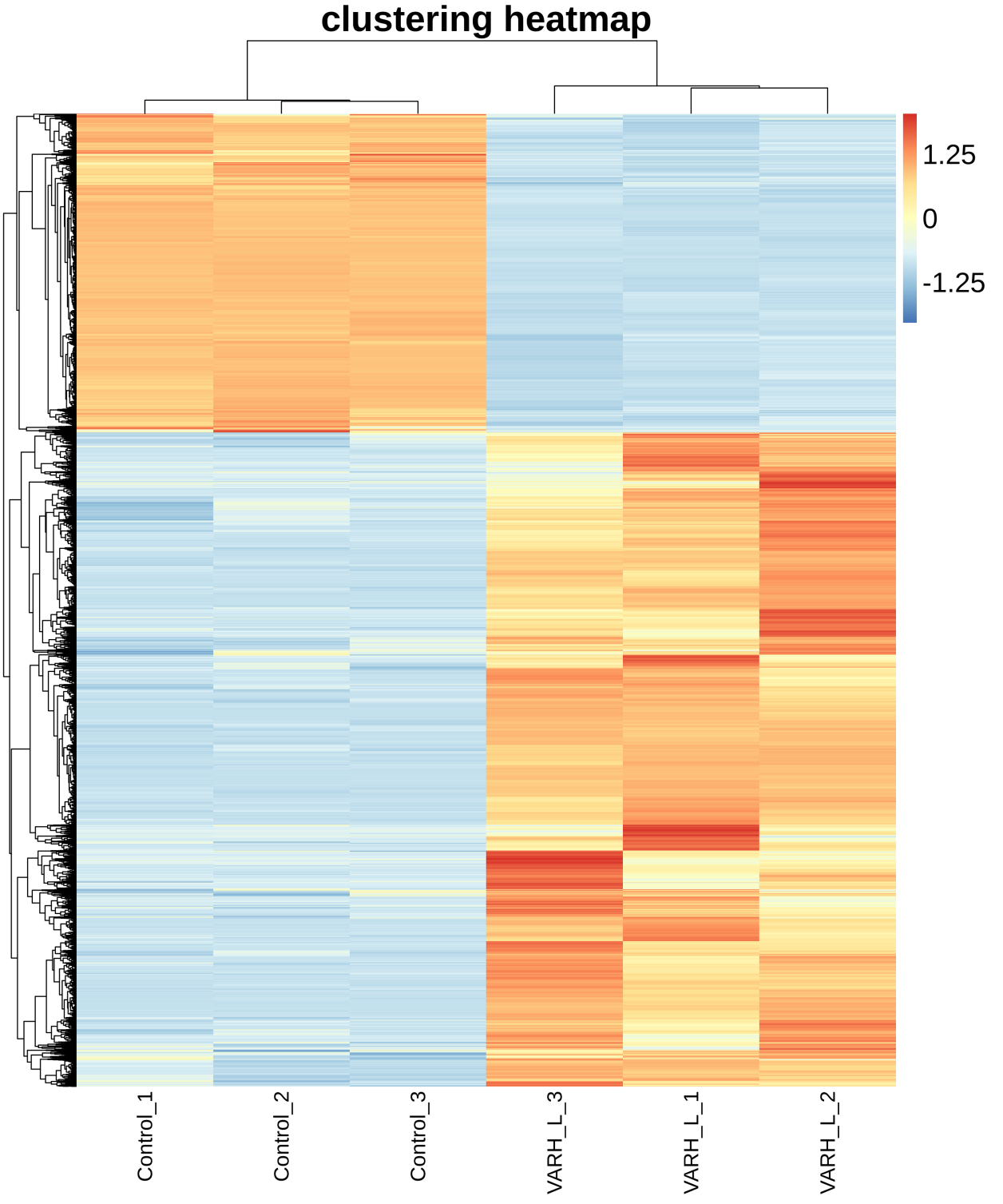


**Figure S31.** Differentially Expressed Genes (DEGs) clustering heatmap in VARH+L versus Control.


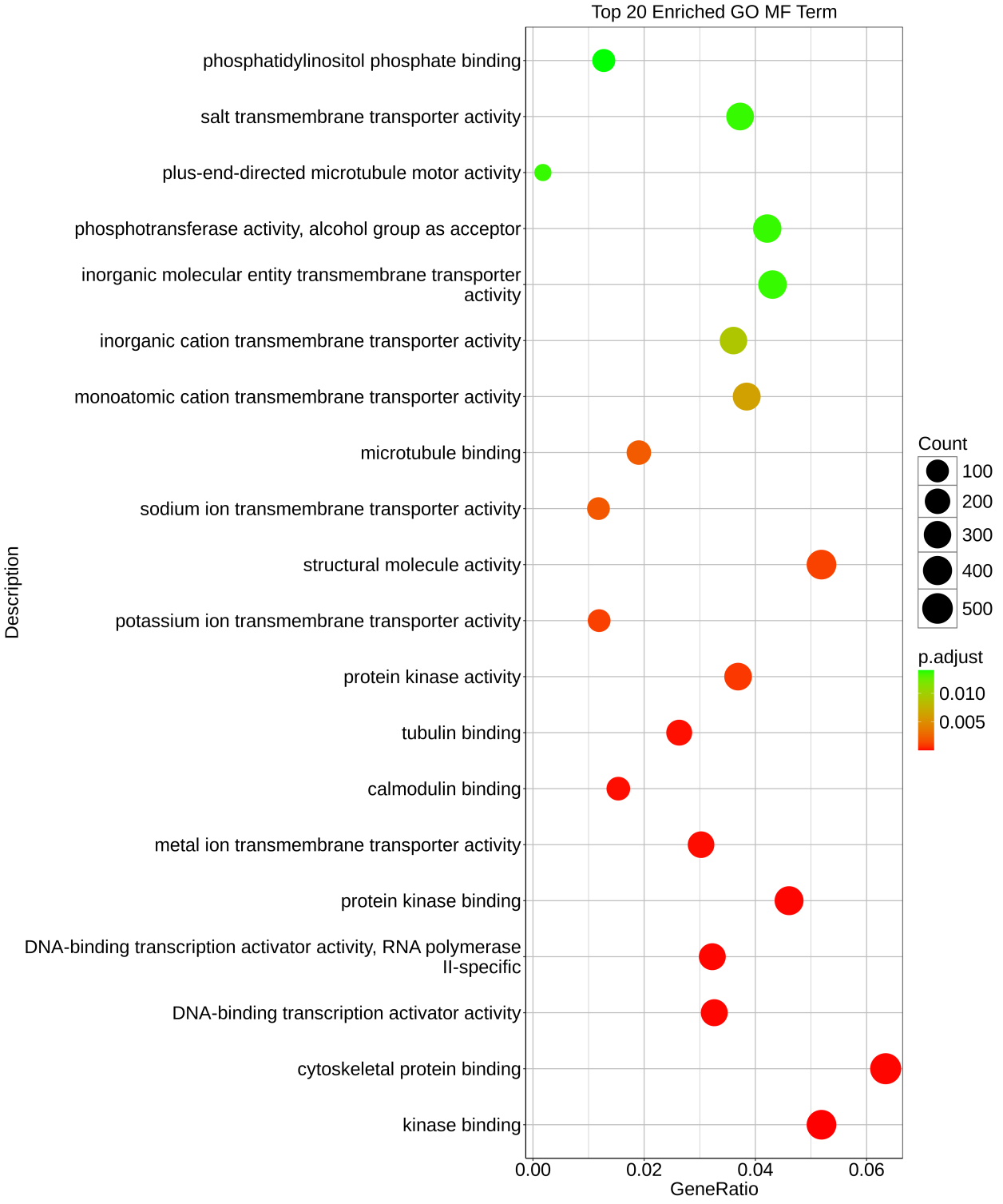


**Figure S32.** Top 20 Enriched GO Terms (Molecular Function).


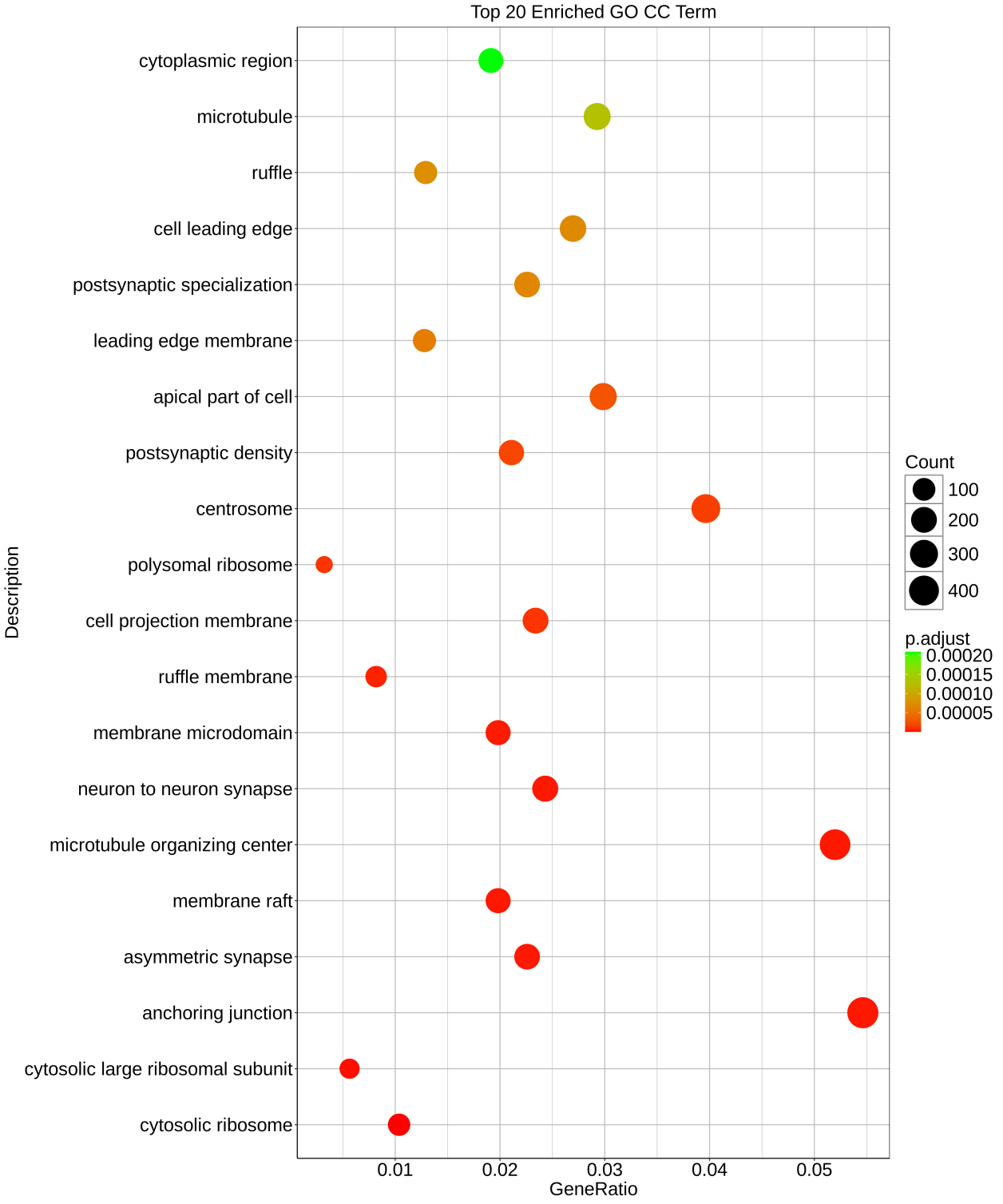


**Figure S33.** Top 20 Enriched GO Terms (Cellular Component).


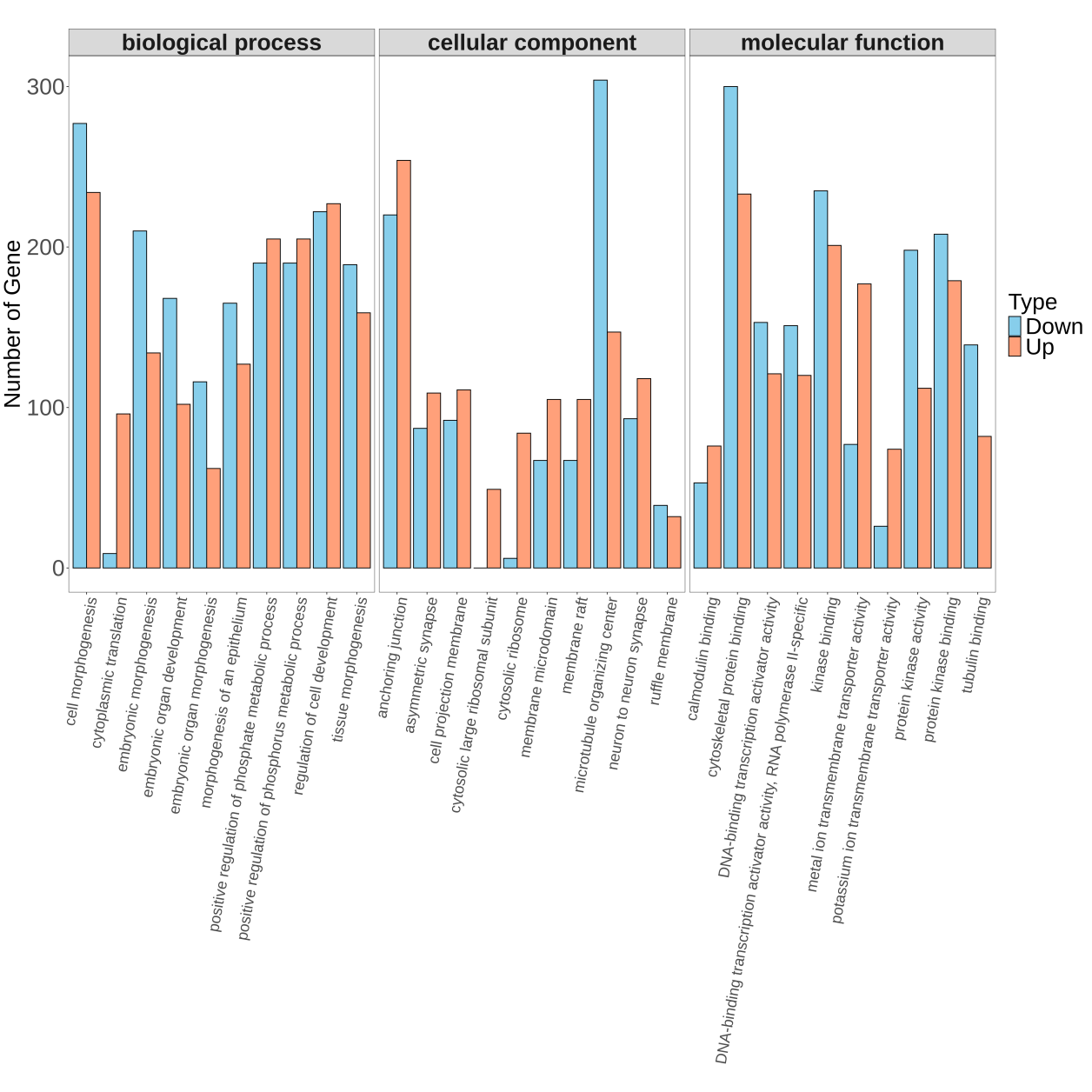


**Figure S34.** Stratified GO enrichment analysis of up- and down-regulated differentially expressed genes.


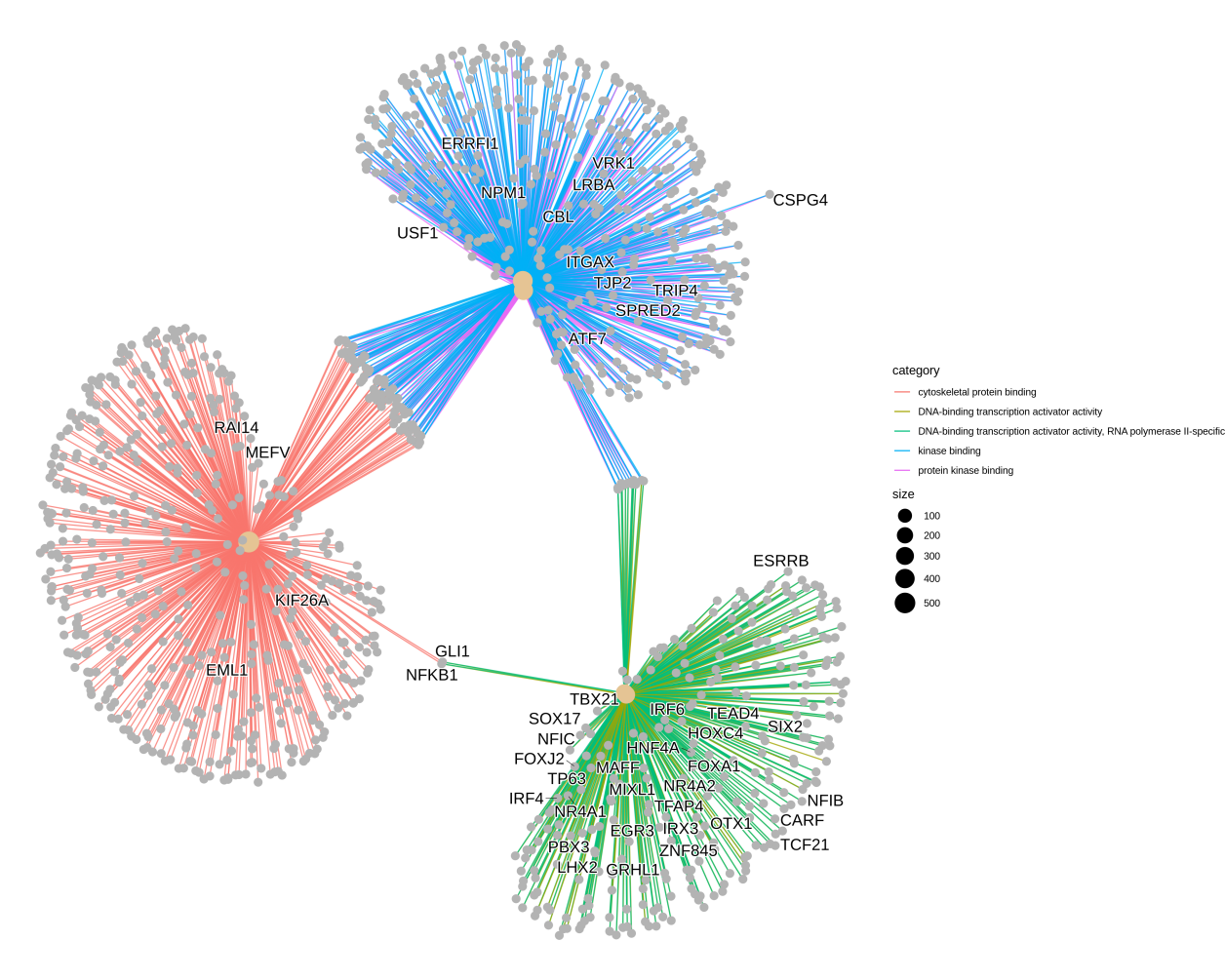


**Figure S35.** GO Gene-Term Association Network (Molecular Function).


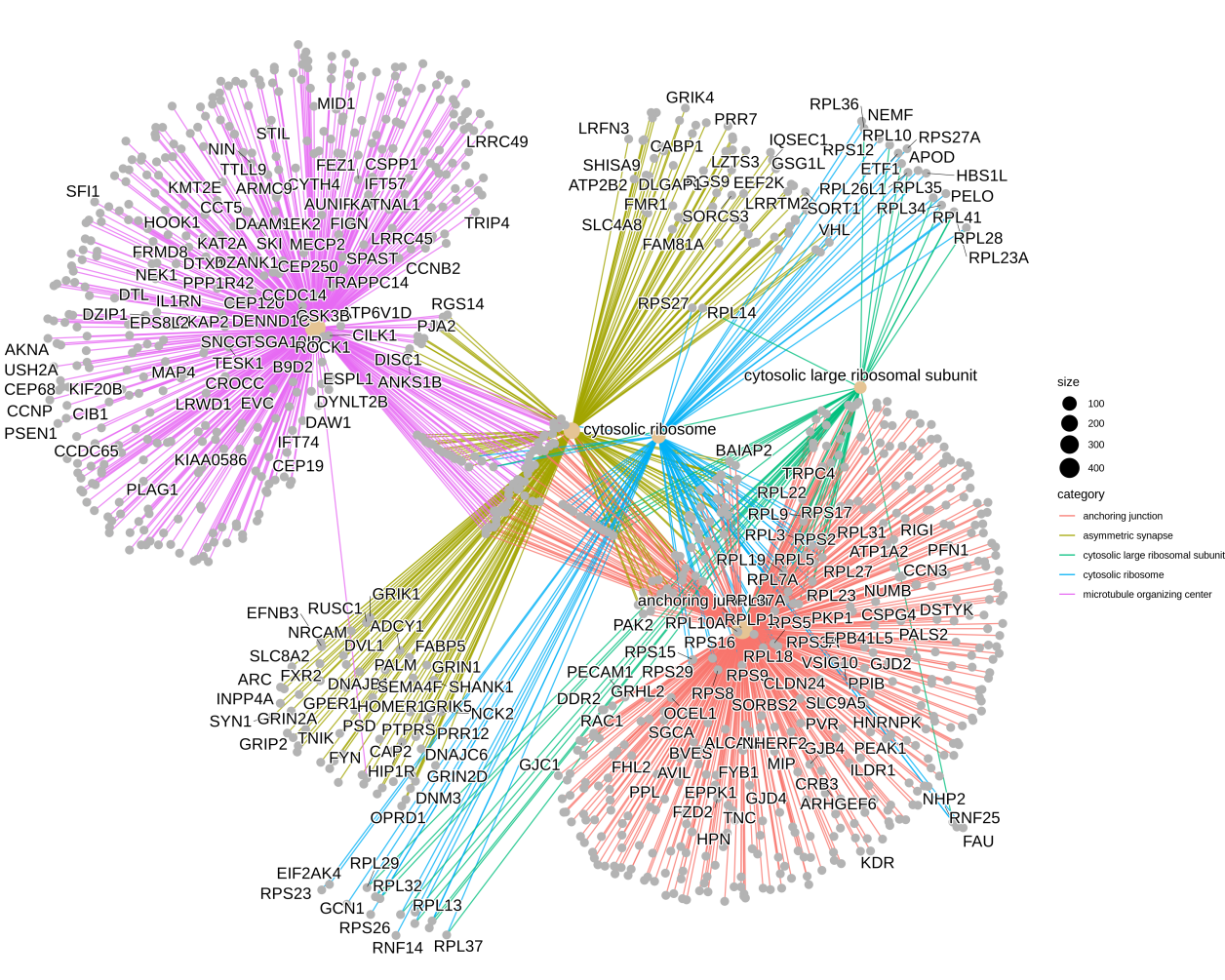


**Figure S36.** GO Gene-Term Association Network (Cellular Component).


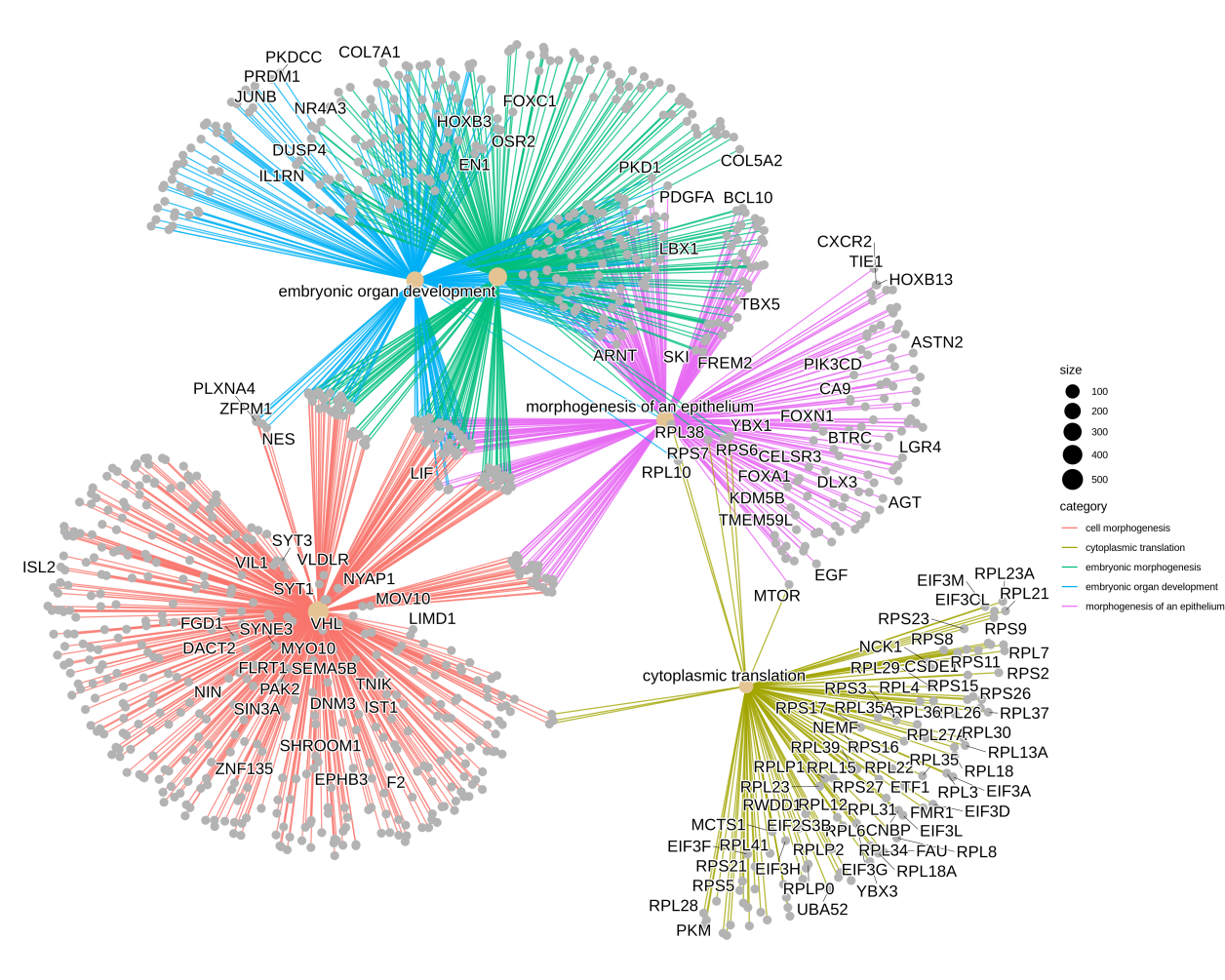


**Figure S37.** GO Gene-Term Association Network (Biological Process).


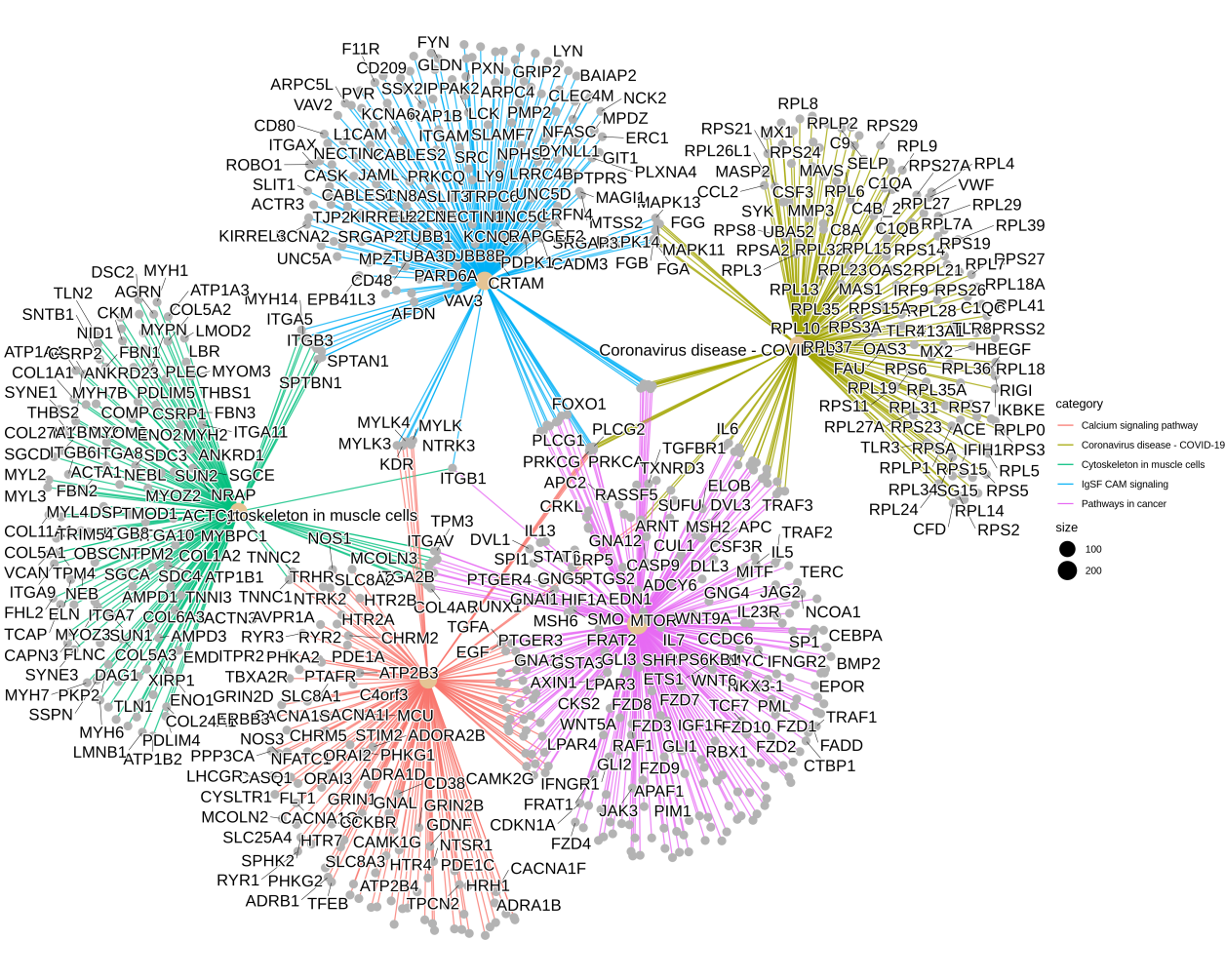


**Figure S38.** KEGG Gene-Pathway Association Network.


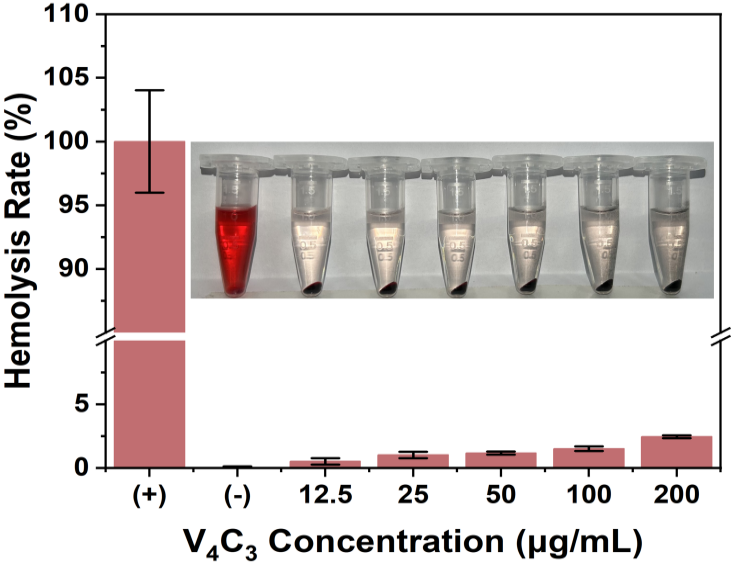


**Figure S39.** Hemolysis rate of VARH at different concentrations.


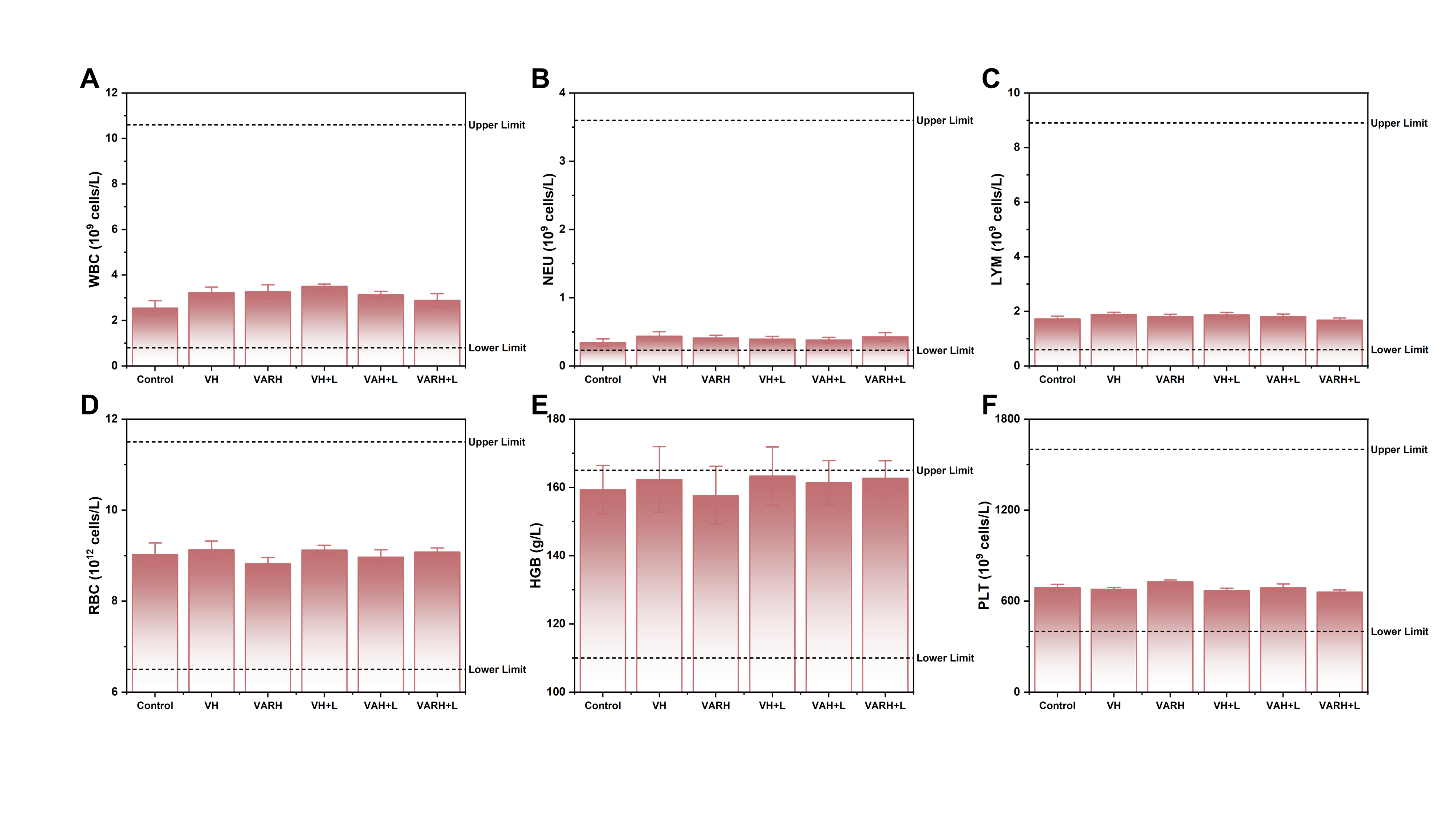


**Figure S40.** Analysis of complete blood count was performed on mice subjected to various treatments.


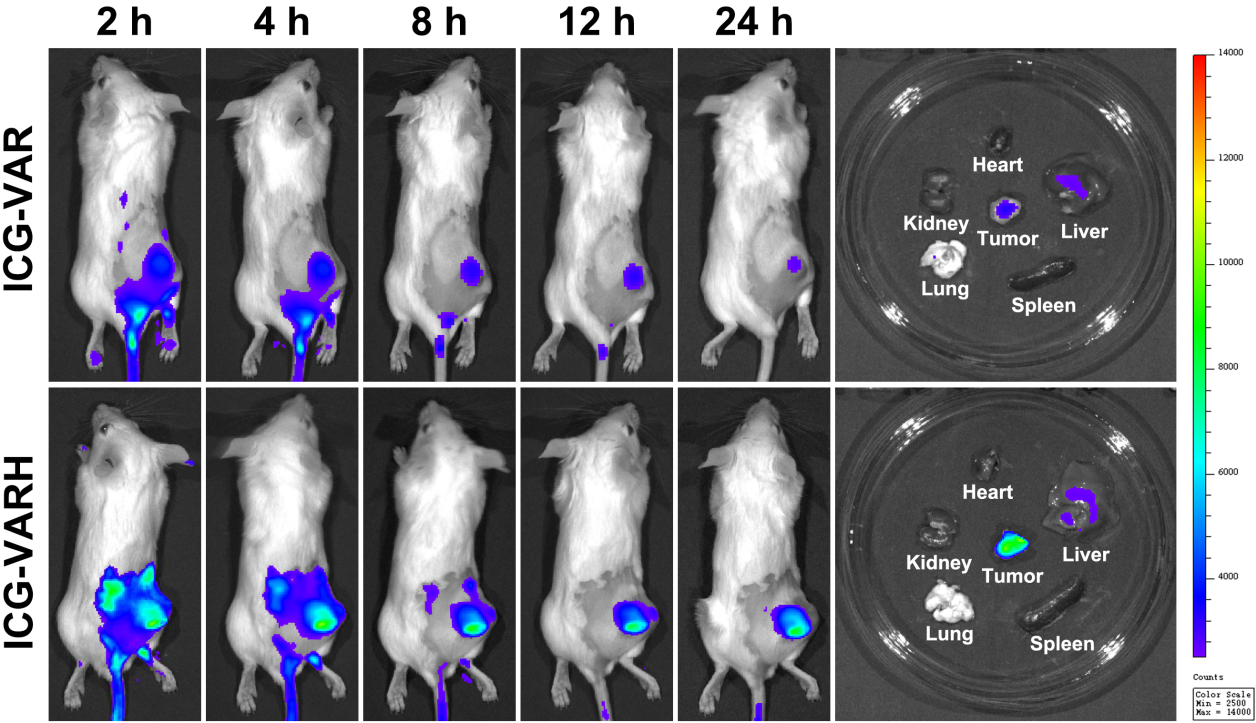


**Figure S41.** In vivo biodistribution and ex vivo fluorescence imaging of VAR and VARH nanocomposites labeled with ICG after tail vein injection in H22 tumor-bearing mice.


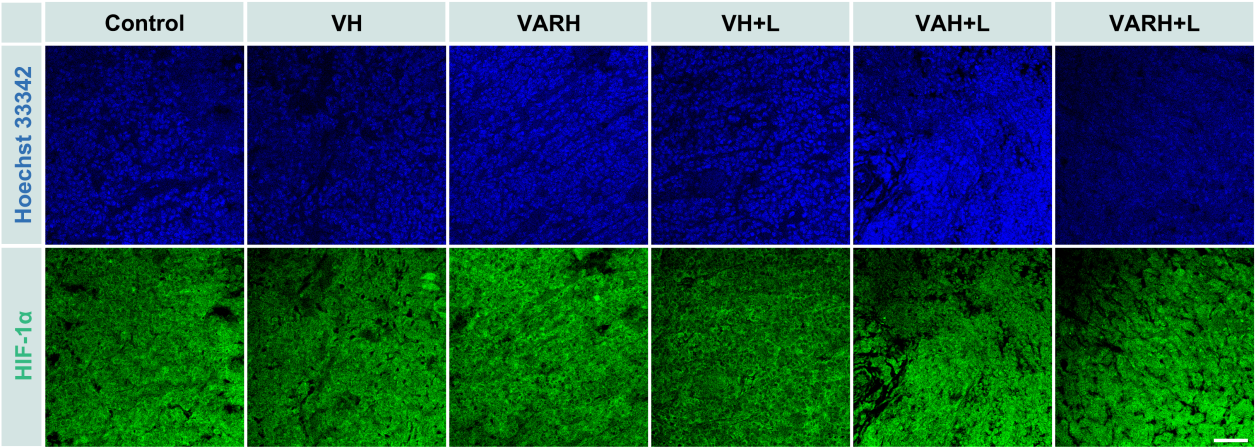


**Figure S42.** Representative CLSM images of HIF-1α immunohistochemistry in mouse tumor tissues under different treatments; scale bar = 50 μm.


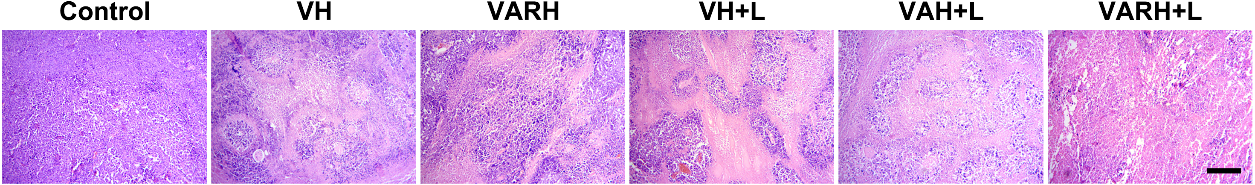


**Figure S43.** H&E-stained images of tumor tissues, scale bar = 100 μm.


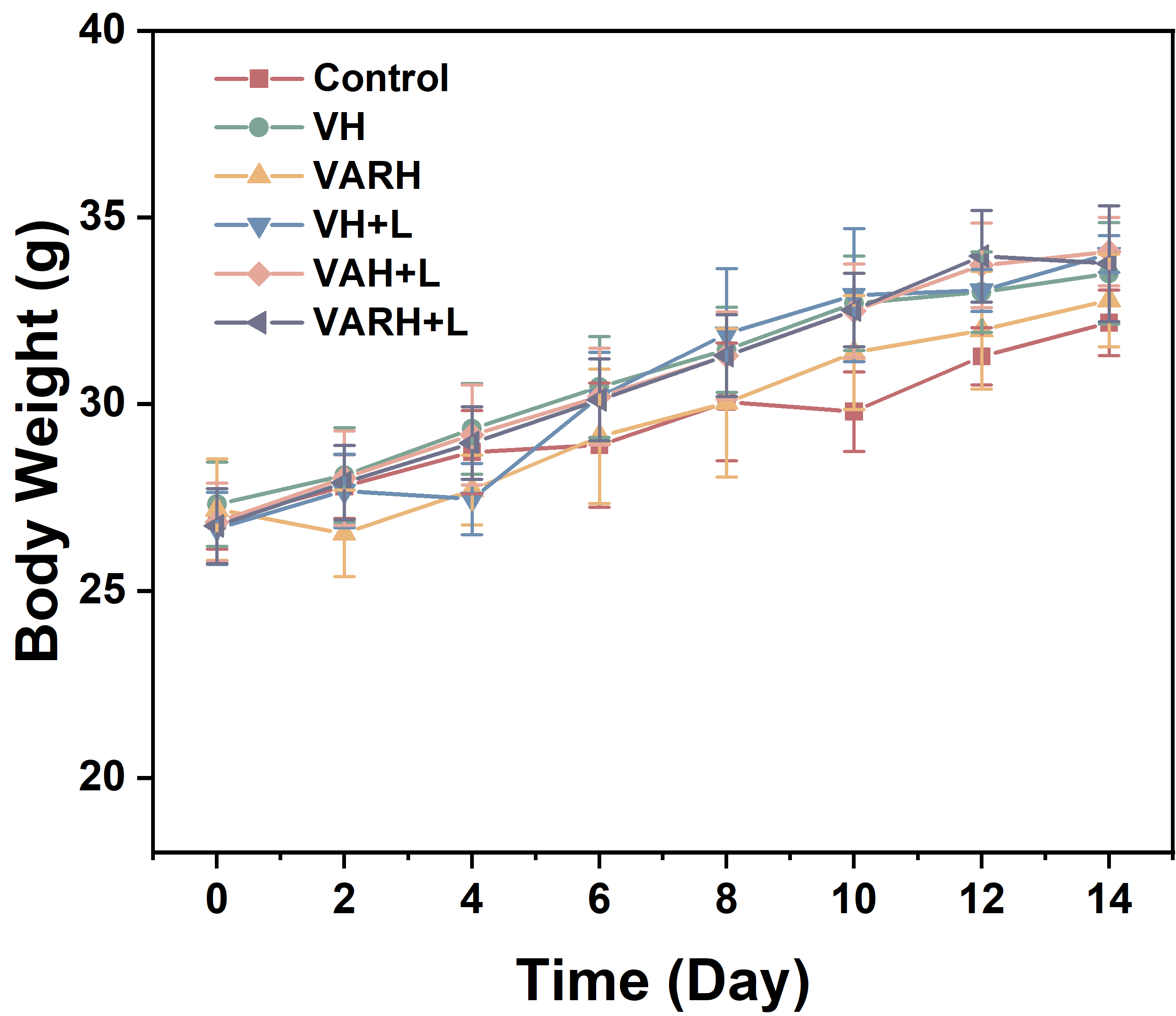


**Figure S44.** Body weight changes of H22 tumor-bearing mice during treatment in different groups.


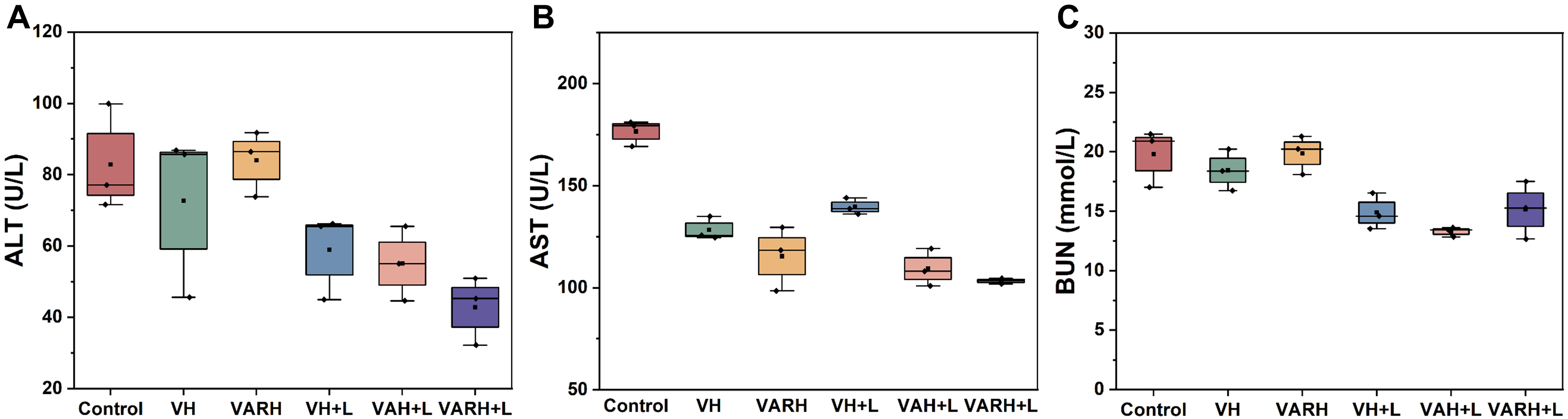


**Figure S45.** Changes in Blood Biochemical Indicators (A) ALT, (B) AST, and (C) BUN in Mice of Different Groups After the End of Treatment.


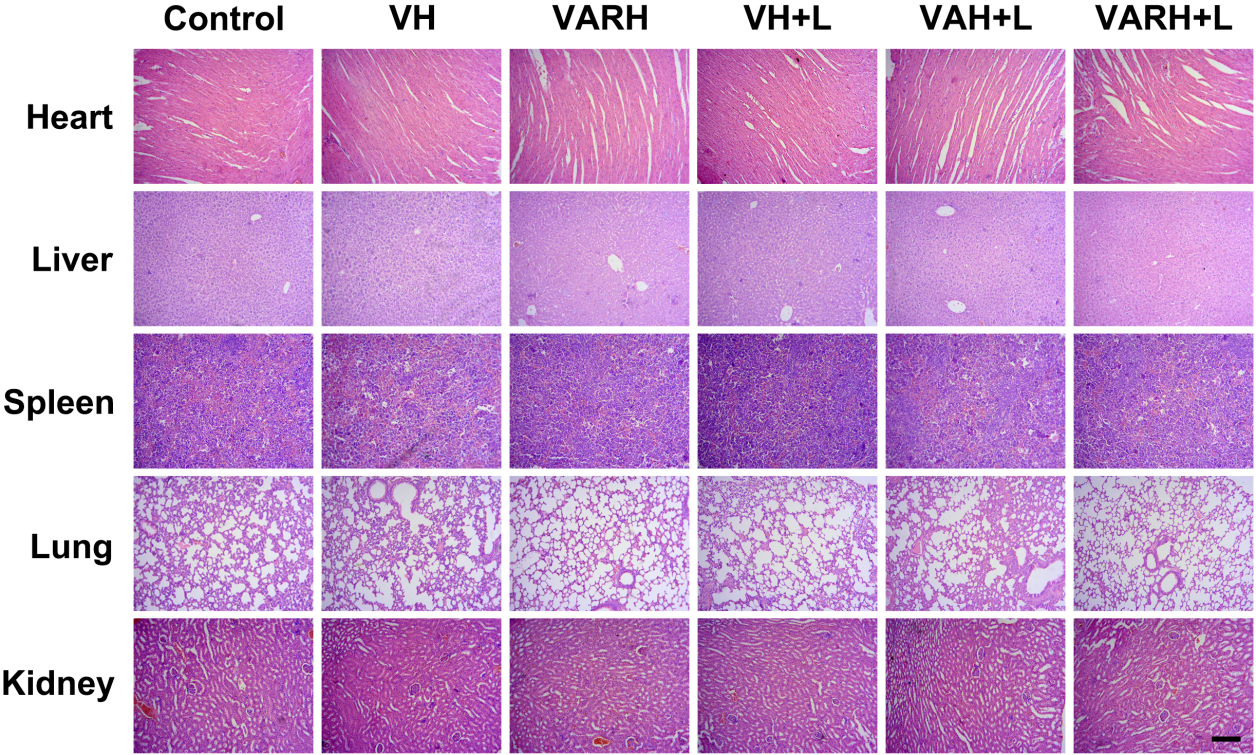


**Figure S46.** H&E-stained images of major organs (heart, liver, spleen, lung, kidney) from H22 tumor-bearing mice in different treatment groups, scale bar = 100 μm.


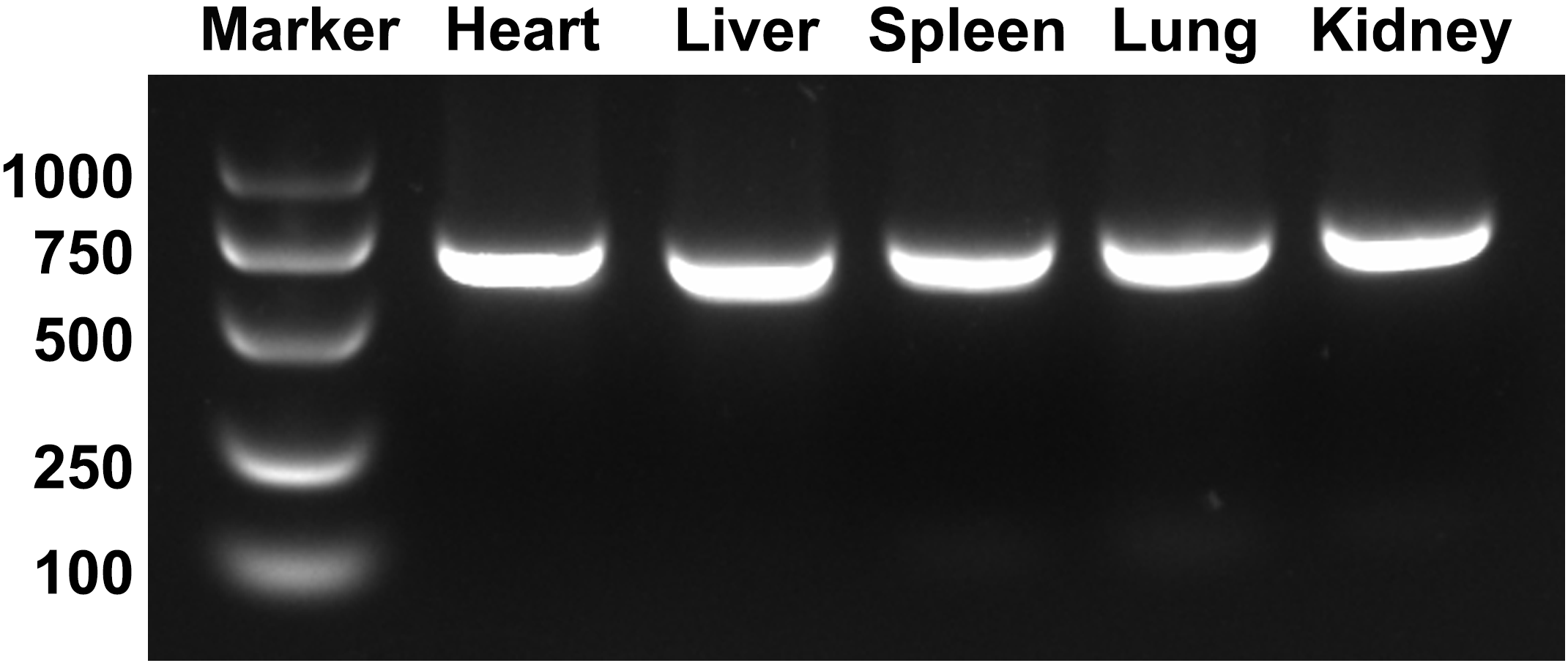


**Figure S47.** T7E1 assay for assessing off-target gene editing in five major organs.


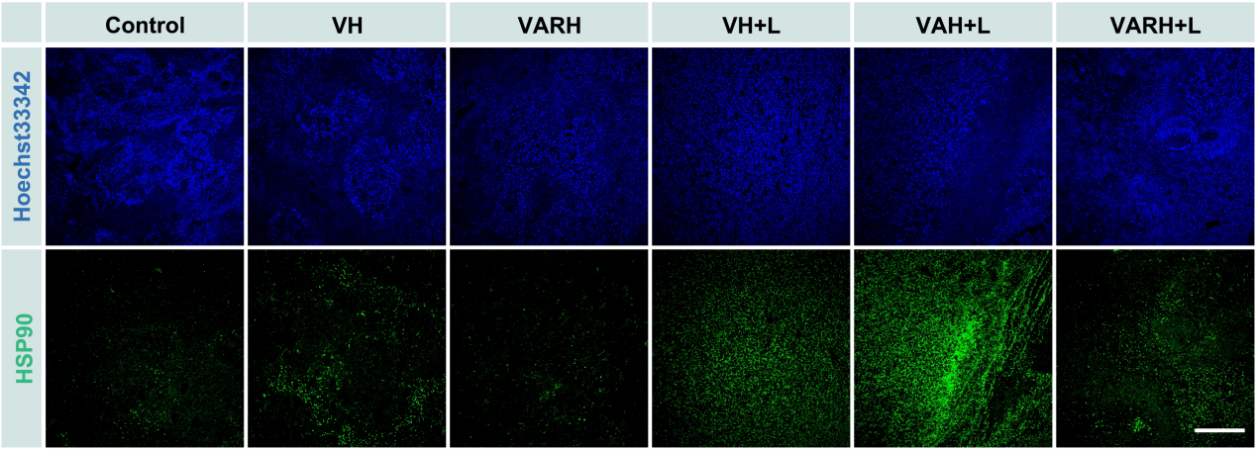


**Figure S48.** Immunofluorescence images of HSP90 protein in tumor tissues of tumor-bearing mice after treatment with different therapeutic groups. Scale bar = 200 μm.


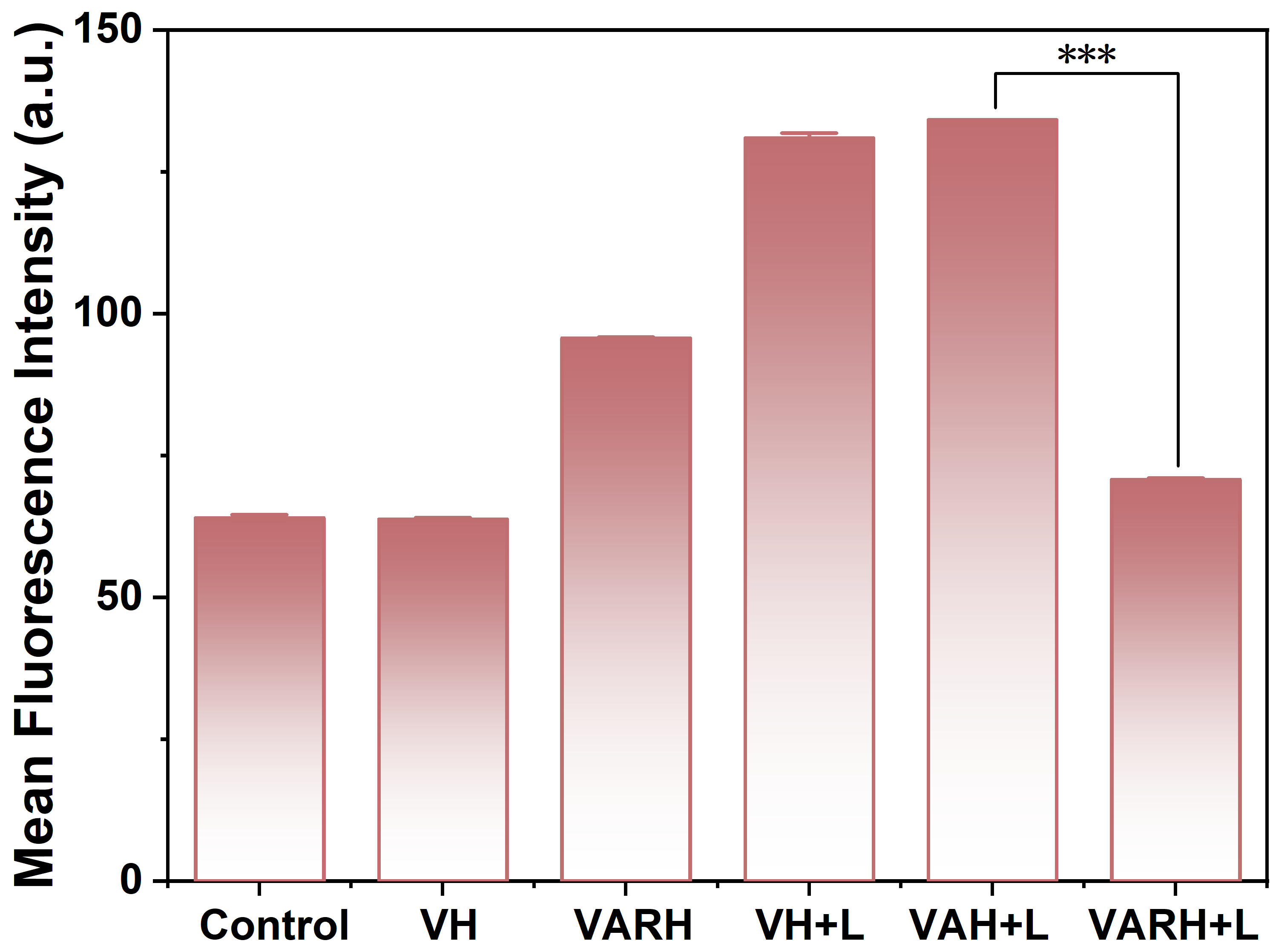


**Figure S49.** Immunofluorescence Quantitative Analysis of HSP90 Protein in Tumor Tissues of Tumor-Bearing Mice After Treatment with Different Treatment Groups.


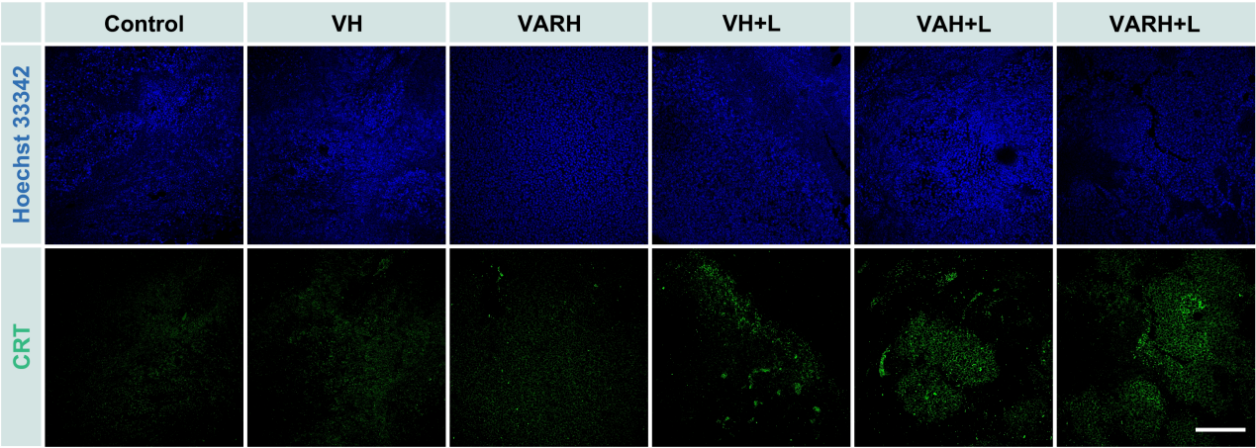


**Figure S50.** CRT exposure in tumor tissues of tumor-bearing mice after treatment with different groups, scale bar = 200 μm.


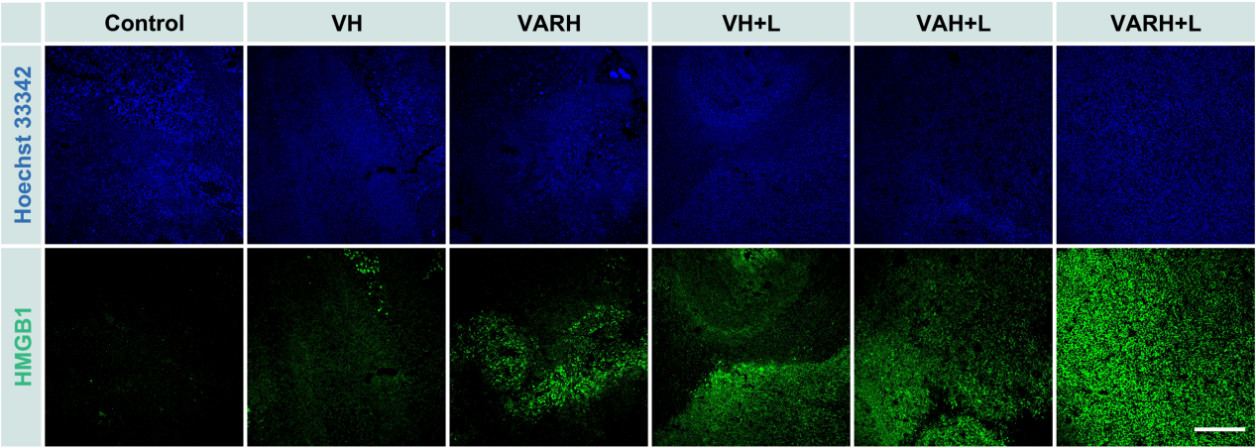


**Figure S51.** HMGB1 release in tumor tissues of tumor-bearing mice after treatment with different groups, scale bar = 200 μm.
